# Simple and effective serum-free medium for sustained expansion of bovine satellite cells for cell cultured meat

**DOI:** 10.1038/s42003-022-03423-8

**Published:** 2022-06-02

**Authors:** Andrew J. Stout, Addison B. Mirliani, Miriam L. Rittenberg, Michelle Shub, Eugene C. White, John S. K. Yuen, David L. Kaplan

**Affiliations:** 1 Biomedical Engineering Department, Tissue Engineering Resource Center, Tufts University, Medford, MA USA; 2 Biological Engineering Department, Massachusetts Institute of Technology, Cambridge, MA USA; 3 Tufts Veterinary Field Service, Cummings School of Veterinary Medicine, Tufts University, North Grafton, MA USA

**Keywords:** Cell growth, Stem-cell biotechnology, Muscle stem cells, Agriculture

## Abstract

Cell-cultured meat offers the potential for a more sustainable, ethical, resilient, and healthy food system. However, research and development has been hindered by the lack of serum-free media that enable the robust expansion of relevant cells (e.g., muscle satellite cells) over multiple passages. Recently, a low-cost serum-free media (B8) was described for pluripotent stem cells. Here, B8 is adapted for bovine satellite cells through the addition of a single component, recombinant albumin, which renders it suitable for long-term satellite cell expansion without sacrificing myogenicity. This new media (Beefy-9) maintains cell growth over the entire period tested (seven passages), with an average doubling time of 39 h. Along with demonstrated efficacy for bovine cells, Beefy-9 offers a promising starting-point for developing serum-free media for other meat-relevant species. Ultimately, this work offers a foundation for escaping cultured meat research’s reliance on serum, thereby accelerating the field.

## Abstract (web summary)

A substitution of FBS in culturing of bovine satellite cells for the purpose of culturing meat is presented, addressing both basal media and growth factors in relation to proliferating and differentiating cells.

## Introduction

Cell-cultured meat is an emerging technology which offers both promising possibilities and substantial scientific challenges. The promise of cultured meat lies in its potential to address environmental, ethical, and human health issues that plague intensive animal agriculture^1^. For instance, early life-cycle analyses suggest that cultured meat could require >90% less land and >75% less water than conventional beef, while contributing >75% fewer greenhouse gas emissions, >95% less eutrophication, and >90% less particulate matter formation^2,3^. At the same time, cultured meat could improve animal welfare, food-system resilience, and human health outcomes^4–6^. The challenges that face the successful technological transition of cultured meat to the marketplace stem from the need for production systems that are low-cost, scalable, food-safe, and free of animal-derived inputs^4,7,8^. Here, cell culture media is a particularly problematic hurdle for several reasons. First, media comprises the majority (>99%) of the cost of current production systems^7–9^. Second, the culture of meat-relevant cells, such as bovine satellite cells (BSCs), has traditionally relied on fetal bovine serum (FBS), a notoriously expensive, unsustainable, and inconsistent component, which is inherently antithetical to the aims of cultured meat^4^. Finally, when serum-free media for satellite cell expansion have been explored, they are either complex^10^, ineffective compared to serum-containing media^11^, reliant on proprietary or animal-derived additives^10–12^, or contain components (e.g., synthetic steroids) that could raise regulatory concerns^12^. Further, no serum-free media has been validated for the sustained expansion of satellite cells over multiple passages^10–12^. As such, despite the substantial body of work that has gone into the exploration of satellite cell culture systems^10–13^, a food-safe and fully animal-derived component-free medium remains a crucial limitation for the field.

Recently a low-cost serum-free medium was described for human iPSCs^14^. This media (B8) contains a simple mixture of basal media (glucose, amino acids, vitamins, salts, and fatty acids), minerals, and proteins, all of which can be found in animal tissue; as such, it is likely to be producible in a food-safe manner. Lab-scale B8 production was demonstrated at a cost of ~$16/L when using in-house growth-factor production. This cost could likely further be reduced with process scale-up and optimization. In contrast, media containing 20% serum or commercially available serum-free media that have been explored for satellite cells cost ~$200–500/L^11,14^. Because of its simplicity and cost, B8 offers a promising starting point for an effective serum-free medium that could advance cultured meat research.

Herein is validated a simple, B8-inspired serum- and animal-component-free media, termed Beefy-9, for culturing BSCs. First, it is shown that supplementation with just one animal-free component (recombinant human albumin, expressed in rice) makes Beefy-9 media effective for BSC expansion in vitro, and that short-term growth is comparable to that in 20% FBS. Next, a protocol for passaging BSCs in Beefy-9 is established and shown to maintain cellular myogenicity in serum-free conditions. Third, cost-saving opportunities for Beefy-9 are demonstrated by lowering growth-factor concentrations without a drastic reduction in proliferation. Fourth, long-term expansion and sustained myogenicity of BSCs are validated in fully serum-free conditions. Finally, the performance/cost ratio of Beefy-9 is optimized by tuning recombinant albumin concentrations to generate an additional medium termed Beefy-9+. Together, these findings offer a valuable tool for cultured meat researchers, and a promising foundation for further media optimization to enable low-cost, scalable, and sustainable cultured meat production.

## Results

### B8 media can lower but not eliminate serum requirements for BSCs during short-term growth

**Fig. 1:**
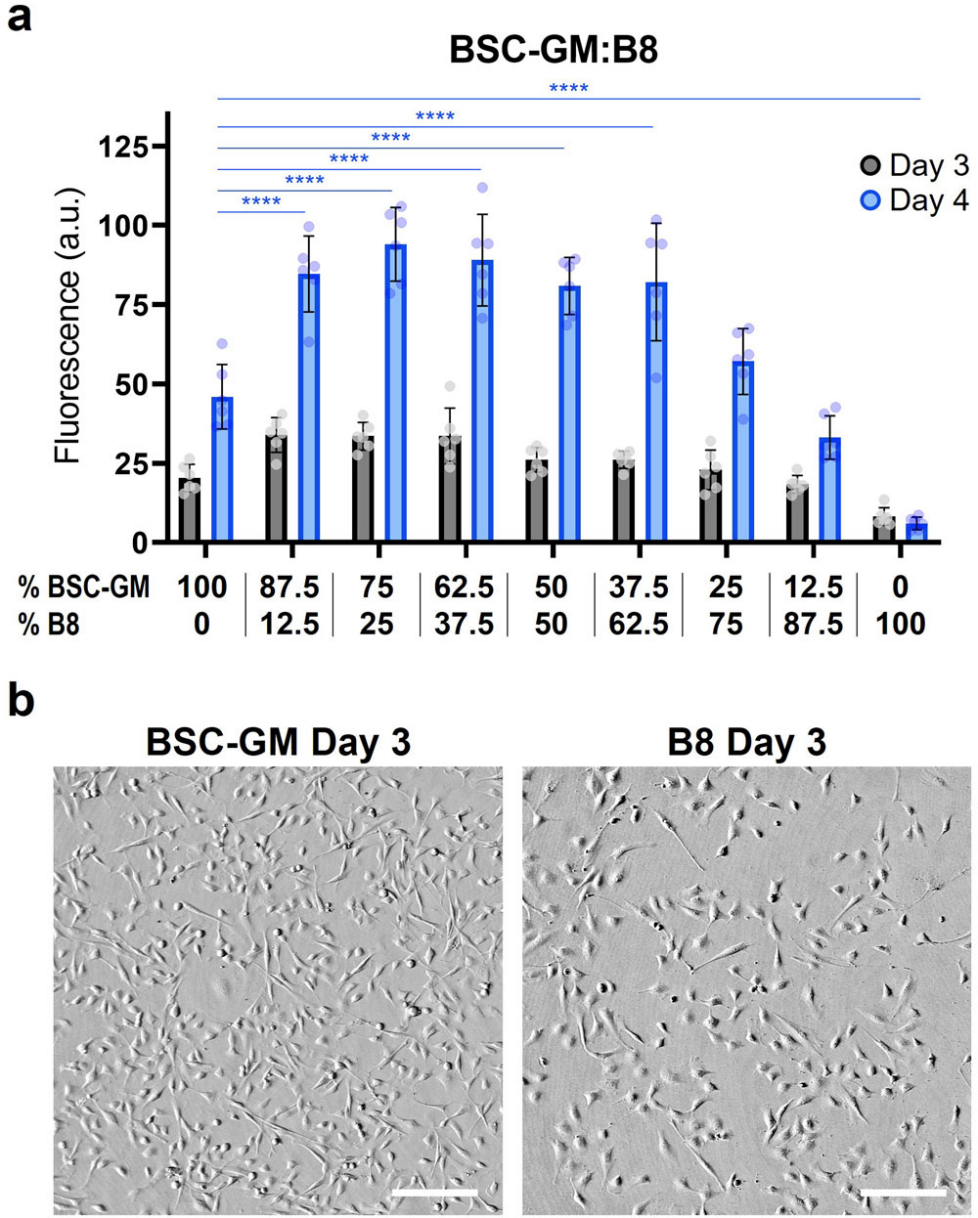
Short-term growth in BSC-GM mixed with B8. **a** BSC proliferation over 3 & 4 days in mixtures of BSC-GM (20% FBS) and B8 media. At the four-day timepoint, mixtures of up to 62.5% B8 significantly improved growth compared to BSC-GM alone, and mixtures of up to 87.5% B8 did not significantly reduce growth (*p* = 0.27). B8 alone showed a significant reduction in growth over four days compared with BSC-GM alone, and showed stagnating growth after three days. *n* = 6 distinct samples; statistical significance was calculated by one-way ANOVA on day 4 data comparing all samples with BSC-GM controls, and is indicated by asterisks, in which *p* < 0.05 (*), *p* < 0.01 (**), *p* < 0.001 (***), and *p* < 0.0001 (****). **b** Brightfield images of BSCs grown for three days in BSC-GM or B8 media. Images show that cell morphology was consistent in serum-containing or serum-free conditions. Cell confluency in the images is qualitatively consistent with growth analysis in part (**a**). Scale bars are 200 μm.

Bovine satellite cells were used throughout experiments. First, staining for Pax7 and Myosin Heavy Chain (MHC) were performed before and after differentiation of isolated cells in order to verify the initial and terminal states of these stem cells^15,16^. Quantitative image cytometry revealed that 96.3% of isolated cells were positive for Pax7 (Fig. S1), indicating a suitably pure population of satellite cells. After cell identity was verified, B8’s capacity to replace serum containing media was analyzed via short term BSC growth assays (3 and 4 days) in mixtures of standard growth media containing 20% FBS (BSC-GM) combined with homemade B8 or supplier provided HiDef-B8 (Figs. 1 and S2, Table S1). These timepoints were selected to maintain a cellular confluency of less than or equal to 70% throughout the experiment. The results showed that B8 media mixed with BSC-GM significantly improved growth compared to BSC-GM alone over four days (Fig. 1a), and that this benefit remained with as much as a 62.5% reduction in FBS (62.5% B8 media). Additionally, a serum reduction of 87.5% did not significantly reduce cell growth over four days. On the other hand, while B8 media alone encouraged cell growth over three days (Fig. 1a, b), this growth did not continue into day 4. These results indicated that B8 was capable of reducing serum requirements in BSCs, but could not completely eliminate these requirements.

### B8 media supplementation improves cell proliferation

**Fig. 2:**
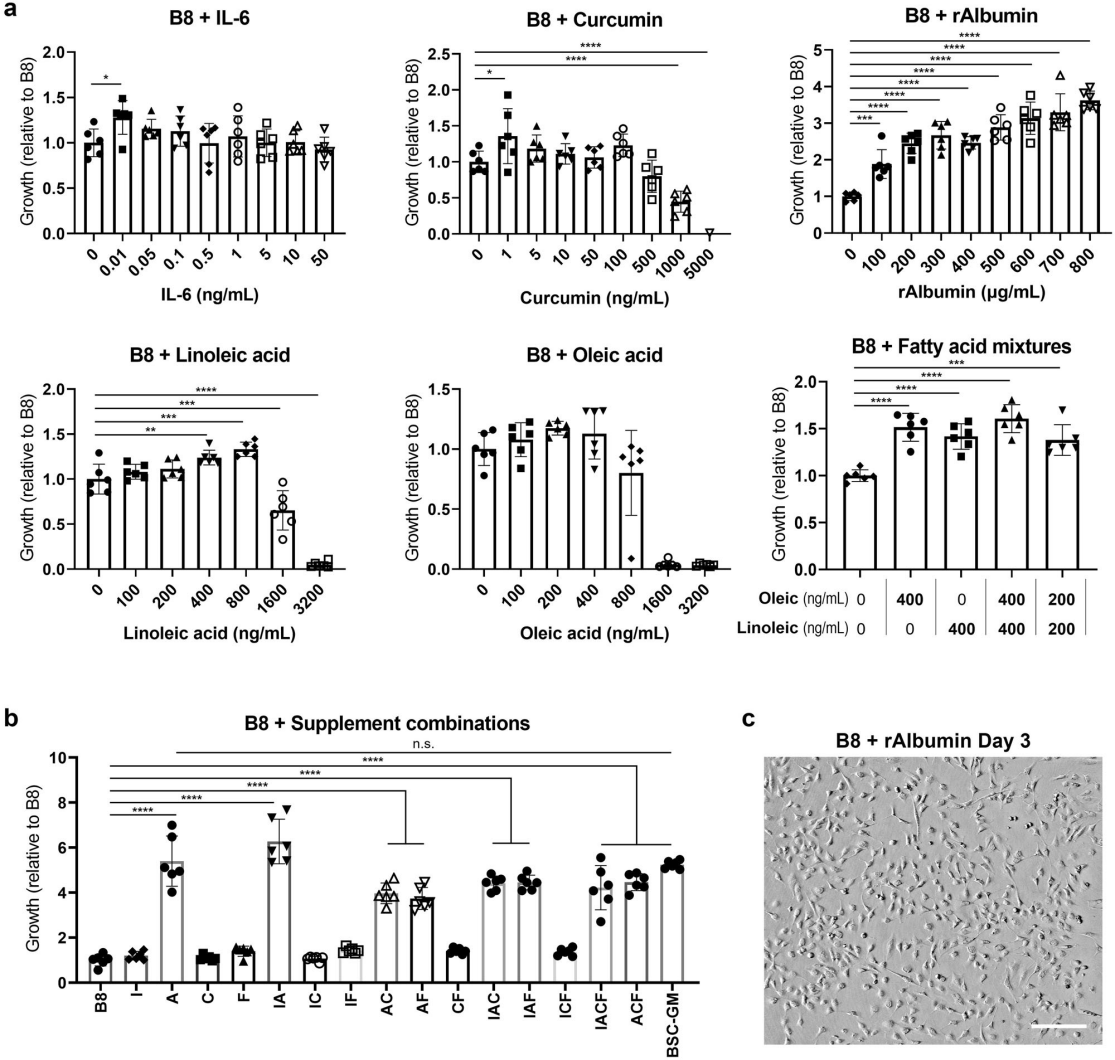
Short-term growth in supplemented B8 media. **a** BSC proliferation over 4 days B8 supplemented with interleukin 6 (IL-6), curcumin, recombinant albumin (rAlbumin), linoleic acid, oleic acid, or mixtures of linoleic and oleic acid. Growth was analyzed on day 4 via dsDNA quantification, and values are given relative to B8. *n* = 6 distinct samples; statistical significance was calculated by one-way ANOVA with multiple comparisons between supplemented samples and B8 controls, and is indicated by asterisks, in which *p* < 0.05 (*), *p* < 0.01 (**), *p* < 0.001 (***), and *p* < 0.0001 (****). While no significant difference was found for oleic acid on day 4 when tested alone (bottom middle panel), there was significant difference on day 3 (Fig. S3), and so it was included as part of the fatty acid mixture analysis (bottom right panel). **b** BSC proliferation over 4 days in B8 supplemented with combinations of factors, in which: B8 = B8; I = IL-6 (0.01 ng/mL); A = rAlbumin (800 μg/mL); C = Curcumin (1 ng/mL); F = linoleic acid (400 ng/mL) and Oleic acid (400 ng/mL); and BSC-GM = serum-containing growth media. *n* = 6 distinct samples; statistical significance was calculated by one-way ANOVA with multiple comparisons between all samples, and is indicated by asterisks, in which *p* < 0.05 (*), *p* < 0.01 (**), *p* < 0.001 (***), and *p* < 0.0001 (****). Statistically significant differences between B8 and other samples are shown, as is the lack of significance between rAlbumin supplemented B8 and BSC-GM (*p* > 0.9990). **c** Brightfield imaging of BSCs at day 4 in B8 with rAlbumin (800 μg/mL) shows that cell morphology was maintained in B8 with albumin supplementation compared with images in Fig. 1. Scale bar is 200 μm.

To overcome the deficiencies of B8 media alone, numerous supplements were tested in a range of concentrations (full list in Table S2), and growth was again analyzed over four days. Six of the factors significantly improved BSC proliferation compared to B8 media alone (Figs. 2a and S3). These were interleukin-6 (IL-6), curcumin, recombinant human albumin (rAlbumin), platelet-derived growth factor (PDGF-BB), linoleic acid, and oleic acid. Of these, rAlbumin was particularly effective, imparting a ~4-fold improvement in growth compared with plain B8. In contrast, other supplements resulted in at best only a ~50% improvement compared with plain B8. To test whether combinations of these supplements could offer synergistic benefits to cell growth, optimal concentrations of the above factors (with the exception of PDGF-BB, which was determined to be insufficiently effective for the substantial cost) were combined and tested (Fig. 2b). Here, rAlbumin (800 μg/mL) was the driving factor in all significant improvements. While a combination of IL-6 (0.01 ng/mL) and rAlbumin offered slightly improved growth compared with rAbumin alone, this difference was not statistically significant. In the interest of maximizing media simplicity and minimizing cost, an augmented B8 media with nine components was established by supplementing with 800 μg/mL rAlbumin alone. This media was termed Beefy-9 due to its component number and design towards bovine muscle cell culture. This media was capable of maintaining short-term growth comparable to serum-containing BSC-GM and maintaining cell morphology in vitro (Fig. 2b, c).

### Passaging in Beefy-9 media

**Fig. 3:**
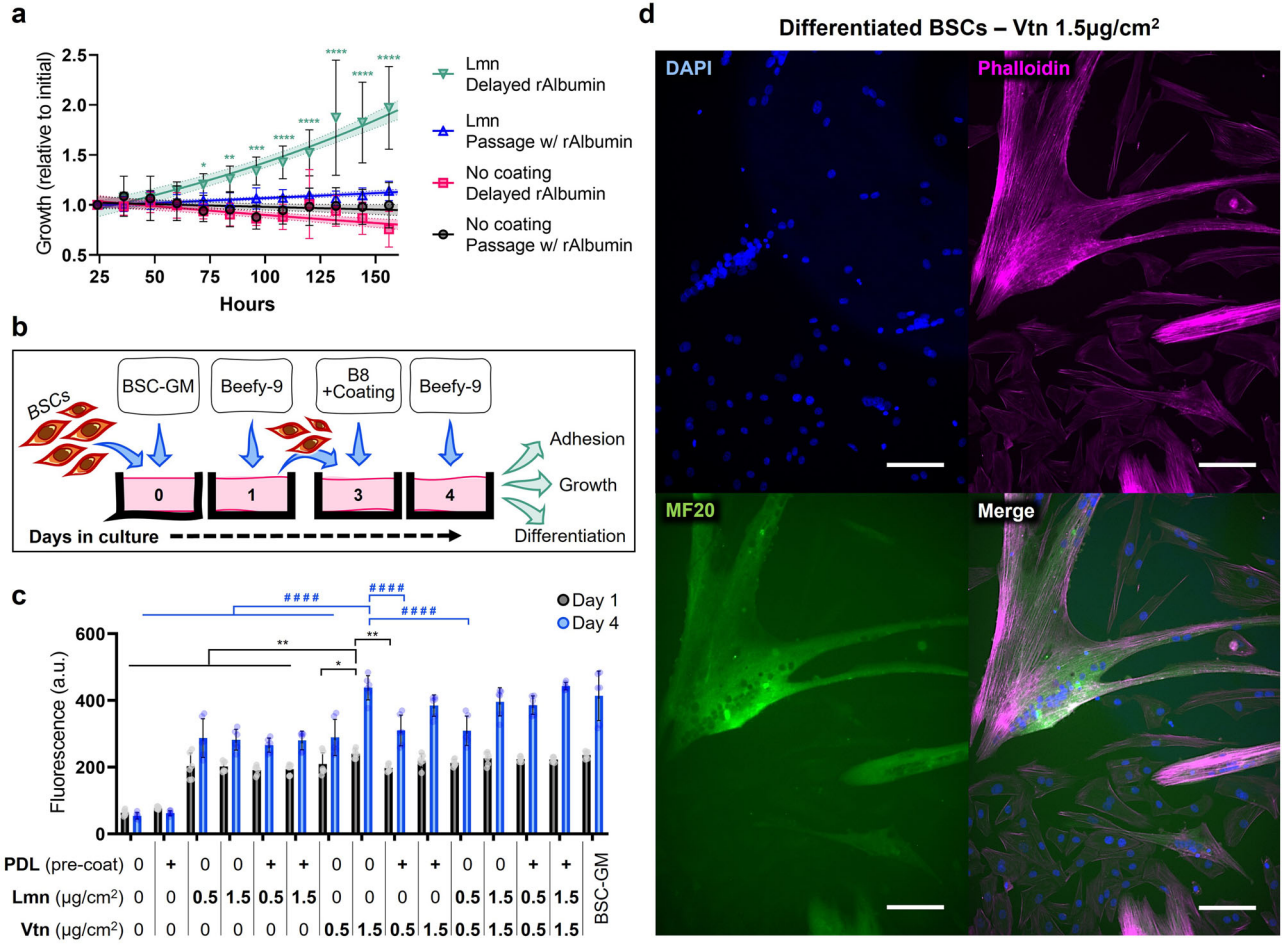
Passaging in Beefy-9. **a** Growth analysis via live-cell imaging of BSCs passaged in B8/Beefy-9 media. Results showed that cells needed to be passaged in the absence of supplemental rAlbumin (Delayed rAlbumin), and that a coating (e.g., iMatrix-511 laminin) was required for adhesion and growth. Specifically, cells without any coating (No coating) were unable to grow, as were cells with iMatrix-511 (Lmn) coating that were passaged in the presence of rAlbumin (Lmn; Passage w/ rAlbumin). In contrast, cells that were passaged onto Lmn coated flasks and allowed to adhere overnight before the addition of rAlbumin (“Lmn Delayed rAlbumin”) showed exponential growth. *n* = 9 image fields of view; statistical significance calculated by two-way ANOVA with multiple comparisons between conditions, and significant difference between “Lmn Delayed rAlbumin” and all other conditions are indicated by asterisks, in which *p* < 0.05 (*), *p* < 0.01 (**), *p* < 0.001 (***), and *p* < 0.0001 (****). A 95% confidence interval calculated via nonlinear regression (least squares regression; exponential (Malthusian) growth) is given. **b** Schematic of B8/Beefy-9 passaging system developed. BSCs were plated in identical conditions (BSC-GM) on day 0 to ensure that an equal number of cells adhered to the plate initially. On day 1, cells were rinsed with PBS and media was changed to Beefy-9. At 70% confluency (day 3), cells were passaged using TrypLE and plated in B8 (no rAlbumin) along with adhesive peptides. One day after passaging, media was changed to Beefy-9, and cells were proliferated and analyzed for adhesion, growth, and myogenicity. **c** PrestoBlue adhesion and growth analysis of BSCs plated with various animal-free coatings. Truncated vitronectin (Vtn) at 1.5 μg/cm^2^ showed superior cell attachment and growth compared to iMatrix-511 laminin (Lmn) or Poly-D-Lysine (PDL). *n* = 6 (two reads for three biological replicates); statistical significance was calculated by one-way ANOVA performed separately for day 1 or day 4 with multiple comparisons between Vtn-N 1.5 μg/cm^2^ and all other samples, and is indicated by asterisks (day 1) or hashes (day 4), in which *p* < 0.05 (*, #), *p* < 0.01 (**, ##), *p* < 0.001 (***, ###), and *p* < 0.0001 (****, ####). **d** Immunofluorescence staining for nuclei (DAPI), actin (Phalloidin), and Myosin Heavy Chain (MF20) in BSCs passaged in Beefy-9 media with 1.5 μg/cm^2^ Vtn-N and delayed rAlbumin. Cells were proliferated to confluency and differentiated for 6 days in a previously described serum-free differentiation medium. Scale bars are 50 μm.

While short-term growth experiments were useful in establishing the benefits of rAlbumin supplementation for tailoring B8 media to short term BSC culture needs, long-term culture and passaging are essential for the robust cell expansion that is required for cultured meat. However, seeding cells into Beefy-9 media directly after passaging initially proved ineffective, as cells did not re-adhere to tissue culture plastic. Two possible explanations were hypothesized for this result. The first was that the coating used (0.25 μg/cm^2^ of laminin-511) was insufficient to enable cell adhesion in the absence of other adhesion factors present in serum (e.g., vitronectin and fibronectin)^17^. The second was that the high concentration of rAalbumin was outcompeting laminin for adsorption to the tissue-culture plastic, thereby further hindering cell adhesion^18,19^. To overcome these possible limitations, BSC passaging was explored 1) with the exclusion of rAlbumin until one day after passaging in order to allow cells to adhere to the flasks overnight, and 2) with various concentrations of different adhesive proteins. Because one priority of this work was media and cell culture workflow simplicity, focus was placed on recombinant adhesive proteins that were relatively low-cost and which had been demonstrated without pre-coating [e.g., laminin-511 fragment (iMatrix-511) and truncated vitronectin (Vtn-N)]^20,21^. Poly-D-Lysine (PDL) coatings (which can be purchased commercially on pre-coated plates) were also explored to augment cell attachment with or without adhesive peptides^21^. The results indicated that delaying the addition of rAlbumin was necessary to allow cell adhesion and growth, as was coating with a cell adhesive peptide (Fig. 3a). When comparing various peptides, results indicated that iMatrix-511 laminin was sub-optimal compared with Vtn-N (Fig. 3c). Specifically, 1.5 μg/cm^2^ of Vtn-N showed superior cell adhesion (day 1) and growth (day 4) than PDL alone, laminin alone, PDL + laminin, or a lower concentration of Vtn-N with or without PDL. Once a suitable passaging method was determined, short-term growth curves were again performed with Beefy-9 supplemented with various growth factors in order to rule out the possible confounding effects that adsorbed serum proteins might have had on short term growth curves performed previously (in which cells were seeded in the presence of serum). No significant effect of these factors was found (Fig. S4), reaffirming that supplementation with rAlbumin alone was optimal over multiple passages.

### Serum-free differentiation following expansion in Beefy-9 media

After establishing delayed rAlbumin and 1.5 μg/cm^2^ of Vtn-N as suitable parameters for multiple-passage culture of BSCs in Beefy-9, the myogenicity of expanded cells was verified. To do this, Beefy-9-passaged cells (P2) were expanded to confluency in Beefy-9 and differentiated for 5 days in a previously published serum-free differentiation medium^10^. Differentiated cells showed the formation of multinucleated myotubes which stained positive for the myogenic marker myosin heavy chain (MHC) (Fig. 3c). The results validated that myogenicity of cells cultured and passaged in Beefy-9 media was maintained. Together, these results demonstrate a fully animal-component-free culture system for proliferating, passaging, and differentiating BSCs (Supplementary Video 1).

### Cost-reducing strategies for Beefy-9 media

**Fig. 4:**
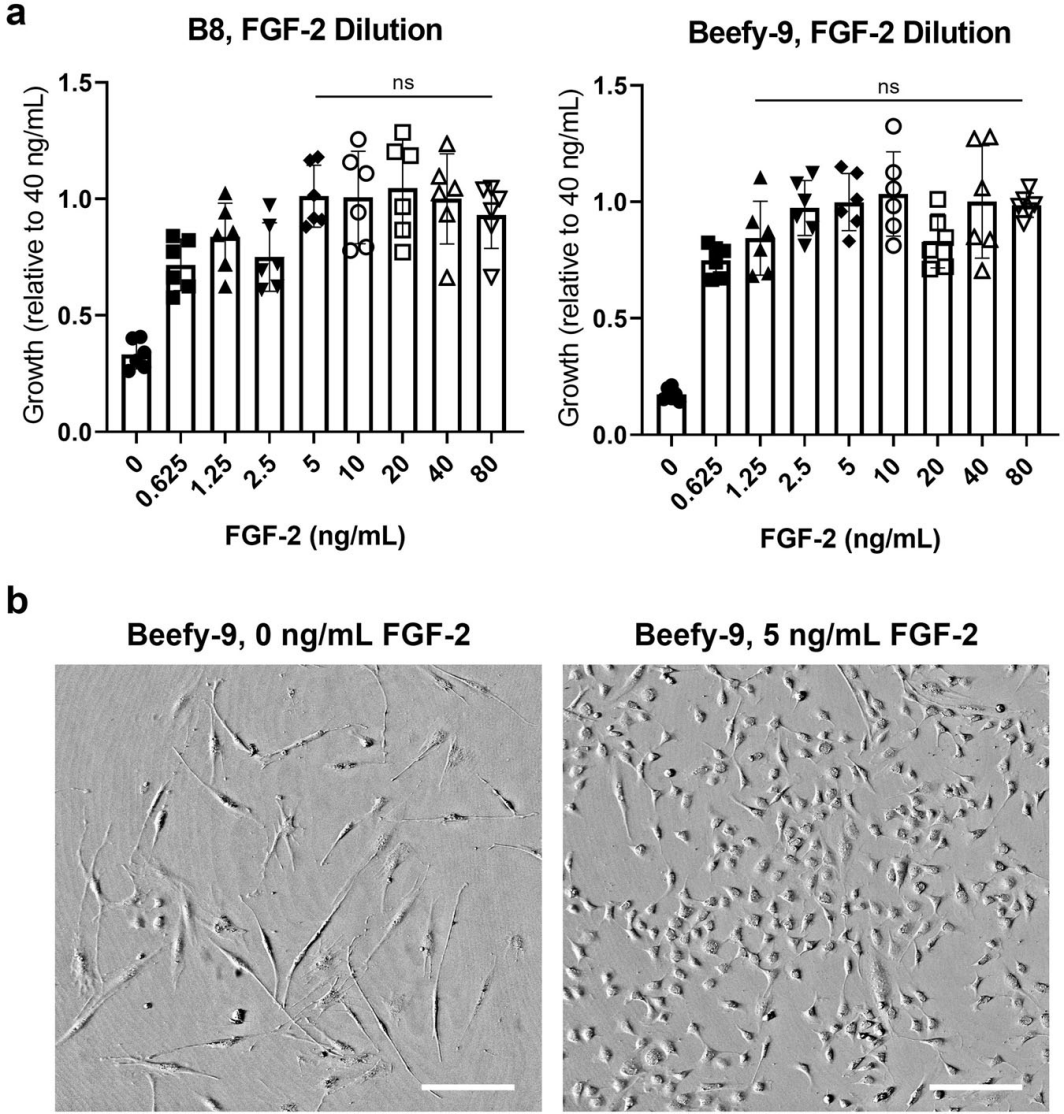
Short-term growth in B8 & Beefy-9 with reduced FGF-2. **a** BSC proliferation over 4 days in B8 or Beefy-9 with various concentrations of FGF-2. FGF-2 could be reduced to 5 ng/mL or 1.25 ng/mL in B8 or Beefy-9, respectively, without significantly impacting cell growth over four days. *n* = 6 distinct samples; statistical significance was calculated by one-way ANOVA with multiple comparisons between various FGF-2 concentrations and 40 ng/mL control conditions. Lack of significance between samples is indicated by ‘ns’ across all samples that hold no significant difference from 40 ng/mL). **b** Brightfield images of BSCs grown for three days in Beefy-9 media with 0 or 5 ng/mL FGF-2. Images show that the complete removal of FGF-2 from Beefy-9 significantly affects satellite cell morphology, whereas a reduction to 5 ng/mL did not affect morphology as compared with images in Figs. 1 and 2. Scale bars are 200 μm.

Next, simple cost reduction strategies for Beefy-9 were explored by lowering the concentration of FGF-2, which is a major cost contributor to B8 and Beefy-9 formulations at the baseline concentration of 40 ng/mL. Growth was analyzed over four days as before in B8 and Beefy-9 media with FGF-2 concentrations ranging from 0–80 ng/mL. The results showed that for B8 and Beefy-9, FGF-2 could be lowered to 5 ng/mL and 1.25 ng/mL, respectively, without significantly affecting growth (Fig. 4a). In contrast, cell growth and morphology were significantly affected by the complete removal of FGF-2 from the media (Fig. 4a, b). These results indicated that substantial reduction in FGF-2 is possible to lower the cost of Beefy-9 without negatively impacting short-term growth rates.

### Long-term culture in Beef-9 media

**Fig. 5:**
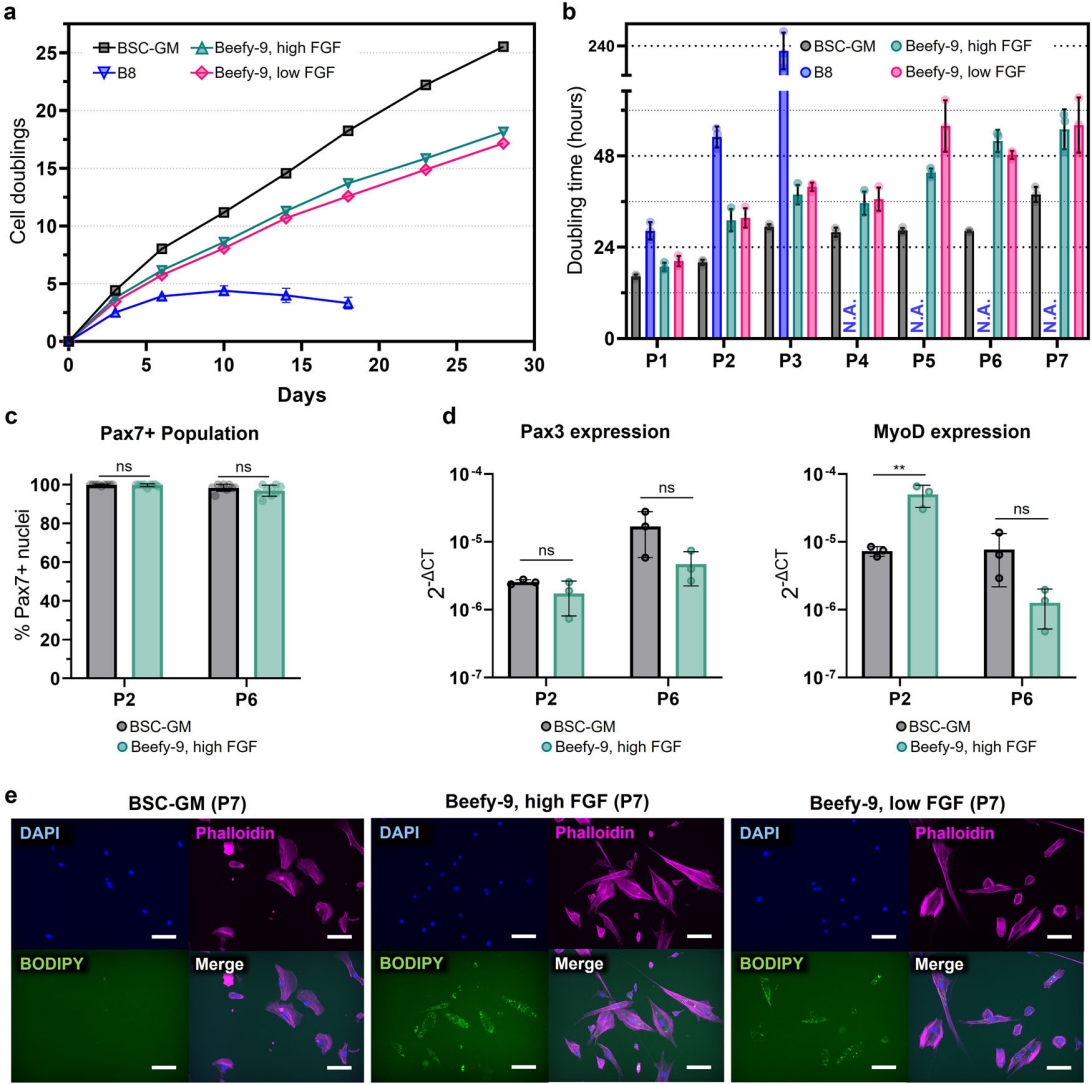
Long-term culture. **a** Cell doublings over multiple passages of BSCs cultured in BSC-GM, B8, Beefy-9, high FGF (40 ng/mL FGF-2), or Beefy-9, low FGF (5 ng/mL FGF-2). Results show that the addition of rAlbumin significantly improved cell growth in B8 over four weeks, although not to the degree of serum. Reducing Beefy-9 FGF-2 decreased cell doublings compared to 40 ng/mL, although this difference was less substantial (17.2 doublings vs 18.2 doublings at 28 days). *n* = 6 (two counts for three biological replicates), and error bars are given as ±standard deviation (though in some instances are smaller than the sample icons). **b** Doubling times were calculated over long-term culture and compared between media. An increase in doubling time at higher passages was found, particularly for Beefy-9 with high or low FGF-2. Notably, however, doubling times remained <48 h for the first five passages (~13 doublings) in Beefy-9 with high FGF-2. **c** Analysis of Pax7 staining (Fig. S5) shows that the percentage of Pax7+ nuclei stayed above 96% for at least six passages in both serum and serum-free media. No significant difference in the percent of Pax7+ cells is observed between media types (*n* = 9 distinct samples, calculated via two-way ANOVA with multiple comparisons between media types). **d** Quantitative PCRs for Pax3 and MyoD expression in proliferative cells grown in BSC-GM and Beefy-9 show no significant differences between media types, except in the case of P2 MyoD, where Beefy-9 shows higher expression than BSC-GM. Results are supported by immunofluorescent staining for these markers (Fig. S5). *n* = 3 distinct samples; statistical significance was calculated via two-way ANOVA with multiple comparisons between media and is indicated by asterisks in which *p* < 0.01 (**). **e** BODIPY staining of lipid accumulation in P7 cells cultured in various media. Cells were passaged, allowed to adhere overnight, and stained for nuclei (DAPI, blue), actin (Phalloidin, magenta), and lipids (BODIPY, green). Results show increased lipid accumulation in in serum-free media compared to BSC-GM. Scale bars 50 μm.

The last step after validating Beefy-9 for short-term growth and establishing an appropriate passaging protocol was to validate long-term expansion of BSCs in Beefy-9. BSCs were seeded as before and fed with either serum-containing BSC-GM, B8, Beefy-9 with high FGF-2 (40 ng/mL), or Beefy-9 with low FGF-2 (5 ng/mL). The low FGF-2 concentration was conservatively selected as the concentration that did not significantly affect short-term growth in either B8 or Beefy-9. Cells were cultured and passaged as described (Fig. 3b) for seven passages over 28 days, with cell counts used to determine cumulative cell doublings over the four-week period. While BSC-GM was still the optimal media over a long growth period, Beefy-9 with high or low FGF-2 content showed significant improvements over B8 media without the addition of rAlbumin (Fig. 5a). Indeed, BSCs in B8 alone ceased proliferating after three passages (4.4 doublings), while BSCs in Beefy-9 continued to expand exponentially over at least seven passages (18.2 and 17.2 doublings for 40 ng/mL FGF-2 and 5 ng/mL FGF-2, respectively).

Converting growth data to doubling times revealed a consistent increase in doubling time over seven passages in Beefy-9 media, with a slower increase in doubling time (i.e., better-sustained proliferation) in BSC-GM (Fig. 5b); however, doubling times remained below 48 h for Beef-9 media for the first five passages (13.7 doublings) and under 56 h for all seven passages (18.2 doublings). The average doubling time over seven passages in Beefy-9 with high or low FGF-2 concentrations were ~39 and ~41 h, respectively. These are higher than the doubling time of ~17 h that has been reported for satellite cells in vivo, but within a range expected based on previous reports of BSCs cultured in vitro^22–24^. Together, these results indicated that the Beefy-9 media with both high and low FGF-2 concentrations were effective for the long-term expansion of bovine satellite cells, but that further optimization is needed to improve growth rates over multiple passages.

Phenotypic analysis of proliferating cells culture revealed the maintenance of BSC stemness over long-term culture in serum-free media. Specifically, >96% of cells in both BSC-GM and Beefy-9 stained positive for the early satellite cell marker Paired-box 7 (Pax7), and there was no significant difference in the Pax7+ population between media types (Fig. 5c). Similarly, qPCR and analysis of gene expression for the related gene Pax3 revealed no significant difference between media types (Figs. 5d and S5). Analyses of the myogenic commitment marker Myoblast Determination Protein 1 (MyoD) by immunostaining and qPCR revealed increased expression in passage two cells cultured in Beefy-9 compared with BSC-GM; however, this difference was absent by passage six (Figs. 5d and S5). Together, these results demonstrate that Beefy-9 maintains satellite cell identity to a similar degree as serum containing media.

At the same time, however, it was noted that cells cultured in serum-free conditions appeared to accumulate lipid droplets over long-term culture, while cells cultured in BSC-GM did not (Fig. 5e). This aberrant lipid accumulation could be due to insulin resistance in cells as a result of the relatively high concentration of insulin in B8 & Beefy-9, and could therefore point towards a possible media optimization strategy by adjusting insulin levels^25,26^. Alternately, lipid accumulation could suggest that BSCs are thrust towards an adipogenic phenotype in Beefy-9 media^11^, though the sustained myogenicity of BSCs in serum-free conditions affirms the capacity of these media to maintain relevant satellite cell function for cultured meat. At the same time, lipid accumulation could potentially offer an advantage for cultured meat production, as lipids often have positive impacts on meat flavor. Ultimately, further exploration of this phenomenon is warranted in future studies.

### BSC myogenicity over long-term culture

**Fig. 6:**
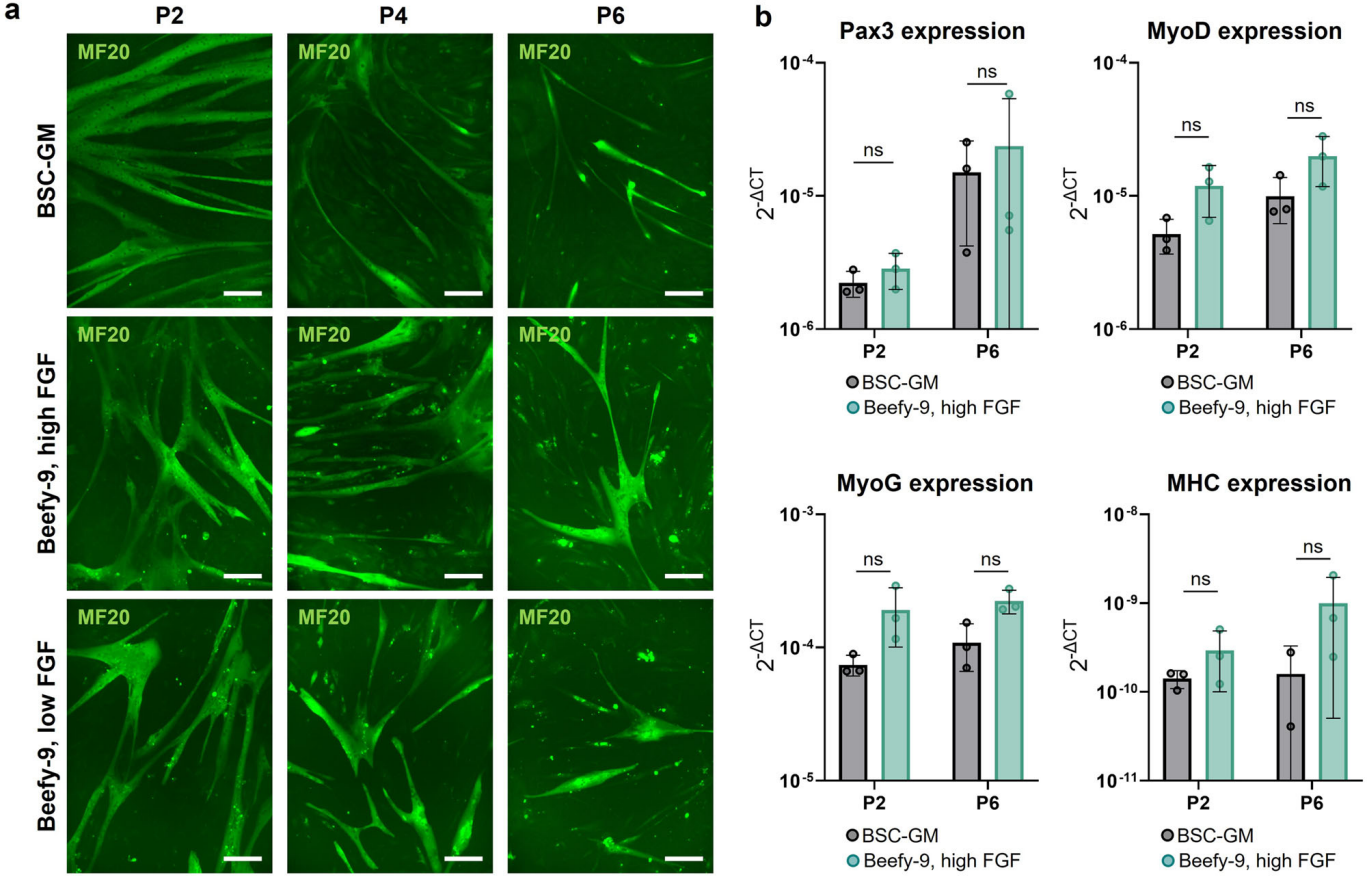
BSC Myogenicity over long term culture. **a** Immunofluorescent staining for Myosin Heavy Chain (MF20, green) in BSCs at passages 2, 4 and 6 in various media. Cells were cultured to confluency and differentiated in a serum-free differentiation medium as previously described. Images revealed that myogenicity of BSCs was maintained throughout long term culture in both serum containing and serum-free media. Scale bars are 200 μm. **b** Quantitative PCR for Pax3, MyoD, Myogenin (MyoG) and Myosin Heavy Chain (MHC) expression in differentiated cells shows no significant difference between media types. Results are supported by immunofluorescent staining for these markers (Fig. S6). *n* = 3 distinct samples; statistical significance was calculated via two-way ANOVA with multiple comparisons between media.

Following proliferation, confluent cells at various passages were differentiated in a serum-free differentiation medium^10^ and stained for Pax7 and MyoD as well as Myogenin and Myosin Heavy Chain (MHC), markers of early fusion and terminal differentiation in muscle cells (Figs. 6a and S6). The formation of Myogenin and MHC-positive multinucleated myotubes in BSC-GM and Beefy-9 formulations over six passages was shown, as was the maintenance of Pax7 and MyoD expression in unfused cells, which together support the maintained myogenicity of BSCs cultured long-term in Beefy-9 (Figs. 6a and S6). Interestingly, differentiation appeared improved in BSCs cultured in Beefy-9 media compared with BSC-GM over multiple passages, both in terms of myotube size and density, and in terms of quantitative fusion index (Figs. 6a, S6 and S7). This result could be because cells cultured in BSC-GM have undergone more doublings than cells in Beefy-9 at the points of analysis, and so represent a more aged satellite cell population^27^. While myogenicity was maintained throughout expansion in Beefy-9 media, it was noted that myotube diameter and density appears reduced at later passages. This is thought to be caused by cell aging, rather than fibroblast overgrowth, due to the maintenance of Pax7 at later passages and in unfused cells.

Along with image analysis, qPCR was used to analyze the expression of Pax3, MyoD, Myogenin, and MHC in differentiated cells (Fig. 6b). Results showed no significant differences between media types, though trends match those seen with immunostaining. Together, these results indicate that Beefy-9 maintains BSC myogenicity over long-term culture at least as well as serum-containing media.

### Media cost analysis

**Fig. 7:**
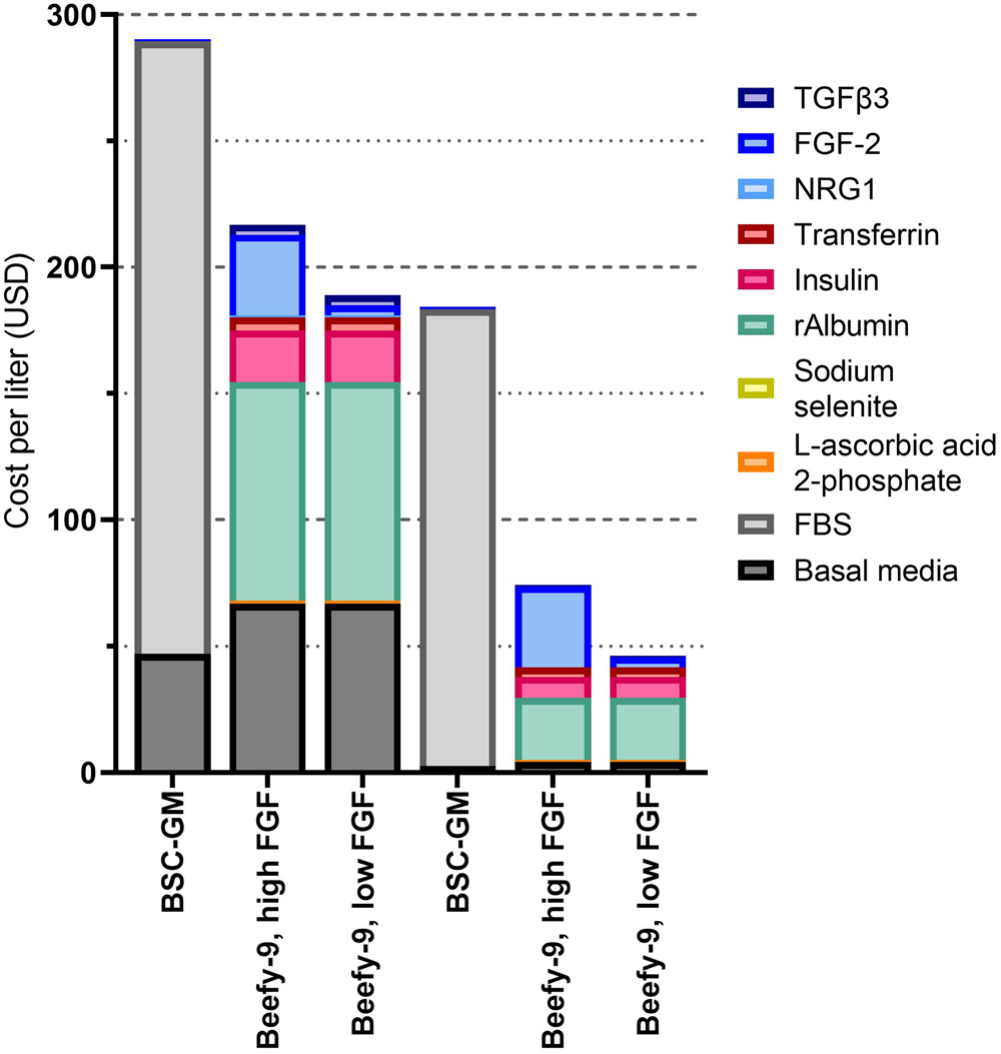
Cost analysis. Media costs for BSC-GM; Beefy-9, high FGF; and Beefy-9, low FGF were assessed for reagents bought at small scale (those used in this study), and reagents if bought in bulk from available suppliers. For both small-scale and bulk purchasing, Beefy-9 (high or low FGF) is more economical than BSC-GM. When purchased in bulk and using low FGF concentrations, Beefy-9 can be prepared for less than $50/L. For both small-scale and bulk purchasing, rAlbumin is a major cost-driver for Beefy-9 media, as is FGF-2 when used at 40 ng/mL.

Once the efficacy of Beefy-9 was demonstrated, a simple cost analysis was performed to understand how this media might be implemented by research groups currently relying on serum-containing media (e.g., 20% FBS + 1 ng/mL FGF-2, as used in this study) for cultured meat research (Fig. 7). Price comparisons revealed that even using purchased growth factors and without bulk ordering (as in this study), Beefy-9 media cost substantially less than serum-containing media ($217/L vs. $290/L, respectively). At the reduced 5 ng/mL of FGF-2, the price of Beefy-9 drops further to $189/L). Further fold price decreases could easily be achieved by increasing the scale of culture media component orders, and by using powdered basal media. Specifically, the costs for Beefy-9 with high or low FGF-2 concentrations dropped to $74/L and $46/L, respectively, when components are ordered in bulk (component sourcing given in Table S3). This amounts to a 75% cost reduction on a per-liter basis compared with bulk-ordered BSC-GM. However, it should be noted that over prolonged expansion, the cost-savings of Beefy-9 are mitigated by the exponential effects of reduced growth rates. Specifically, by passage 4 of prolonged expansion the cost-per-cell yielded is higher for Beefy-9 than for BSC-GM. As such, substantial cost optimization is required to make Beefy-9 cost-effective for large-scale cultured meat production. In this study, Beefy-9 prices were dominated by rAlbumin, basal media, FGF-2 (at high concentrations), and insulin. If bulk ordering of store-bought components is used, the price was dominated by FGF-2 (at high concentrations), rAlbumin, and insulin, with basal media offering less impact. While Beefy-9 is easy to produce in-house, further ease-of-use can be achieved by purchasing HiDef-B8 media and simply adding rAlbumin to prepare HiDef-Beefy-9; however, at current prices this results in increased cost.

### Increasing rAlbumin concentration improves growth

**Fig. 8:**
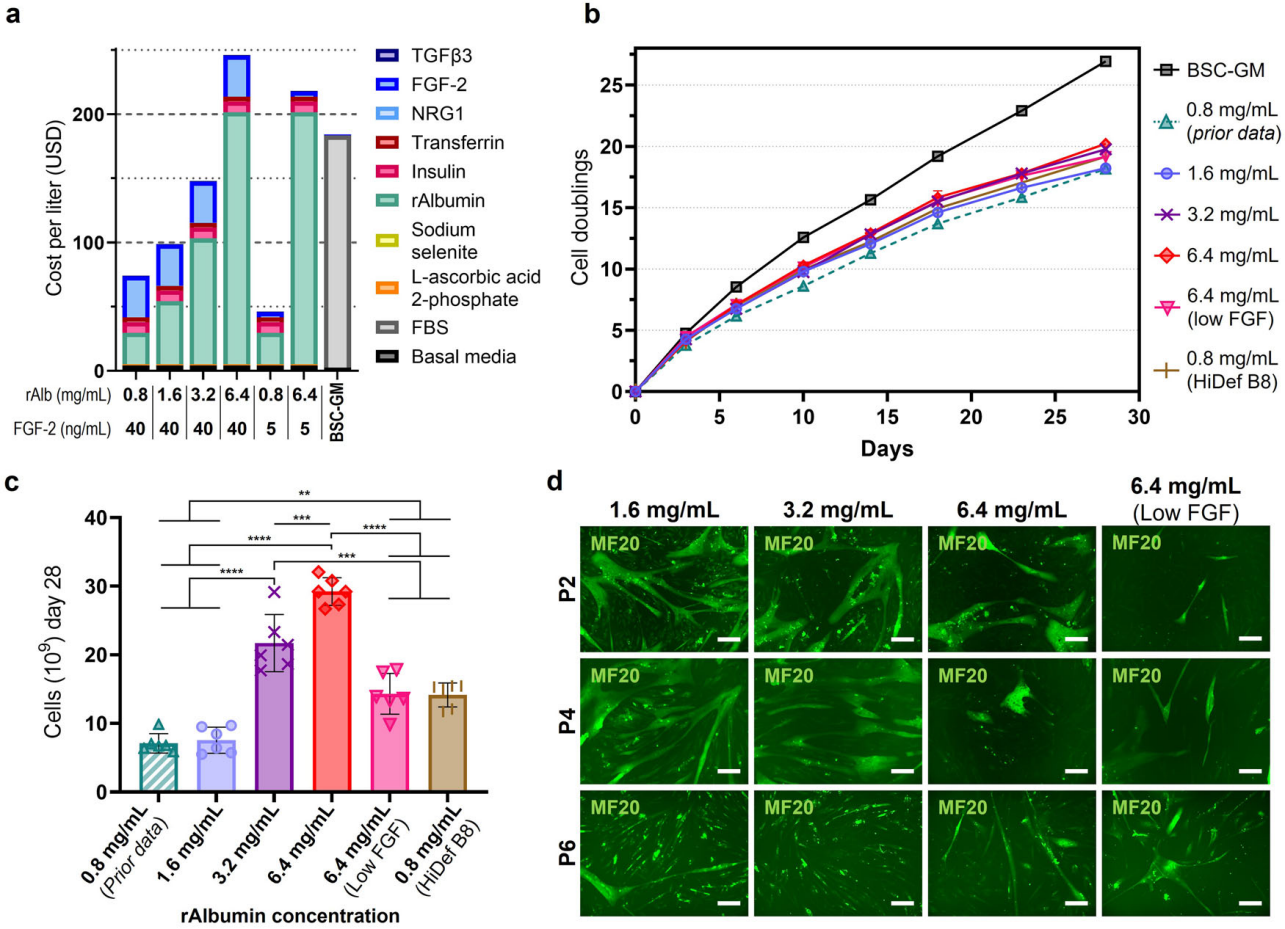
Long-term culture with increased rAlbumin. **a** Media costs for Beefy-9 with varying concentrations of rAlbumin and FGF-2, as well as BSC-GM. As before, costs in Beefy-9 are driven by rAlbumin. At and above 6.4 mg/mL, Beefy-9 costs surpass serum-containing BSC-GM costs. Costs represent bulk-orders from available suppliers. **b** Cell doublings over multiple passages of BSCs cultured in BSC-GM, Beefy-9 with rAlbumin concentrations of 0.8 mg/mL (data from Fig. 5), 1.6 mg/mL, 3.2 mg/mL, 6.4 mg/mL (with 40 or 5 ng/mL FGF-2), and HiDef Beefy-9 using supplier provided HiDef B8. Results showed that increased rAlbumin concentrations improved cell growth, with the highest numbers of cell doublings was provided by concentrations of 6.4 mg/mL (20.2) and 3.2 mg/mL (19.8) rAlbumin. *n* = 6 (two counts for three biological replicates), and error bars are given as ±standard deviation (though in some instances are smaller than the sample icons). **c** Final (28-day) cell counts for the serum-free media tested in **b** and with a starting cell population of 24,000. Results indicated significant increases in cell yield for 3.2 and 6.4 mg/mL rAlbumin compared with 0.8 mg/mL, as well as significant improvements when using engineered growth factors (in supplier provided HiDef B8). *n* = 6 (two counts for three biological replicates); statistical significance was calculated by one-way ANOVA with multiple comparisons between all conditions, and is indicated by asterisks, in which *p* < 0.05 (*), *p* < 0.01 (**), *p* < 0.001 (***), and *p* < 0.0001 (****). **d** Immunofluorescence staining for Myosin Heavy Chain (MF20, green) in BSCs at passages 2, 4 and 6 during long-term culture experiments in Beefy-9 with increased rAlbumin. After reaching confluency, cells were differentiated in serum-free differentiation medium for four days (P2 and P4) or six days (P6) and stained for MHC. Images revealed that the myogenicity of BSCs was maintained throughout long term culture in all media. Scale bars are 200 μm.

While 800 μg/mL of rAlbumin was used in the above work, short term growth analysis did not suggest that this was the optimal concentration of rAlbumin, merely the best of those initially tested. As increased rAlbumin resulted in improved growth (Fig. 2), it was investigated whether additional increases in rAlbumin could further improve outcomes. Short term analyses with concentrations of rAlbumin up to 11.2 mg/mL revealed continued improvements with increased rAlbumin, with the highest concentration yielding an 8.5-fold improvement in 4-day growth compared to Beefy-9’s 800 ug/mL (0.8 mg/mL) (Fig. S8). In light of this, long-term growth was again investigated with increasing concentrtions of rAlbumin, and compared with the original Beefy-9 tests, HiDef Beefy-9 (using supplier-provided B8 with engineered growth factors), and BSC-GM (Fig. 8). While short-term growth analyses revealed that 11.2 mg/mL of rAlbumin offered the best results of the concentrations tested, cost analysis revealed that by 6.4 mg/mL rAlbumin, Beefy-9 had already surpassed the cost of serum-containing BSC-GM (Fig. 8a). Because cost was a key factor in this study, long term growth analysis was therefore performed for rAlbumin concentrations up to, but not exceeding 6.4 mg/mL (Fig. 8b). Results showed that increased albumin improved growth over one month of cell expansion, with total doublings exceeding 19 and 20 for Beefy-9 containing 3.2 and 6.4 mg/mL rAlbumin, respectivley (compared with 18 for the original Beefy-9 containing 0.8 mg/mL rAlbumin). Taken as total cell count at day 28, these represent 3- and 4-fold improvements in cell yield, respectively (Fig. 8c). No improvement was observed for 1.6 mg/mL rAlbumin compared with 0.8 mg/mL at day 28, though a consistent improvement was seen for earlier passages. When comparing growth improvements with costs, increasing rAlbumin to 3.2 mg/mL resulted in a 3-fold improvement in cell number with only a 2-fold increase in cost. For 6.4 mg/mL rAlbumin, these values are 4-fold and 3-fold, respectively. As such, Beefy-9 with 3.2 mg/mL of rAlbumin (termed Beefy-9+) may offer the best ratio of cell growth to media cost (148 $/L). However, the best media for a given scenario will likely depend on the specific constraints, priorities, and target doublings of the application in question, and all of the serum-free media tested were inferior to BSC-GM, which showed a 100-fold increase in cell yield over seven passages compared with serum-free media. Throughout culture in Beefy-9 with additional rAlbumin, cells maintained their myogenicity (Fig. 8d), though the degree of MF20 staining as well as myotube density and diameter appear to have been reduced for cells grown in media containing 6.4 mg/mL rAlbumin (especially with reduced FGF). While Beefy-9+ with 3.2 mg/mL rAlbumin and reduced FGF was not explored in this work, it is possible that this would further improve the performance/cost ratio. Additionally, the use of engineered thermally-stable growth factors (as in the original B8 work, and in supplier-provided HiDef B8) may improve performance. This was evidenced in long-term growth studies, where a 2-fold improvement in cell number was seen between HiDef Beefy-9 compared with standard Beefy-9.

## Discussion

Since its emergence, cultured meat research and development has been stymied by a lack of suitable serum-free media for muscle stem cell expansion. This deficiency has led the field to rely on FBS for most research, thereby hindering the relevance of findings. This is particularly the case when looking forward to the serum-free processes which will be necessary for scalable and sustainable cultured meat production. Developing serum-free media for relevant cell types (e.g., muscle and fat) and relevant species (e.g., bovine, porcine, galline, etc.) is therefore essential to accelerate research in the field. These media should at a minimum be affordable, comprised of food-safe components, reliable, and easy to use (e.g., not overly complex) in order to promote their adoption in widespread research efforts and towards scaled production processes. In this work, two media (Beefy-9 and Beefy-9+) are described as promising candidates for simple serum-free culture of bovine satellite cells. This includes validation of: (1) the efficacy of these media in promoting BSC proliferation, (2) the ability of these media to enable long-term expansion when used in combination with rAlbumin-free B8 and truncated vitronectin, (3) the maintenance of satellite cell myogenicity when expanded in Beefy-9 and Beefy-9+, and (4) cost savings compared to serum-containing media.

The impact of rAlbumin on Beefy-9’s efficacy for BSCs is noteworthy, particularly considering that the addition of albumin did not show nearly as profound of an effect on iPSC growth when B8 was originally developed^14^. Albumin has many roles in cell culture media, and indeed comprises about 60% of the total proteins in serum. For an in-depth summary of these roles, the reader is directed to several thorough reviews^28,29^. Briefly, though, albumin acts in culture media to bind, carry, and stabilize compounds such as fatty acids, metal ions, signaling molecules, amino acids, and other factors. As such, it is a potent and multifaceted antioxidant which can sequester these species from redox or other degrading reactions, increasing the half-life, availability, and solubility of beneficial factors while reducing the accumulation of harmful byproducts. It is possible that these regulating effects impart an advantage to Beefy-9 and Beefy-9+ over B8 alone, as the latter was not incapable of promoting cell division in BSCs over the short term, only of fostering robust and prolonged expansion. Interestingly, albumin has also been suggested as a potential protectant against cell stresses due to sparging bubbles in various bioreactors, and so Beefy-9 media could offer additional benefits in these scaled-up bioprocesses^29^.

A major goal of this work was to develop a medium that is simple and low-cost, as these two factors can help to lower the barriers to entry for cultured meat research, as well as lay the groundwork for a more diffuse production system (with less consolidation amongst a few concentrated corporations). Cost analysis suggests that Beefy-9 and Beefy-9+ media should be easy to implement and affordable (particularly in the case of Beefy-9) for academic labs that are currently using serum-containing (e.g., 20% FBS) media for cultured meat research. It should be noted, however, that substantial cost reduction of serum-free media is still needed to make cultured meat production economically viable from an industrial perspective. Here, reducing the cost of rAlbumin, growth factors and basal media (e.g., through the use of plant or algal hydrolysates) will be essential^30,31^. Additionally, co-culture of meat-relevant cells with nutrient- or growth factor-producing cells could offer valuable cost-saving opportunities^32^. Finally, media production in-house and economies of scale will likely further drive down costs^14^. When considering Beefy-9, it is clear that recombinant proteins are the main drivers of cost. As such, further research should explore cost-reduction of these recombinant proteins, or opportunities for substituting or eliminating these factors. Additionally, it should be noted that in the absence of cell-adhesion proteins found in FBS, an increased concentration of tissue-vessel coating was required to achieve adequate BSC adhesion and growth during serum-free culture. This factor is often overlooked when discussing the cost of large-scale cell culture; however, the present study relied on 1.5 μg/cm^2^ of Vtn-N, which adds ~$0.18/cm^2^, or $31.75 per T-175 flask (a standard vessel used in our lab, suitable for ~5 doublings at standard seeding and passaging densities). Opportunities to reduce the costs associated with cell adhesion include adapting or engineering cells to suspension culture, pre-coating flasks with Vtn-N with or without additional factors, reducing the cost of recombinant adhesion protein production, or exploring low-cost alternatives to recombinant production^4,33,34^.

While this work presents a promising resource for researchers and a useful foundation for continued media development, it is clear that further media optimization for both cost and long-term efficacy is warranted. Specifically, the present work relies on one-factor-at-a-time exploration of media components to find suitable supplements to tailor B8 for bovine muscle stem cells. As media components are intimately entangled in their effects on cell biology, it is unlikely that Beefy-9 or Beefy-9+ are optimal as-is. Indeed, it is possible that media components which appeared inconsequential in this work (e.g., hepatocyte growth factor or ethanolamine) would offer advantages in other permutations of media not tested in the development of Beefy-9, or even that other forms of these factors with improved bioactivity or stability could effectively improve Beefy-9. Computational approaches to media development are better suited for solving such multi-factorial problems, and should therefore be leveraged to further optimize Beefy-9 and other serum-free media for cultured meat applications^31^. Here, efforts can focus on adipose as well as muscle tissue, for instance through the use of free fatty acid addition to Beefy-9 to induce the transdifferentiation of BSCs into lipid-accumulating cells^35^. Additionally, further work is needed to overcome the slower long-term growth and aberrant lipid accumulation that is seen in BSCs cultured over seven passages in Beefy-9. Particularly, efforts to reduce doubling times to those seen in the presence of serum, or even in vivo, would improve the overall process economics of cultured meat production. Promising options for this include further media optimization, the use of spontaneously or genetically immortalized stem cells, which could improve long-term outcomes in Beefy-9^36,37^, and the exploration of different concentrations of insulin, fatty acids, or other signaling factors to better control progression down myogenic vs. adipogenic pathways. Here, the use of other serum-free differentiation media may also improve outcomes, and should be explored^38^.

The present work offers a simple, affordable, and effective Beefy-9 and Beefy-9+ serum-free media for improving cultured meat research. However, the cost and efficacy of these media will require further optimization for industrial scale production of cultured meats that aim to reach price-parity with conventional meats. Future work to address these needs could focus on both engineering efforts (e.g., increased recombinant growth factor productivity, production of species-specific recombinant proteins, optimized media formulations, media recycling, and cell line engineering) and scientific discoveries (e.g., novel protein analogues or alternatives, or insights into cell signaling pathways) in order to drive the cost of production down. Additionally, exploration of cell sourcing and isolation techniques, cell lineage control (e.g., of myogenic vs. adipogenic differentiation), and bioreactor systems can build on substantial prior work and help to increase production efficiency and drive down costs^13,39–42^. Here, cultured meat development is likely to provide collateral benefit to biomedical research, such as tissue engineering for volumetric muscle loss or cell-based biopharmaceutical production. Ultimately, sustained efforts towards serum-free media development are needed to continually lower costs and improve scalability of cultured meat over time, bringing products closer to market viability and cultured meat’s possible benefits closer to reality.

## Materials and methods

### Primary bovine satellite cell isolation and maintenance

Primary bovine satellite cells (BSCs) were isolated with methods previously used in our group and based on previously described pre-plating satellite-cell isolation protocols^6,41^. Briefly, ~0.5 g of muscle was excised from the semitendinosus of a 14-day-old Simmental calf at the Tufts Cummings School of Veterinary Medicine according to approved methods (IACUC protocol #G2018-36). Muscle tissue was minced into a paste and digested in 0.2% collagenase II (Worthington Biochemical #LS004176, Lakewood, NJ, USA; 275 U/mg) for 45 min with regular trituration. Digestion was halted with BSC growth media (BSC-GM) comprised of DMEM + Glutamax (ThermoFisher #10566024, Waltham, MA, USA) supplemented with 20% fetal bovine serum (FBS; ThermoFisher #26140079), 1 ng/mL human FGF-2 (ThermoFisher #68-8785-63), and 1% Primocin (Invivogen #ant-pm-1, San Diego, CA, USA), and cells were filtered and plated at a density of 100,000 cells/cm^2^ onto uncoated tissue-culture flasks. After 24 h of incubation at 37 °C with 5% CO_2_, the plated suspensions (containing satellite cells) were transferred to flasks coated with 1 μg/cm^2^ mouse laminin (Sigma #CC095, St. Louis, MO, USA), which were left untouched for three days before growth media was changed, and cells were cultured using standard practices on tissue-culture plastic coated with 0.25 ug/cm^2^ iMatrix recombinant laminin-511 (Iwai North America #N892021, San Carlos, CA, USA). After two weeks of culture, Primocin in growth media was replaced with 1% antibiotic-antimycotic (ThermoFisher #1540062). For regular cell maintenance, cells were cultured at 37 °C in 5% CO_2_ to a maximum of 70% confluence, counted using an NC-200 automated cell counter (Chemometec, Allerod, Denmark), and either passaged using 0.25% trypsin-EDTA (ThermoFisher #25200056) or frozen in FBS with 10% Dimethyl sulfoxide (DMSO, Sigma #D2650). For routine myogenic differentiation, cells were cultured to confluency as above and then incubated for one week without changing the medium.

### Characterization of isolated cells

To characterize isolated cells, proliferative BSCs were stained for Paired-box 7 (Pax7), a marker of satellite cell identity. Cells were fixed with 4% paraformaldehyde (ThermoFisher #AAJ61899AK) for 30 min, washed in PBS, permeabilized for 15 min using 0.5% Triton-X (Sigma # T8787) in PBS, blocked for 45 min using 5% goat serum (ThermoFisher #16210064) in PBS with 0.05% sodium azide (Sigma #S2002), and washed with PBS containing 0.1% Tween-20 (Sigma #P1379). Primary Pax7 antibodies (ThermoFisher #PA5-68506) were diluted 1:500 in blocking solution containing 1:100 Phalloidin 594 (ThermoFisher #A12381), added to cells, and incubated overnight at 4 °C. Cells were then washed with PBS + Tween-20, incubated with secondary antibodies for Pax7 (ThermoFisher #A-11008, 1:500) for 1 h at room temperature, washed with PBS + tween-20, and mounted with Fluoroshield mounting medium with DAPI (Abcam #ab104139, Cambridge, UK) before imaging. Imaging was performed via fluorescence microscopy (KEYENCE, BZ-X700, Osaka, Japan). Batch colocalization analysis was performed on multiple images using the BZ-X800 image cytometry software to enumerate the nuclei which were positive for Pax7, thereby giving a quantitative measure of satellite cell purity in the isolated cell population.

To validate myogenicity of isolated BSCs, cells were differentiated for 7 days as described. Cells were then fixed, stained, and imaged as previously described, using primary antibodies for myosin heavy chain (MHC) (Developmental studies hybridoma bank #MF-20, Iowa City, IA, USA; 4 μg/mL), phalloidin 594 (1:100), appropriate secondary antibodies (ThermoFisher #A-11001, 1:1000), and Fluoroshield mounting medium with DAPI.

### Short-term growth analysis

Homemade B8 medium was prepared using store-bought components and a previously described formulation and method of preparation (Table S1)^14^. Additionally, HiDef-B8 medium aliquots were generously provided by Defined Bioscience (Defined Bioscience # LSS-201, San Diego, CA, USA) and added to DMEM/F12 with 1% antibiotic/antimycotic. Short term BSC growth (3 and 4 days) was analyzed for mixtures of serum-containing and serum-free media, as well as for pure B8 media with reduced growth factor concentrations and/or with the addition of various media supplements (Table S1). Briefly, BSCs were thawed (passage number < 2) and plated in BSC-GM on 96-well tissue-culture plastic plates for each timepoint at a density of 2,500 cells/cm^2^ with 0.25 ug/cm^2^ iMatrix recombinant laminin-511. After 24 h, BSC-GM was removed, cells were washed 1x with DPBS, and new media (e.g., B8± supplementation) was added. A list of supplements and concentrations can be found in Table S1. Media was changed on day 3, and on days 3 and 4 cells were imaged, media was aspirated from appropriate plates, and plates were frozen at −80 °C. Once all timepoints were frozen, cell number was analyzed using a FluoReporter dsDNA quantitation kit (ThermoFisher #F2962) according to the recommended protocol and with fluorescence readings performed on a Synergy H1 microplate reader (BioTek Instruments, Winooski, VT, USA) using excitation and emission filters centered at 360 and 490 nm, respectively. Cell number at 3 and 4 days was analyzed relative to pure B8 or HiDef media.

### Passaging in Beefy-9 media

To test various passaging conditions using B8 + rAlbumin (Beefy-9), BSCs were plated in BSC-GM onto T-75 culture flasks at a density of 2,500 cells/cm^2^ with 0.25 ug/cm^2^ iMatrix recombinant laminin-511. After 24 h, BSC-GM was removed, cells were washed 1x with DPBS, and Beefy-9 media was added. Cells were cultured to 70% confluency, harvested with TrypLE Express (ThermoFisher #12604021), centrifuged at 300 g, and resuspended in B8 or Beefy-9 media with or without iMatrix laminin-511. Cells were seeded at 5,000 cells/cm^2^ (0.25 ug/cm^2^ iMatrix laminin) onto a 12-well plate, and growth was analyzed with a live cell monitoring system (Olympus Provi CM20, Tokyo, Japan). After 24 h, media was aspirated, and all cells were fed with Beefy-9 media. Cell growth was compared over seven days in order to determine the effects of seeding ±rAlbumin and ±iMatrix laminin.

To test the effect of different coatings, 48-well plates were prepared with or without pre-coating with poly-D-lysine (Sigma #P1024-10MG) according to the manufacturer’s instructions. Cells were then cultured and harvested as above, centrifuged at 300 g, and resuspended in B8 media with varying concentrations if iMatrix laminin and/or truncated recombinant human vitronectin (ThermoFisher #A14700). Cells were seeded at 5,000 cells/cm^2^. After 24 h, media was aspirated, and cells were rinsed 1x with DPBS. Cells were then fed with Beefy-9 media with 10% PrestoBlue reagent (ThermoFisher #A13262) and incubated at 37 °C. After 2.5 h, PrestoBlue media was moved to a 96-well plate and read with a Synergy H1 microplate reader using excitation and emission filters centered at 560 and 590 nm, respectively. Cell culture media was then replenished with Beefy-9 and PrestoBlue analysis was repeated on Day 4.

### Long-term growth analysis

For generating long-term growth curves, BSCs were thawed and plated (P1) onto 6-well culture plates (triplicate wells) in BSC-GM with 0.25 ug/cm^2^ iMatrix laminin-511. After allowing cells to adhere overnight, media was removed, cells were washed 1x with DPBS, and either BSC-GM, B8, Beefy-9 (40 ng/mL FGF-2) or Beefy-9 (5 ng/mL FGF-2) were added to cells. Upon reaching ~70% confluency, cells were rinsed with DPBS, harvested with TrypLE Express, and counted using an NC-200 automated cell counter (duplicate counts for each well). Cells were then pelleted at 300 g, resuspended in BSC-GM or B8 media, re-counted, and seeded onto new 6-well plates at 2,500 cells/cm^2^ with 0.25 ug/cm^2^ iMatrix laminin-511 (cells in BSC-GM) or 1.5 ug/cm^2^ recombinant vitronectin (cells in B8). After allowing cells to adhere overnight, media was replaced with appropriate media (BSC-GM, B8, Beefy-9 (40 ng/mL FGF-2) or Beefy-9 (5 ng/mL FGF-2). This process was repeated over 28 days and seven passages. Throughout culture, cells were fed every two days. When seeding cells for passages 2, 4, 6, and 7, additional wells were seeded for staining for myosin heavy chain (P2, P4 and P6) or lipid accumulation (P7). This method was used as well for long-term growth analysis of Beefy-9 with increased rAlbumin concentrations, with the only difference being the media in question.

### Serum-free differentiation

Throughout long-term culture, a population of cells at passages 2, 4, and 6 were cultured to confluency in serum-containing or serum-free conditions, and media was changed to a previously described serum-free differentiation media consisting of Neurobasal (Invitrogen #21103049, Carlsbad, CA, USA) and L15 (Invitrogen #11415064) basal media (1:1) supplemented with 1% antibiotic/antimycotic, 10 ng/mL insulin-like growth factor 1 (IGF-1; Shenandoah Biotechnology #100-34AF-100UG, Warminster, PA, USA) and 100 ng/mL epidermal growth factor (EGF; Shenandoah Biotechnology #100-26-500UG)^10^. Cells were differentiated for 4-6 days (changing media every two days).

### Immunostaining of cultured & differentiated cells

To verify the identity of sub-confluent cells over multiple passages in various media, cells were fixed, stained, and imaged as previously described, using primary antibodies for Pax7 and MyoD (ThermoFisher #MA5-12902; 1:100) along with DAPI and secondary antibodies for rabbit (ThermoFisher #A-11072; 1:500) and mouse (ThermoFisher #A-11001), respectively. Pax7 and MyoD expression were quantitatively analyzed in ImageJ. To verify the ability of cells to differentiate over multiple passages in various media, differentiated cells from above were fixed, stained, and imaged as previously described, using primary antibodies for Pax7, MyoD, Myogenin (Santa Cruz Biotecnology #sc-52903, Dallas, TX, USA; 1:250), MHC, DAPI, and appropriate secondary antibodies.

To analyze lipid accumulation in culture, media was replaced with PBS containing 2 μM BODIPY™ 493/503 (ThermoFisher #D3922). Cells were incubated for 20 min at 37 °C, washed 2x with PBS, and fixed for 30 min with 4% paraformaldehyde. Cells were then washed 3x with PBS, permeabilized and blocked as before, and incubated at room temperature for one hour in blocking solution containing phalloidin 594 (1:100) and 1 μg/mL DAPI (ThermoFisher #62248). Stained cells were rinsed 3x with PBS and imaged via fluorescence microscopy as before.

### Gene expression analysis

To further support immunostaining data, quantitative PCR was performed on sub-confluent and differentiated cells from P2 and P6. Briefly, RNA was harvested with the RNEasy Mini Kit (Qiagen #74104, Hilden, Germany) and cDNA was prepared using 200 ng of RNA for each reaction with the iScript cDNA synthesis kit (Bio-Rad # 1708890, Hercules, CA, USA) according to the manufacturers’ instructions. Next, qPCR was performed using the TaqMan Gene Expression Master Mix (ThermoFisher #4369016), with 18 S as a housekeeping control gene. Primers used were: 18 S (ThermoFisher #Hs03003631), Pax3 (ThermoFisher #Bt04303789), MyoD (ThermoFisher #Bt04282788), Myogenin (ThermoFisher #Bt03258929), and Myosin Heavy Chain (ThermoFisher #Bt03273061). Reactions were performed using 3.5 uL of the prepared cDNA, according to the manufacturer’s instructions. Reactions were run on the Bio-Rad CFX96 Real Time System thermocycler, and results were analyzed as 2^−Δct^ normalized to 18 S expression.

### Statistical analysis

Statistical analysis was performed with GraphPad Prism 9.0 software (San Diego, CA, USA). Short-term cell growth analyses were performed via one-way or two-way ANOVA, as appropriate, with multiple comparisons performed with the Tukey’s HSD post-hoc test. Regression analysis (Fig. 3a) was performed via nonlinear regression (least squares; exponential (Malthusian) growth) and is shown alongside 95% confidence interval. Doubling time (Fig. 5b) was determined through nonlinear regression (least squares; exponential (Malthusian) growth) for each biological replicate (well) of each passage, using technical replicates (duplicate counts) in generating nonlinear regressions. *P* values <0.05 were treated as significant. Unless otherwise stated, errors are given as ± standard deviation.

### Reporting summary

Further information on research design is available in the Nature Research Reporting Summary linked to this article.

## Supplementary information

Supplementary Information

Description of Additional Supplementary Files

Supplementary Video 1

Supplementary Data 1

Reporting Summary

## Data Availability

The authors declare that the data supporting this study are available within the article’s Supplementary files. Extra data are available from the corresponding author upon request.
