# Supplementary material for: Simple and effective serum-free medium for sustained expansion of bovine satellite cells for cell cultured meat
Source: Commun Biol. 2022 Jun 2;5:466. doi: 10.1038/s42003-022-03423-8 (PMC9163123; doi:10.1038/s42003-022-03423-8)
Supplement: Supplementary file 1 — Supplementary Information [file 42003_2022_3423_MOESM1_ESM.pdf]

Simple and effective serum-free medium for sustained expansion of bovine satellite cells for cell cultured meat - **Supplementary Materials**

Andrew J. Stout, Addison B. Mirliani, Miriam L. Rittenberg, Michelle Shub, Eugene C. White, John S.K. Yuen Jr., David L. Kaplan

Supplementary Figure 1: Bovine Satellite Cell (BSC) characterization and myogenicity

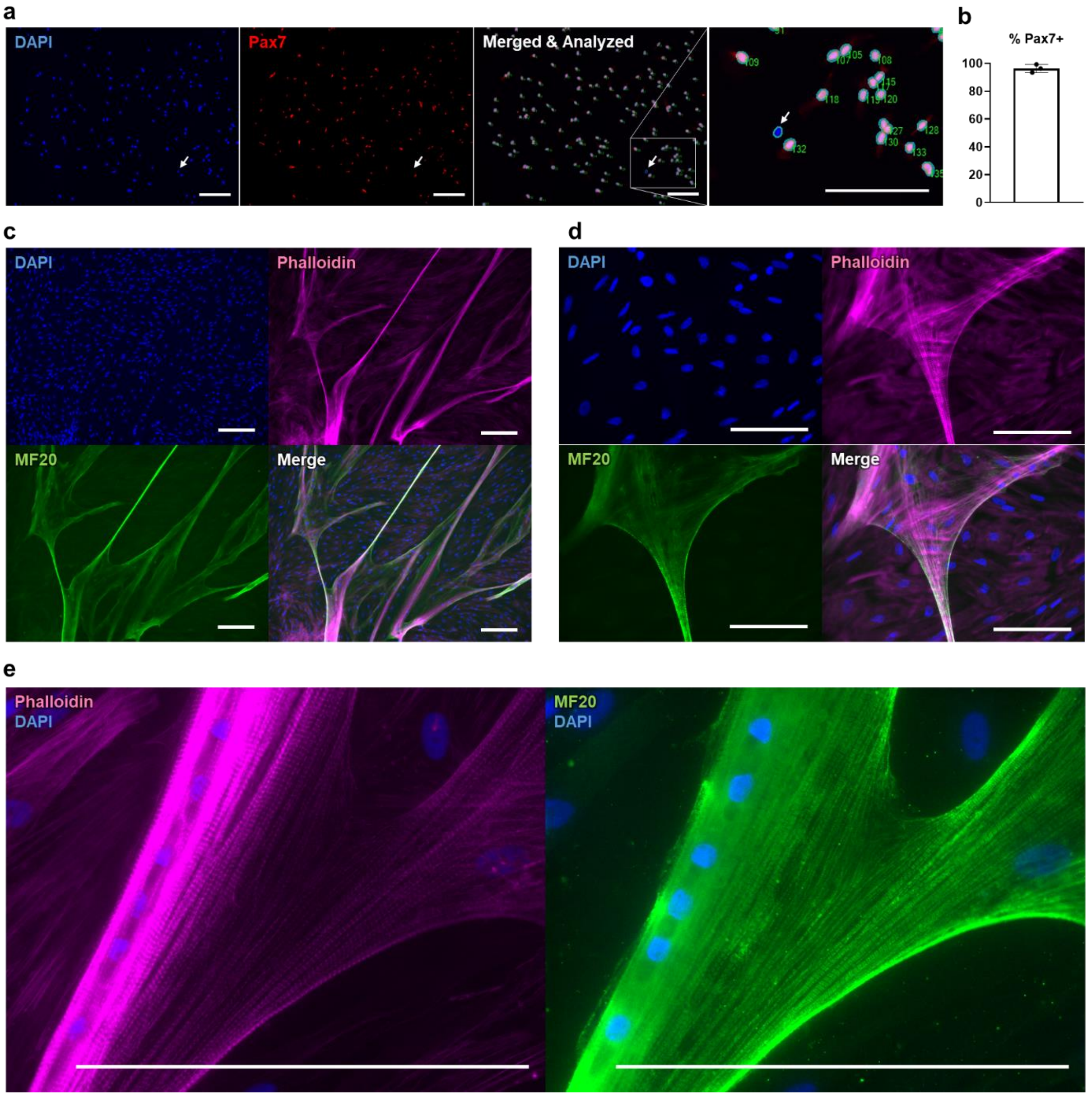

Figure S1: Bovine Satellite Characterization. (a) Isolated BSCs were stained and imaged for Pax7, the primary muscle stem cell marker, and DAPI (nuclei). Images were analyzed for colocalization of red (Pax7) and blue (DAPI) using the KEYENCE BZ-X800 analysis software to determine the number of nuclei that were positive for Pax7. Images show a representative analysis for one stain, with a Pax7-negative cell indicated with a white arrow. The rightmost image shows a magnified portion of the merged and analyzed image. Scale bars are 100 µm. **(b)** Quantitative Pax7 analysis from image cytometry described in (a). Average percent of Pax7+ cells was 96.32% (n=3) for isolated BSCs. **(c-e)** Myogenic differentiation of isolated BSCs was determined through staining for myosin heavy chain (MF20), and counterstaining with DAPI and Phalloidin (actin). Images show robust myogenic differentiation. At high magnification, actin & myosin striations are visible. Scale bars are 200 µm.

Supplementary Table 1: B8 formulation and sourcing for this study.

Component	Concentration	Supplier	Catalog #
**DMEM/F12** basal media	N/A	ThermoFisher	11320033
2-Phospho-**L-ascorbic acid** trisodium salt	200 µg/mL	Sigma	49752-10G
**Insulin** (human, recombinant)	20 µg/mL	Sigma	91077C-250MG
**Transferrin** (human, recombinant)	20 µg/mL	InVitria	777TRF029
Sodium **selenite**	20 ng/mL	Sigma	S5261-10G
Fibroblast growth factor (**FGF-2**)	40 ng/mL	PeproTech	100-18B
Neuregulin (**NRG1**)	0.1 ng/mL	PeproTech	100-03
Transforming growth factor (**TGFβ3**)	0.1 ng/mL	R&D Systems	8420-B3-005/CF
UltraPure **Water**	5.8% (v/v)	ThermoFisher	10977015
**Antibiotic/Antimycotic**	1% (v/v)	ThermoFisher	1540062

Functional species in media components are bolded. To prepare media, two aliquots were first prepared. For aliquot 'A,' 400 mg/mL 2-Phospho-L-ascorbic acid trisodium salt was slowly prepared in water, sterilized, and aliquoted in 250 µL portions. For aliquot 'B,' 40 mg/mL insulin was added to water, and 1 N HCl was added until insulin had dissolved. Slowly, 1 N NaOH was added to bring the pH up to ~6. Next, 40 mg/mL transferrin, 40 µg/mL sodium selenite, 80 µg/mL FGF-2, 0.2 µg/mL NRG1, and 0.2µg/mL TGFβ3 were added and the solution was sterilized and aliquoted in 250 µL portions. To prepare media, DMEM/F12 (500 mL) was sterilized with 5.3 mL of 100x Antibiotic/Antimycotic and 31 mL of water through a sterile filter, and aliquots A and B were added after sterilization. Following protocol previously described for B8 media, media used in this study was used within one month of preparing from frozen aliquots, and media was warmed to room temperature before feeding cells.

Supplementary Table 2: Summary of all media supplements tested.

Component	Supplier	Catalogue #	Range (ng/mL)
Hydrocortisone	Sigma	H0888-1G	3.125—100
Estradiol	Sigma	E8875-1G	1—5,000
Progesterone	Sigma	P8783-1G	0.3125—10
Dexamethasone	Sigma	D4902-100MG	3.125—400
BMS 564929	Tocris Bioscience	5274	0.1—500
Bovine growth hormone	MP Biomedical	02160074.1	7.8125—250
Curcumin	Sigma	C7727-500MG	1—5,000
Spermidine	Sigma	S2626-1G	100—500,000
Ethanolamine	Acros Organics	149582500	10—50,000
Linoleic acid	Sigma	L5900-10MG	25—3,200
Oleic acid	Sigma	O1257-10MG	25—3,200
Stat3 inhibitor	Sigma	573096-1MG	100—500,000
Interleukin-6	BioRad	PBP021	0.01—50
Leukemia inhibitory factor	Peprotech	300-05	0.3125—40
Hepatocyte growth factor	Novus Biologicals	NBP2517300.1MG	0.3125—40
Platelet derived growth factor BB	ThermoFisher	PHG0045	0.15625—20
Pigment-epithelium derived factor	R&D Systems	1177SF025	0.390625—50
Insulin-like growth factor	ThermoFisher	PHG0078	0.1—50
Cardiotrophin 1	Novus Biologicals	NBP199745	0.15625—20
Recombinant human albumin	Sigma	A9731-1G	100,000—800,000

Supplementary Figure 2: BSC growth in dilutions of BSC-GM and HiDef-B8

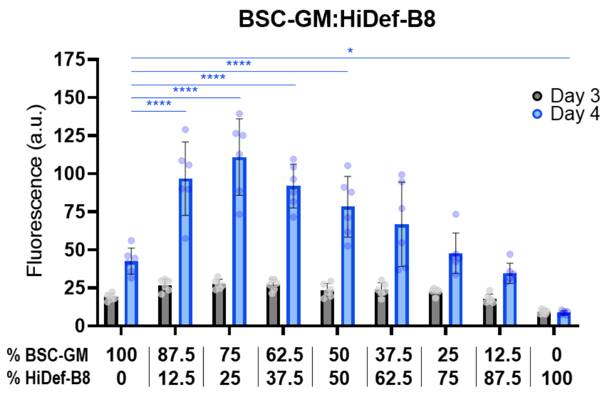

Figure S2: Short-term growth in BSC-GM mixed with HiDef-B8. BSC proliferation over 3 & 4 days in mixtures of BSC-GM (20% FBS) HiDef-B8 media. Results show that up to 50% HiDef-B8 significantly improves growth compared to BSC-GM, and that up to 87.5% does not significantly reduce growth compared to BSC-GM. n=6 distinct samples; statistical significance was calculated by one-way ANOVA on day 4 data comparing all samples with BSC-GM controls, and is indicated by asterisks, in which $p < 0.05$ (*), $p < 0.01$ (**), $p < 0.001$ (***), and $p < 0.0001$ (****).

Supplementary Figure 3: Individual supplement effects on B8 media

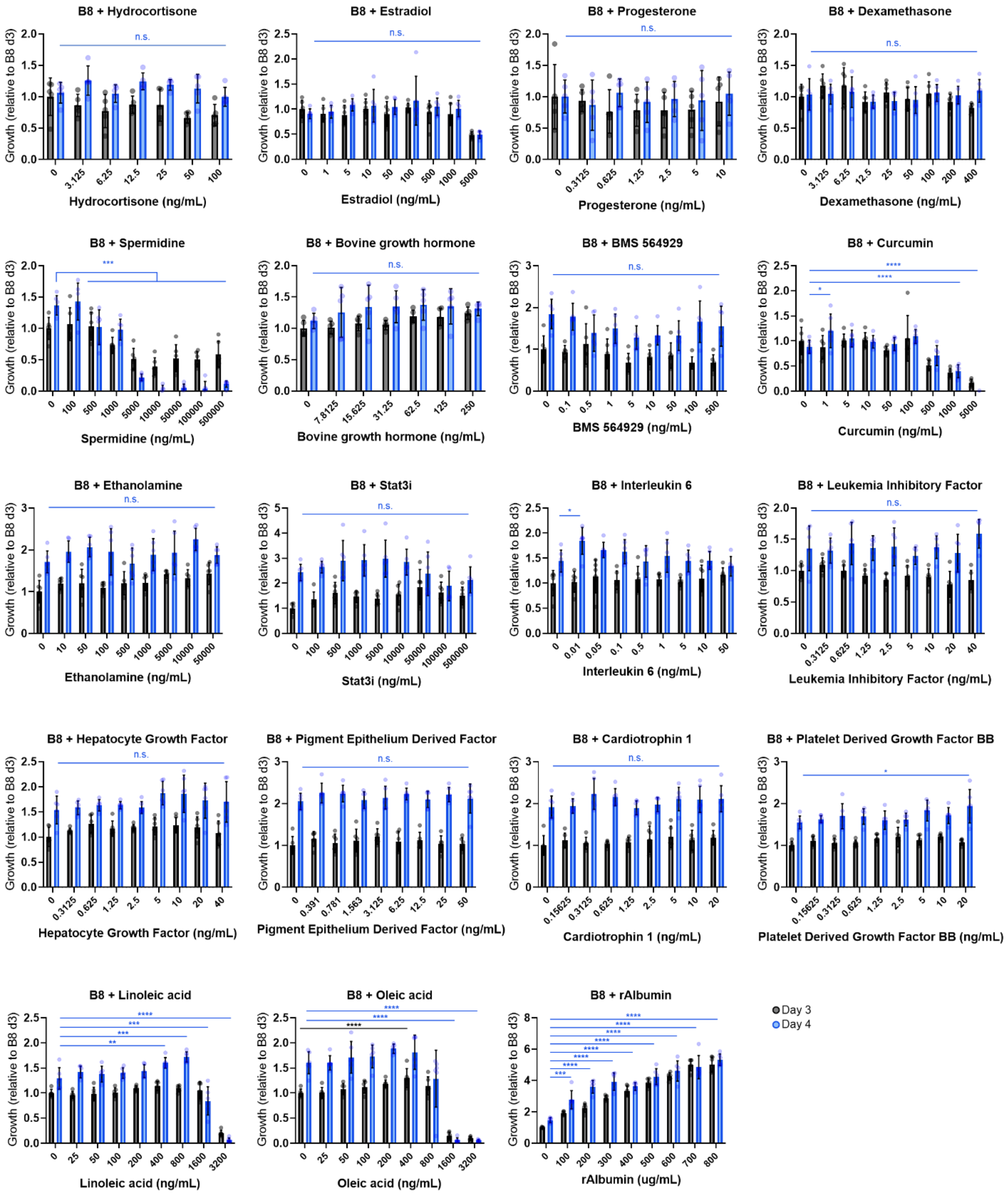

Figure S3: Short-term growth in supplemented B8 media. BSC proliferation over 3 & 4 days B8 supplemented with various supplements. Values are fluorescence based and given relative to day 3 B8 values. n=6 distinct samples; statistical significance was calculated by one-way ANOVA on day 4 data with multiple comparisons between supplemented samples and B8 controls, and is indicated by asterisks, in which $p < 0.05$ (*), $p < 0.01$ (**), $p < 0.001$ (***), and $p < 0.0001$ (****).

Supplementary Figure 4: Combined supplement effects on B8 media

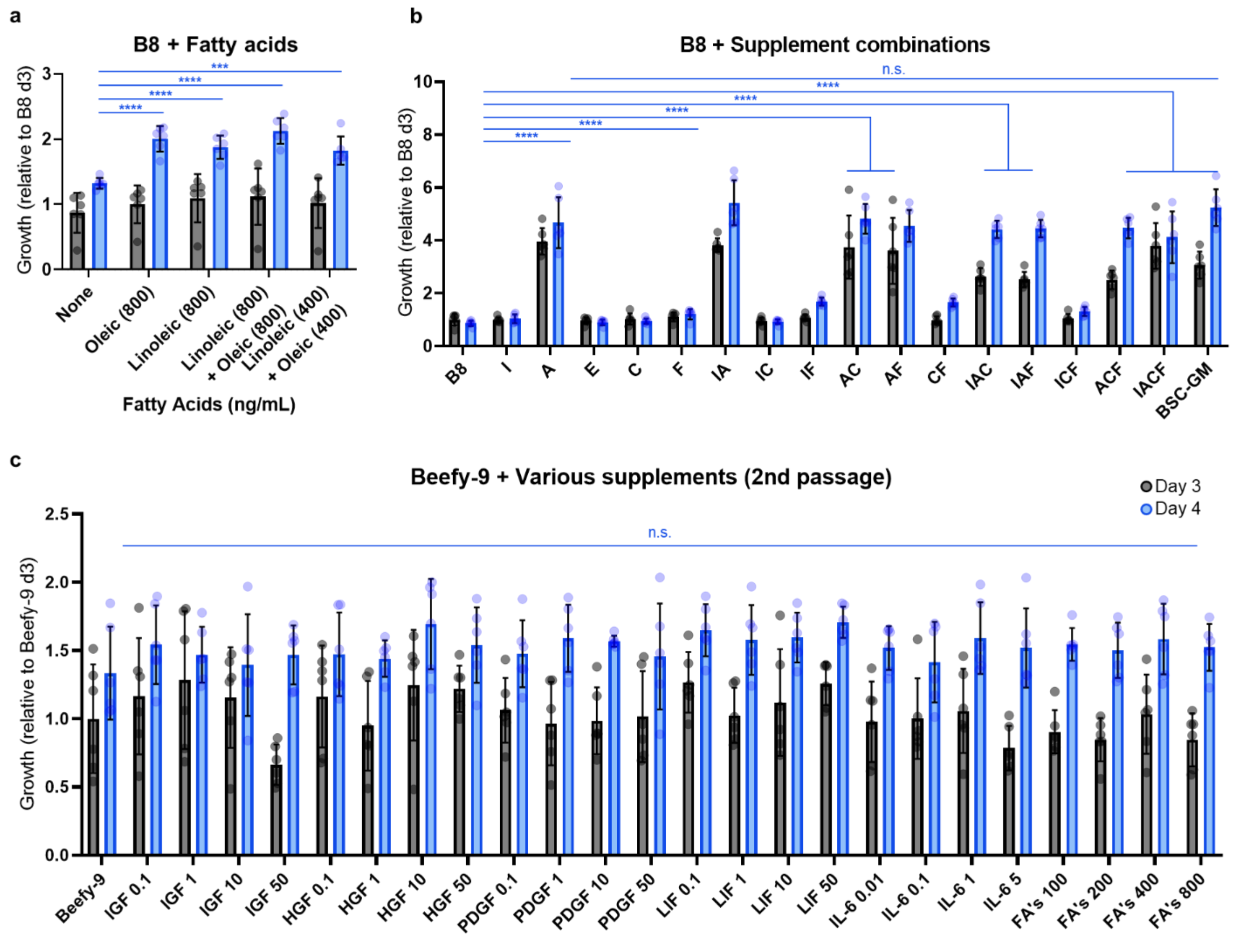

Figure S4: Short-term growth in B8 media supplemented with combinations of supplements. (a) BSC proliferation over 3 & 4 days B8 supplemented with combinations of fatty acids. Values are fluorescence based and given relative to day 3 B8 values. n=6 distinct samples; statistical significance was calculated by one-way ANOVA on day 4 data with multiple comparisons between supplemented samples and B8 controls, and is indicated by asterisks, in which $p < 0.05$ (*), $p < 0.01$ (**), $p < 0.001$ (***), and $p < 0.0001$ (****). **(b)** BSC proliferation over3 & 4 days in B8 supplemented with combinations of factors, in which: B8 = B8; I = IL-6 (.01 ng/mL); A = rAlbumin (800 µg/mL); C = Curcumin (1 ng/mL); F= linoleic acid (400 ng/mL) and Oleic acid (400 ng/mL); and BSC-GM = serum-containing growth media. n=6 distinct samples; statistical significance was calculated by one-way ANOVA on day 4 data with multiple comparisons between all samples, and is indicated by asterisks, in which $p < 0.05$ (*), $p < 0.01$ (**), $p < 0.001$ (***), and $p < 0.0001$ (****). Statistically significant differences between B8 and other samples are shown, as is insignificance between rAlbumin supplemented B8 and BSC-GM ($p>0.9990$). **(c)** BSC proliferation over 3 & 4 days in Beefy-9 supplemented with various factors (IGF = insulin-like growth factor 1 at concentrations from 0.1—50 ng/mL; HGF = hepatocyte growth factor at concentrations from 0.1—50 ng/mL; PDGF = platelet derived growth factor BB at concentrations from 0.1—50 ng/mL; LIF = leukemia inhibitory factor at concentrations from 0.1—50 ng/mL; IL-6 = interleukin 6 at concentrations from 0.01—5 ng/mL, FA's = Combinations of Linoleic & Oleic acid at concentrations ranging from 100-800 ng/mL of each fatty acid). Cells were passaged in B8/Beefy-9 media before testing in order to eliminate the potential for confounding release of adsorbed serum-derived growth factors from tissue culture plastic. n=6 distinct samples; statistical significance was calculated by one-way ANOVA on day 4 data with multiple comparisons between supplemented samples and Beefy-9 controls, and is indicated by asterisks, in which $p < 0.05$ (*), $p < 0.01$ (**), $p < 0.001$ (***), and $p < 0.0001$ (****).

Supplementary Video 1: Video of cell growth and differentiation
****See video file included in Data Files ****

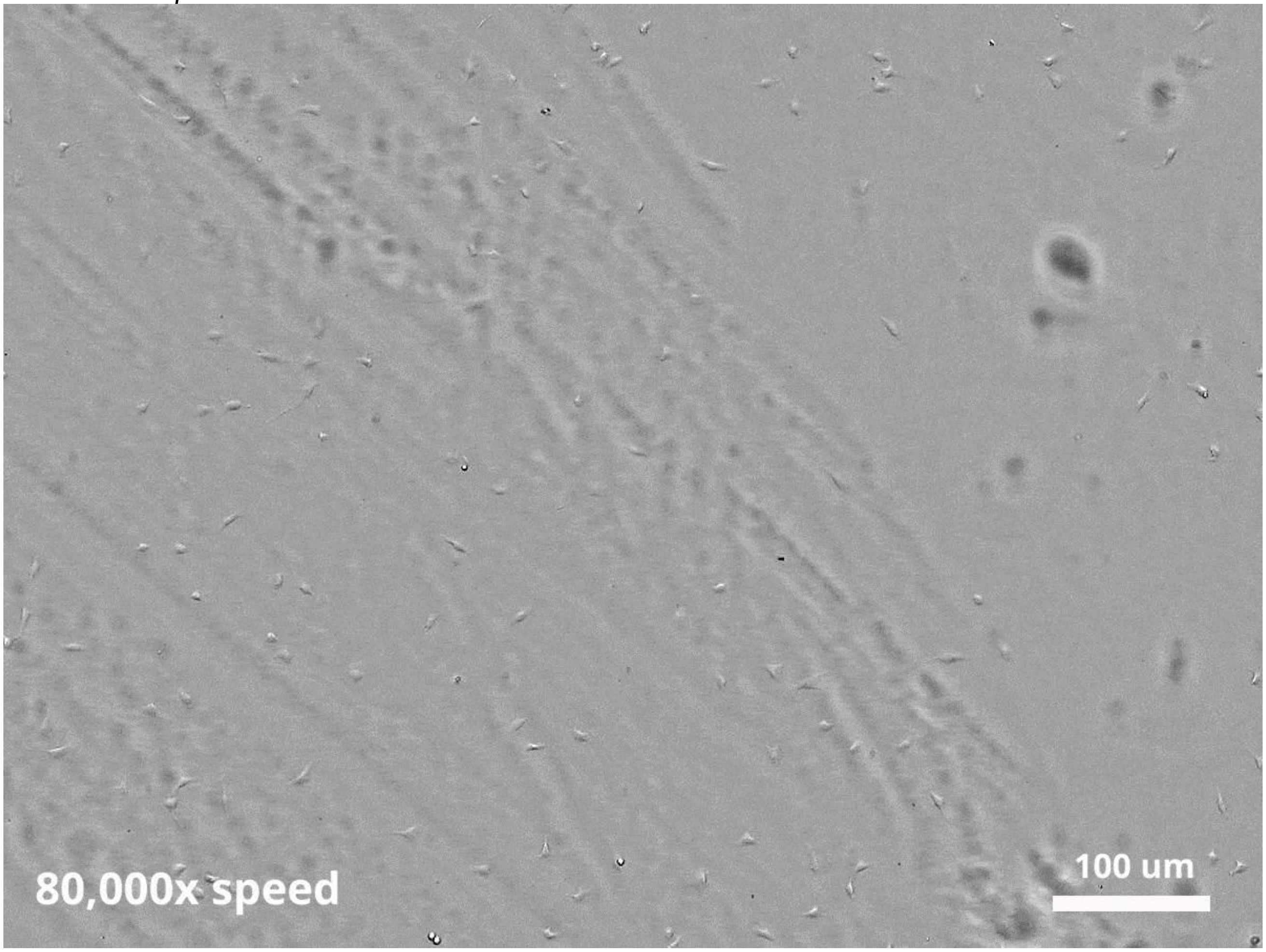

Video of cell growth in Beefy-9 after passaging onto vitronectin-coated plates demonstrates a completely animal-component free culture process for BSCs. Cells were harvested from previous culture vessels with animal-component-free TrypLE, seeding onto new flasks with animal-component-free recombinant vitronectin, proliferated with animal-component-free Beefy-9 media, and differentiated with an animal-component-free differentiation medium[1]. Cells were visualized on an Olympus Provi CM20 over 10 days with imaging every three hours.

Supplementary Figure 5: Multi-passage staining of sub-confluent muscle stem cells

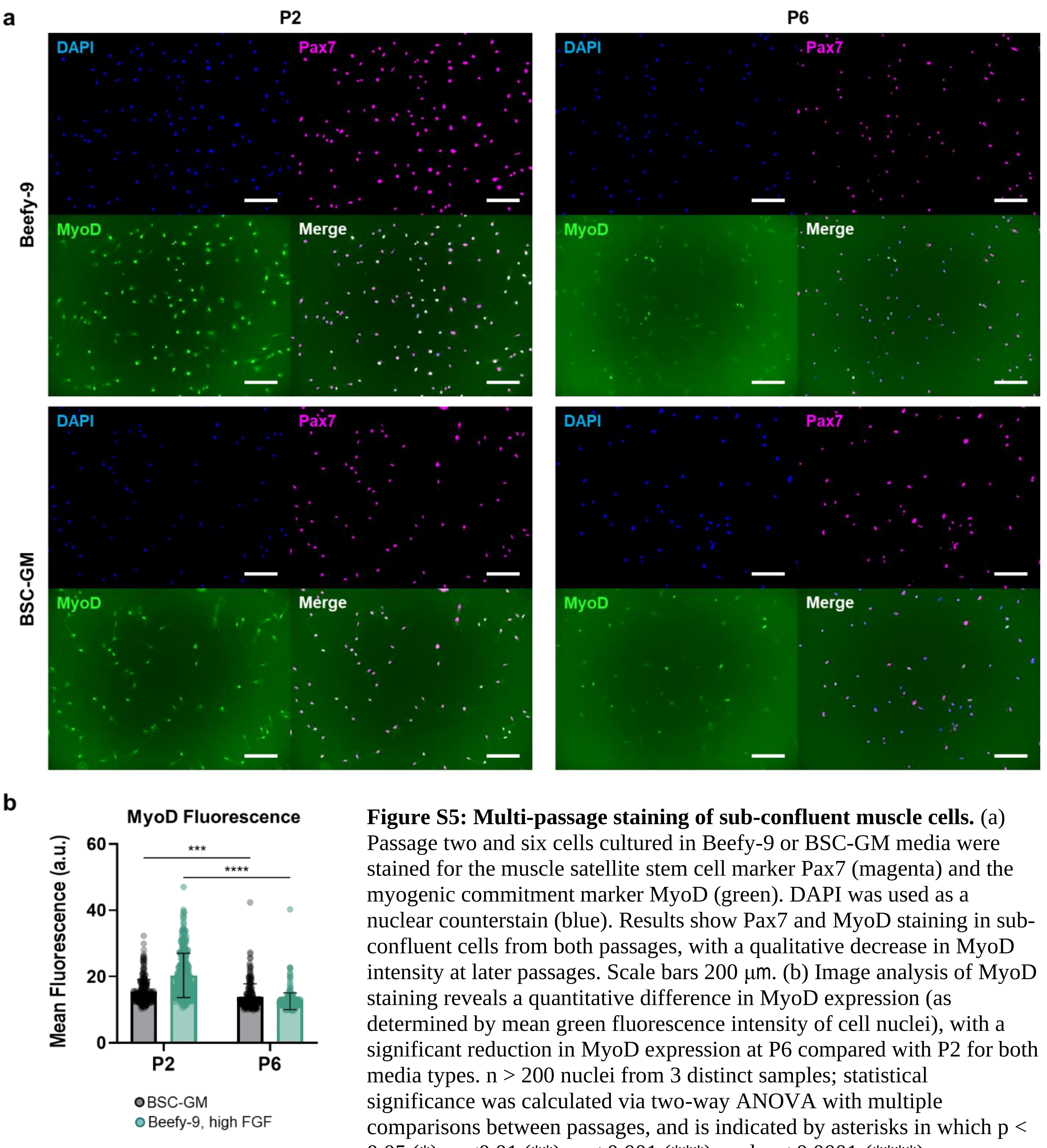

Figure S5: Multi-passage staining of sub-confluent muscle cells. (a) Passage two and six cells cultured in Beefy-9 or BSC-GM media were stained for the muscle satellite stem cell marker Pax7 (magenta) and the myogenic commitment marker MyoD (green). DAPI was used as a nuclear counterstain (blue). Results show Pax7 and MyoD staining in sub-confluent cells from both passages, with a qualitative decrease in MyoD intensity at later passages. Scale bars 200 μm. (b) Image analysis of MyoD staining reveals a quantitative difference in MyoD expression (as determined by mean green fluorescence intensity of cell nuclei), with a significant reduction in MyoD expression at P6 compared with P2 for both media types. n > 200 nuclei from 3 distinct samples; statistical significance was calculated via two-way ANOVA with multiple comparisons between passages, and is indicated by asterisks in which $p < 0.05$ (*), $p < 0.01$ (**), $p < 0.001$ (***), and $p < 0.0001$ (****).

Supplementary Figure 6: Multi-passage staining of differentiated muscle stem cells

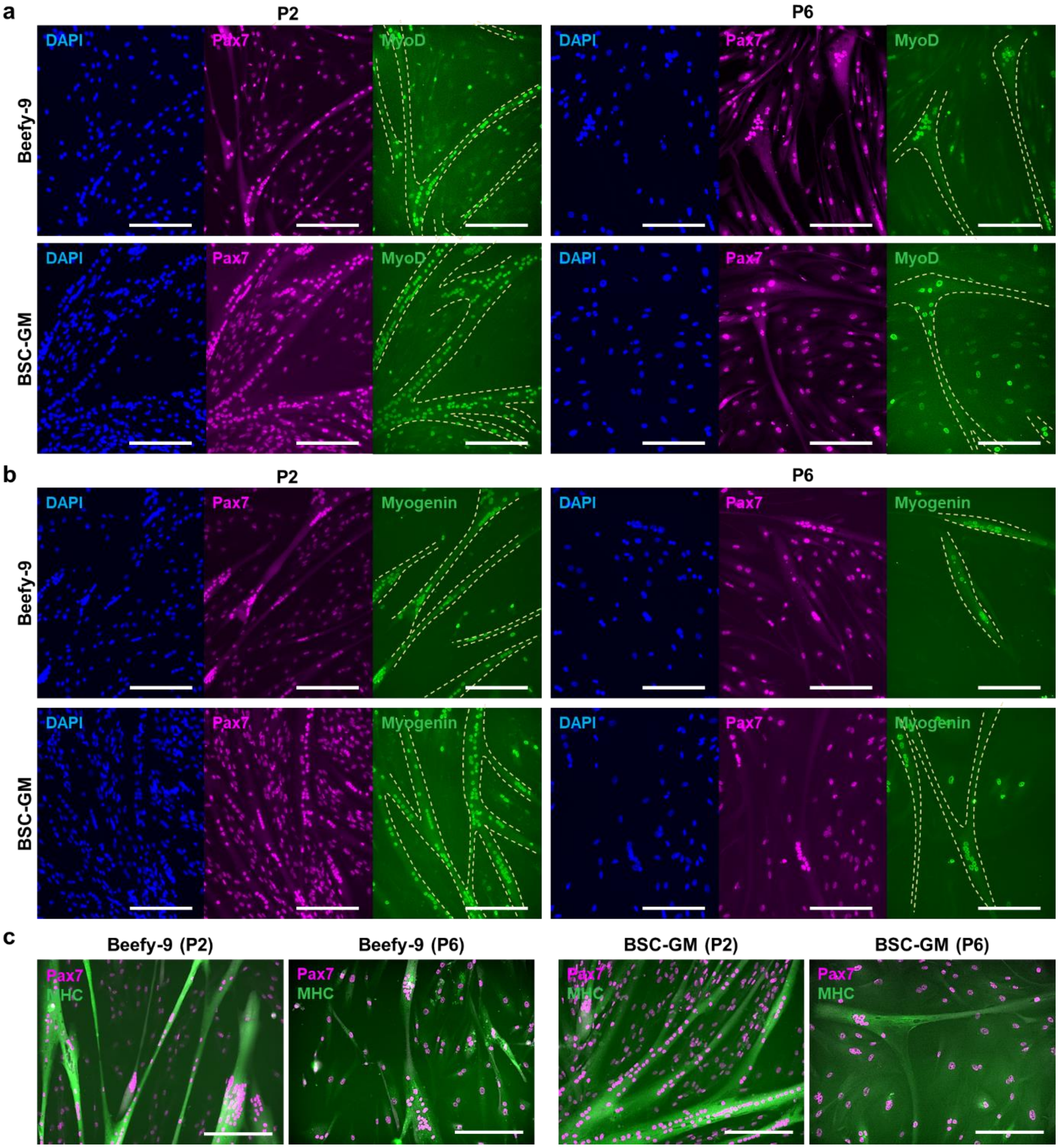

Figure S6: Multi-passage staining of differentiated muscle cells. (a) Cells from P2 and P6 cultured in Beefy-9 and BSC-GM were differentiated as before and stained for Pax7 (magenta), the myogenic commitment marker MyoD (green), and nuclei (DAPI; blue). Differentiated myotubes are indicated with dashed yellow lines. (b) Cells treated as above were stained for the Pax7 (magenta), the fusion regulating factor Myogenin (green), and nuclei (DAPI; blue). Differentiated myotubes are indicated with dashed yellow lines. Results show Myogenin+ cells primarily located within multinucleated myotubes. (c) Cells treated as above were stained for myosin heavy chain (MHC; green) and Pax7 (magenta). Results show MHC-negative cells still maintain Pax7 expression. Scale bars 200 µm.

Supplementary Figure 7: Fusion index analysis of differentiated BSCs

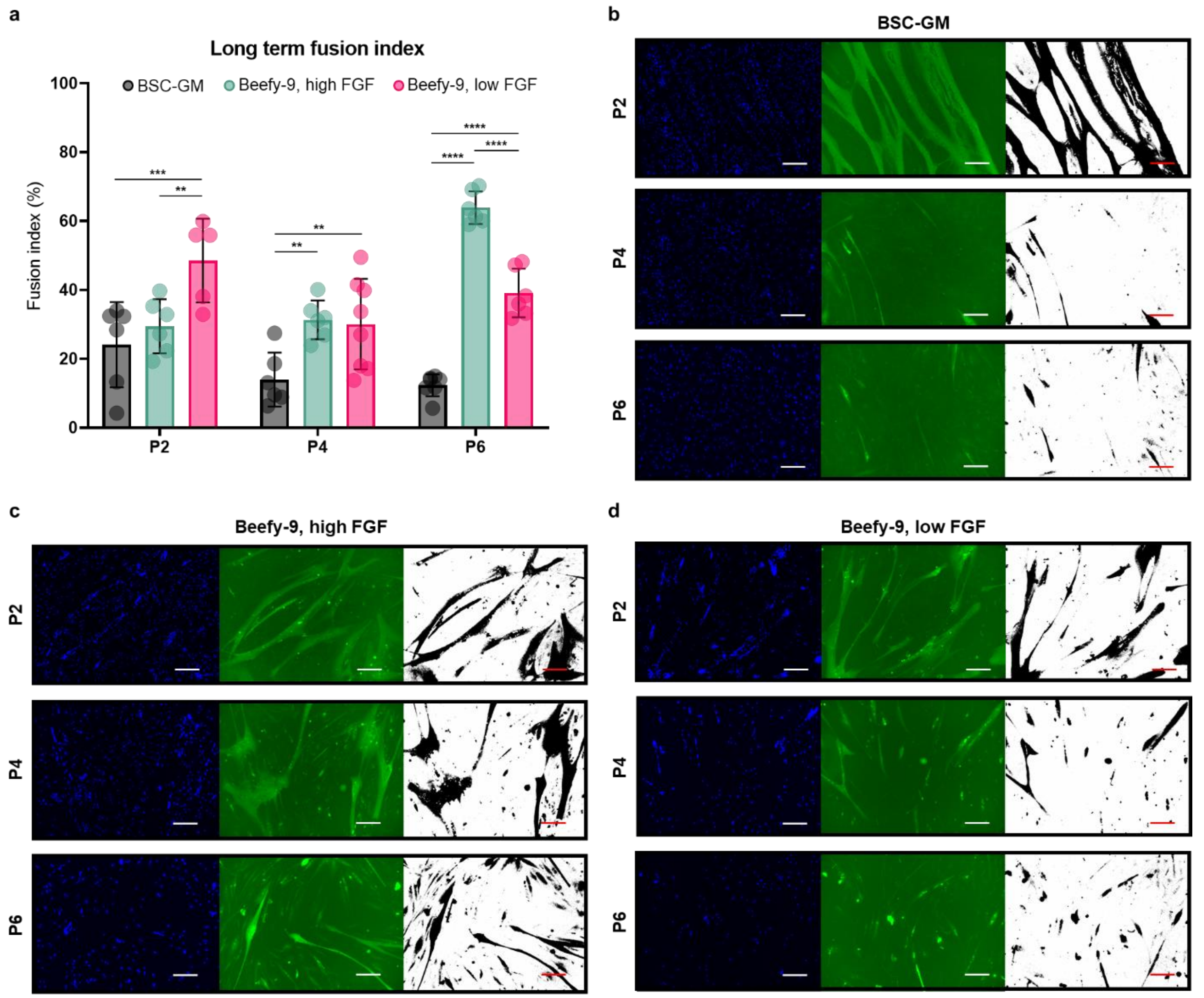

Figure S7: Fusion index analysis of BSCs cultured over six passages in various media. (a) Fusion index of images (10x magnification) of BSCs cultured in various media and differentiated for 4 days (P2 and P4) or 6 days (P6). Fusion index given as the percent of imaged nuSclei in the images that are superimposed with myosin heavy chain (MHC) staining after thresholding in ImageJ (threshold value = 18 for all images). Results suggest improved differentiation in serum-free cultures; however, it is possible that reduced total cell number in serum-free conditions contribute to this conclusion, rather than solely differentiation extent. n=5-8 distinct images depending on whether any images were excluded due to imaging artifacts (e.g., bubbles) that clearly disrupted image analysis; statistical significance was calculated by two-way ANOVA with multiple comparisons between media types for each distinct passage tested, and is indicated by asterisks, in which $p < 0.05$ (*), $p < 0.01$ (**), $p < 0.001$ (***), and $p < 0.0001$ (****). **(b-d)** Representative images (Left = DAPI; Middle = Myosin Heavy Chain (MHC); Right = MHC post-thresholding) of those used for generating figure S4(A) for each media type and passage number tested. Scale bars 100 µm.

Supplementary Figure 8: Short-term growth with additional rAlbumin

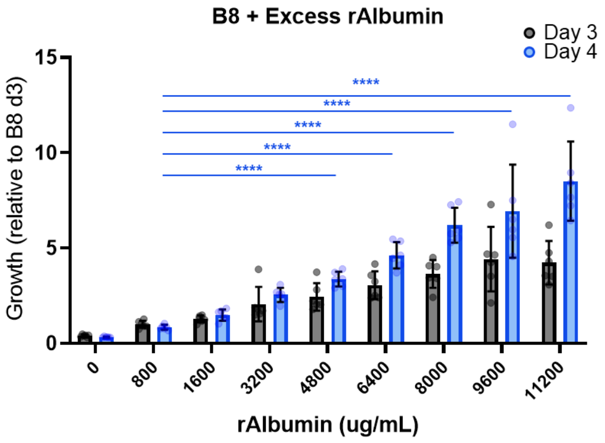

Figure S7: Short-term growth in Beefy-9 with additional rAlbumin. BSC proliferation over 3 & 4 days in increased concentrations of rAlbumin (over 800 ug/mL, as was formulated for the original Beefy-9 medium. Results show an drastic increase in growth with increasing rAlbumin concentration. Indeed, while 11.2 mg/mL was the maximum concentration tested, this was not the limit for improving short-term growth. n=6 distinct samples; statistical significance was calculated by one-way ANOVA on day 4 data comparing all samples with Beefy-9 ("800"), and is indicated by asterisks, in which $p < 0.05$ (*), $p < 0.01$ (**), $p < 0.001$ (***), and $p < 0.0001$ (****).

Supplementary Table 3: Component sourcing and pricing for cost analysis

BSC-GM															
This study								Bulk							
Component	Unit/L	Amnt/L	Supplier	Cat. #	Units/order	Cost/order	Cost/L	Component	Unit/L	Amnt/L	Supplier	Cat. #	Units/order	Cost/order	Cost/L
DMEM (Liquid)<br>+ HEPES<br>+ Sodium Bicarbonate	mL	800	ThermoFisher	10569044	5,000	294	47.04	DMEM (Powder)	mL	800	ThermoFisher	12100061	10000	27.88	2.23
								HEPES	g	3.574	Grainger	30TY36	10000	1060.8	0.38
								Sodium Bicarbonate	g	1.2	Grainger	31GD57	5000	120.59	0.03
FBS	mL	200	ThermoFisher	26140079	500	606	242.40	FBS	mL	200	Zenbio	SER-500	500	452	180.80
FGF-2	ug	1	Peprotech	100-18B	1000	800	0.80	FGF2	ug	1	Peprotech	100-18B	1000	800	0.80
**Total**							**290.24**	**Total**							**184.24**

Bt9 (store-bought growth factors)															
This study								Bulk							
Component	Unit/L	Amnt/L	Supplier	Cat. #	Units/order	Cost/order	Cost/L	Component	Unit/L	Amnt/L	Supplier	Cat. #	Units/order	Cost/order	Cost/L
DMEM/F12 (Liquid)<br>+ HEPES<br>+ Sodium Bicarbonate	mL	1000	ThermoFisher	11330057	5,000	334	66.80	DMEM/F12 (Powder)	mL	1000	ThermoFisher	12500096	50000	189	3.78
								HEPES	g	3.574	Grainger	30TY36	10000	1060.8	0.38
								Sodium Bicarbonate	g	1.2	Grainger	31GD57	5000	120.59	0.03
L-ascorbic acid 2-phosphate	mg	200	Sigma	49752-10G	10000	65.5	1.31	L-ascorbic acid 2-phosphate	mg	200	Sigma	49752-100G	100000	417	0.83
Insulin	mg	20	Sigma	91077C-250MG	250	254	20.32	Insulin	mg	20	InVitria	777HSA017S-	100000	41920	8.38
Transferrin	mg	20	inVitria	777TRF029	1000	260.5	5.21	Transferrin	mg	20	inVitria	777TRF029	10000	1823	3.65
Sodium selenite	ug	20	Sigma	S5261-10G	10000000	32.1	0.00	Sodium selenite	ug	20	Sigma	S5261-100G	100000000	32.1	0.00
FGF2	ug	40	Peprotech	100-18B	1000	800	32.00	FGF2	ug	40	Peprotech	100-18B	1000	800	32.00
NRG1	ng	100	Peprotech	100-03	10000	80	0.80	NRG1	ng	100	PeproTech	100-03	1000000	1500	0.15
TGFb3	ng	100	R&D Systems	8420-B3-005/CF	5000	199	3.98	TGFb3	ng	100	PeproTech	100-36E	1000000	5200	0.52
rAlbumin	mg	800	Sigma	A9731-1G	1000	108	86.40	rAlbumin	mg	800	ScienCell	OsrHSA	1000000	30703	24.56
**Total**							**216.82**	**Total**							**74.28**

Bt9 (Low FGF)															
This study								Bulk							
Component	Unit/L	Amnt/L	Supplier	Cat. #	Units/order	Cost/order	Cost/L	Component	Unit/L	Amnt/L	Supplier	Cat. #	Units/order	Cost/order	Cost/L
DMEM/F12 (Liquid)<br>+ HEPES<br>+ Sodium Bicarbonate	mL	1000	ThermoFisher	11330057	5,000	334	66.80	DMEM/F12, Powder	mL	1000	ThermoFisher	12500096	50000	189	3.78
								HEPES	g	3.574	Grainger	30TY36	10000	1060.8	0.38
								Sodium Bicarbonate	g	1.2	Grainger	31GD57	5000	120.59	0.03
L-ascorbic acid 2-phosphate	mg	200	Sigma	49752-10G	10000	65.5	1.31	L-ascorbic acid 2-phosphate	mg	200	Sigma	49752-100G	100000	417	0.83
Insulin	mg	20	Sigma	91077C-250MG	250	254	20.32	Insulin	mg	20	InVitria	777HSA017S-	100000	41920	8.38
Transferrin	mg	20	inVitria	777TRF029	1000	260.5	5.21	Transferrin	mg	20	inVitria	777TRF029	10000	1823	3.65
Sodium selenite	ug	20	Sigma	S5261-10G	10000000	32.1	0.00	Sodium selenite	ug	20	Sigma	S5261-100G	100000000	32.1	0.00
FGF2	ug	5	Peprotech	100-18B	1000	800	4.00	FGF2	ug	5	Peprotech	100-18B	1000	800	4.00
NRG1	ng	100	Peprotech	100-03	10000	80	0.80	NRG1	ng	100	PeproTech	100-03	1000000	1500	0.15
TGFb3	ng	100	R&D Systems	8420-B3-005/CF	5000	199	3.98	TGFb3	ng	100	PeproTech	100-36E	1000000	5200	0.52
rAlbumin	mg	800	Sigma	A9731-1G	1000	108	86.40	rAlbumin	mg	800	ScienCell	OsrHSA	1000000	30703	24.56
**Total**							**188.82**	**Total**							**46.28**
