# Supplementary material for: Simple and effective serum-free medium for sustained expansion of bovine satellite cells for cell cultured meat
Source: Commun Biol. 2022 Jun 2;5:466. doi: 10.1038/s42003-022-03423-8 (PMC9163123; doi:10.1038/s42003-022-03423-8)
Supplement: Supplementary file 2 — Description of Additional Supplementary Files [file 42003_2022_3423_MOESM2_ESM.pdf]

Description of Additional Supplementary Files

File name: Supplementary Video 1
Description: Video of cell growth and differentiation.
