# Supplementary figures and images for: Simple and effective serum-free medium for sustained expansion of bovine satellite cells for cell cultured meat (part 1 of 2)
Source: Commun Biol. 2022 Jun 2;5:466. doi: 10.1038/s42003-022-03423-8 (PMC9163123; doi:10.1038/s42003-022-03423-8)

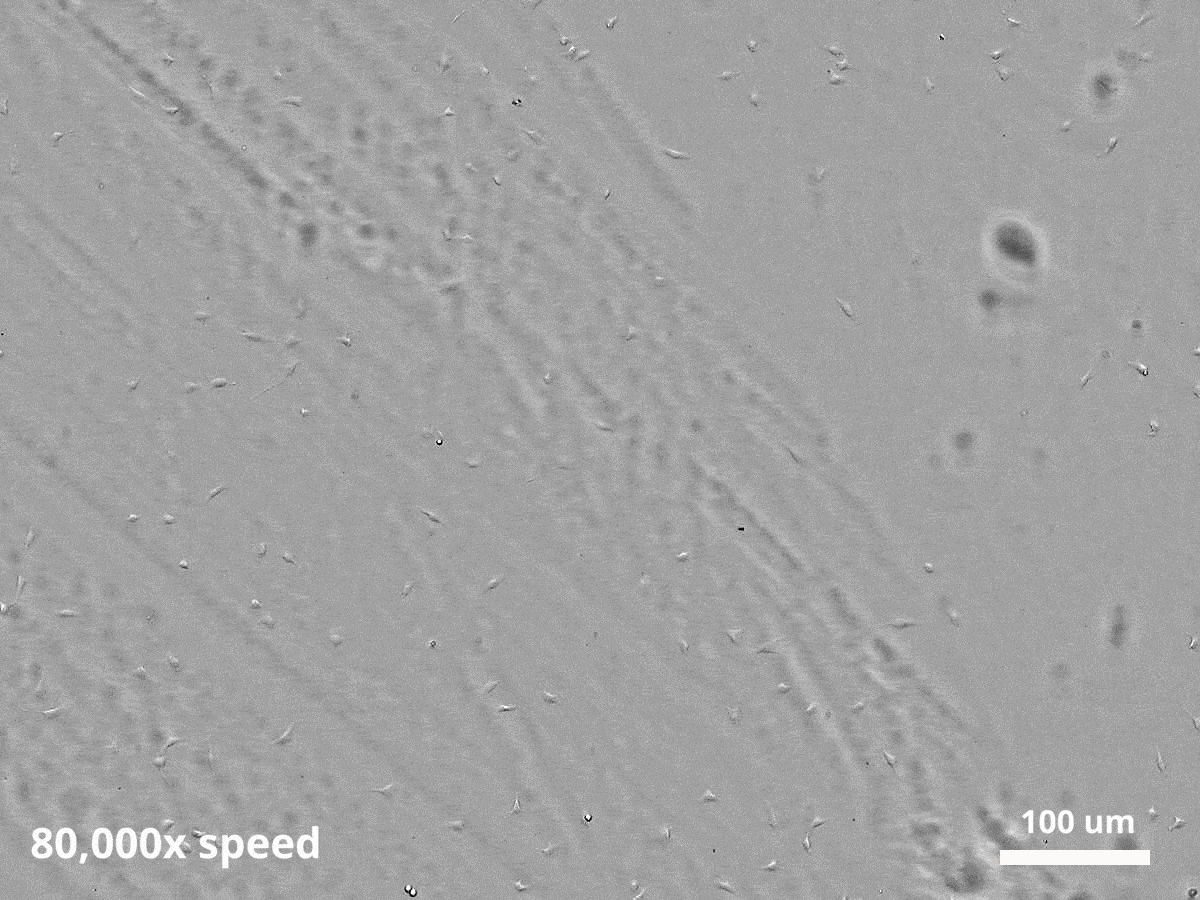

Supplement: Supplementary file 3 — Supplementary Video 1 [file 42003_2022_3423_MOESM3_ESM.gif]

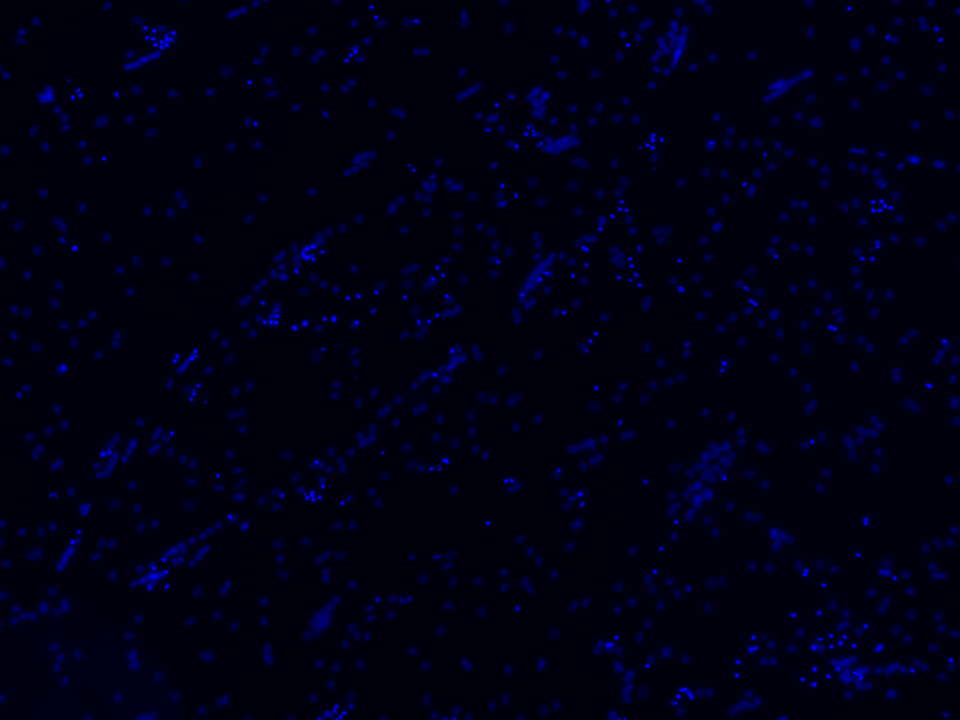

Supplement: Supplementary file 4 — Supplementary Data 1 [file 42003_2022_3423_MOESM4_ESM.zip › Figure S7 (Images)/Raw image files/10x BSC2 P2 Beefy-9 high FGF/XY01/image_XY01_CH1.tif]

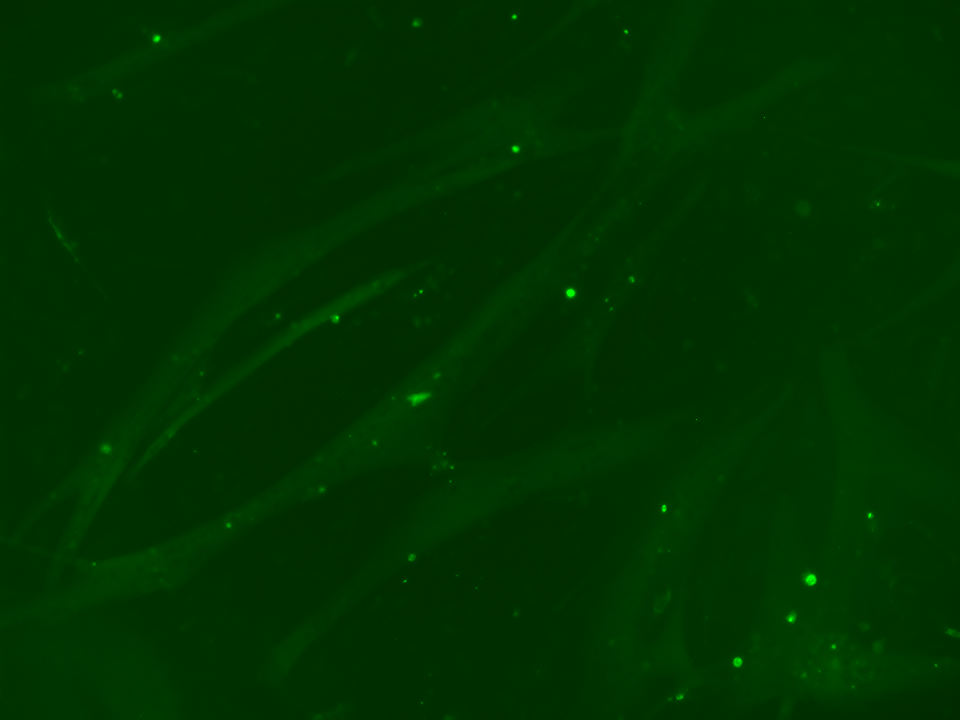

Supplement: Supplementary file 4 — Supplementary Data 1 [file 42003_2022_3423_MOESM4_ESM.zip › Figure S7 (Images)/Raw image files/10x BSC2 P2 Beefy-9 high FGF/XY01/image_XY01_CH2.tif]

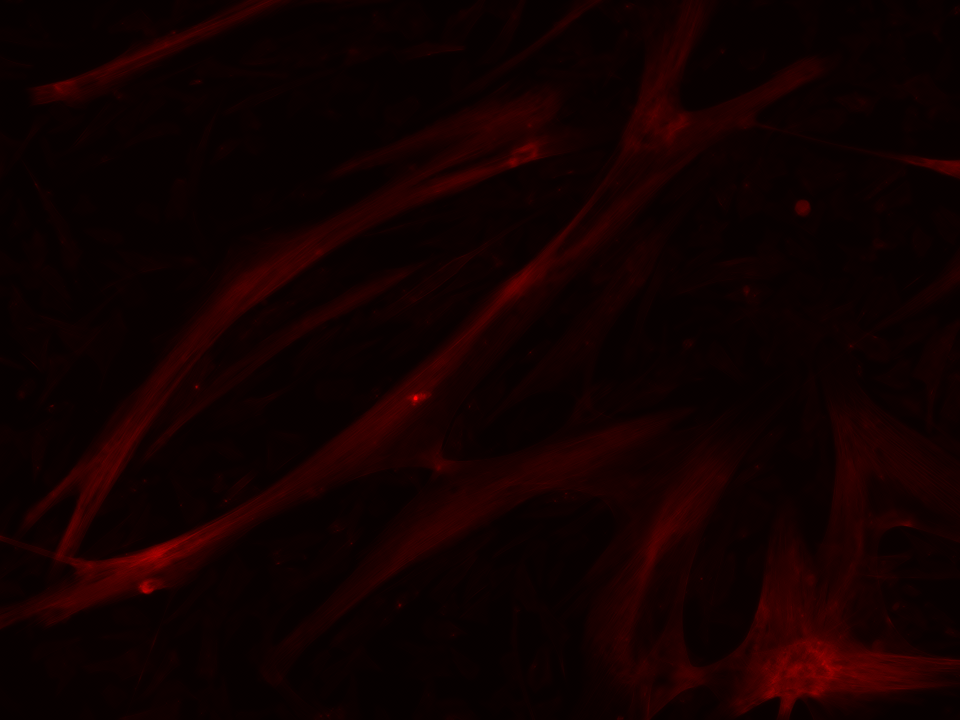

Supplement: Supplementary file 4 — Supplementary Data 1 [file 42003_2022_3423_MOESM4_ESM.zip › Figure S7 (Images)/Raw image files/10x BSC2 P2 Beefy-9 high FGF/XY01/image_XY01_CH3.tif]

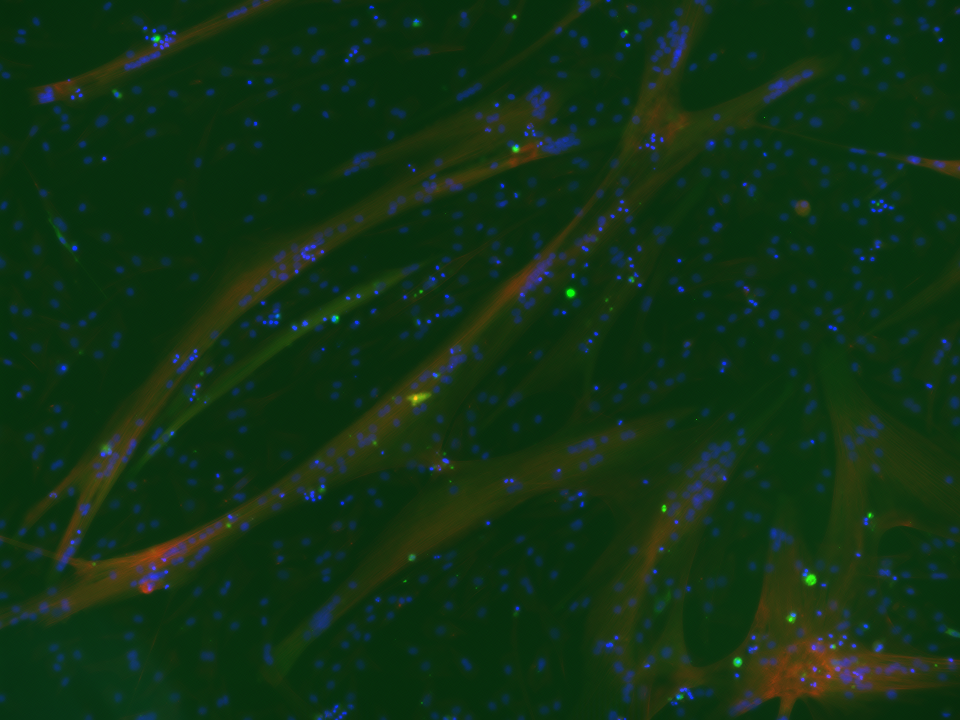

Supplement: Supplementary file 4 — Supplementary Data 1 [file 42003_2022_3423_MOESM4_ESM.zip › Figure S7 (Images)/Raw image files/10x BSC2 P2 Beefy-9 high FGF/XY01/image_XY01_Overlay.tif]

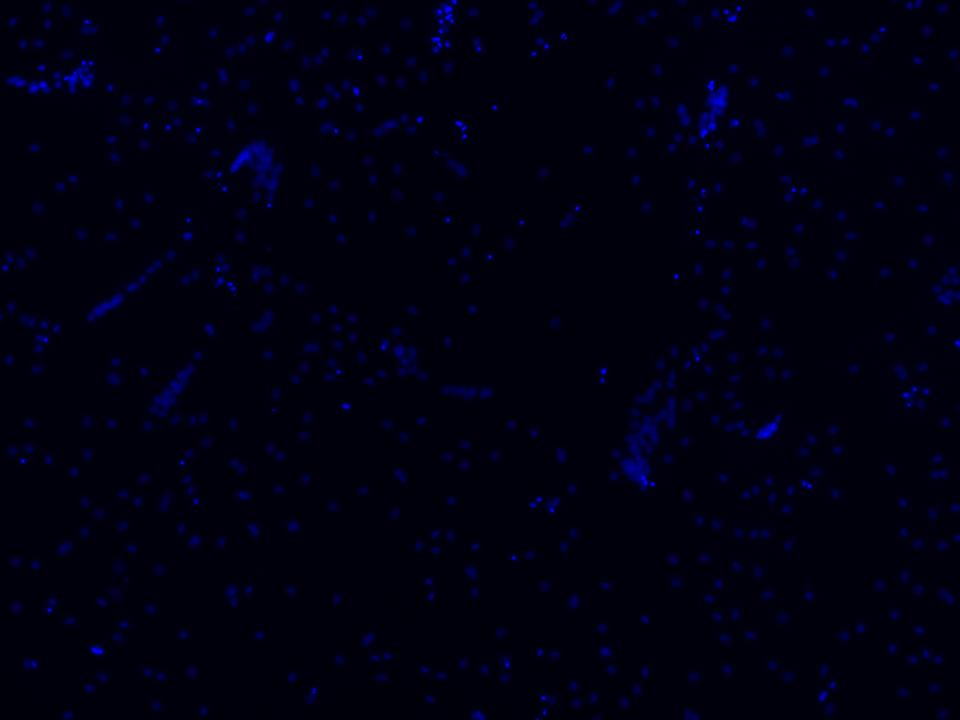

Supplement: Supplementary file 4 — Supplementary Data 1 [file 42003_2022_3423_MOESM4_ESM.zip › Figure S7 (Images)/Raw image files/10x BSC2 P2 Beefy-9 high FGF/XY02/image_XY02_CH1.tif]

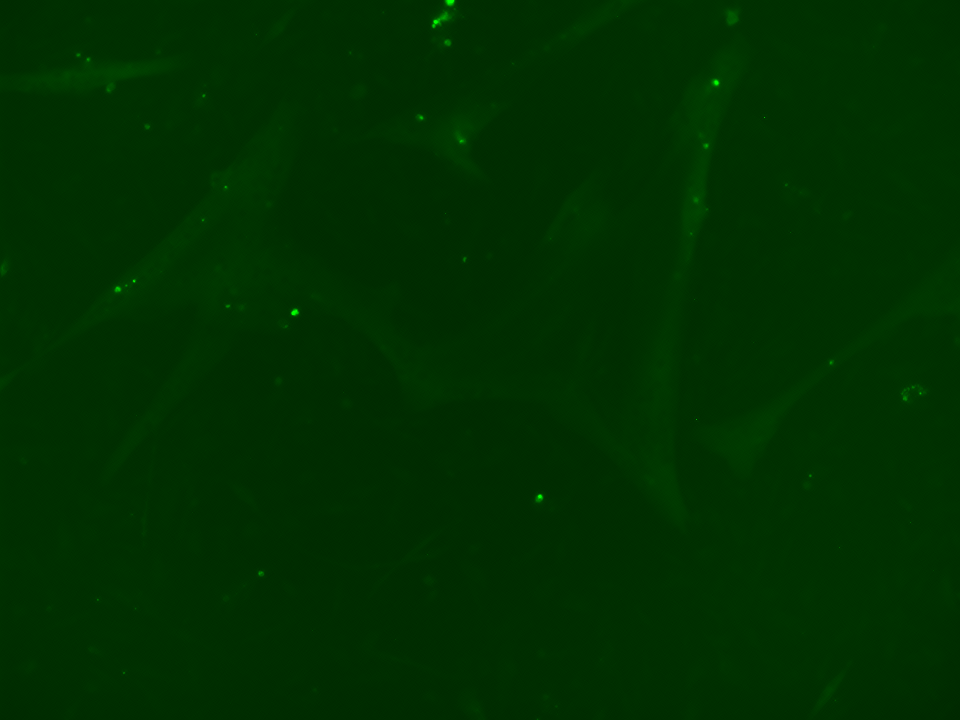

Supplement: Supplementary file 4 — Supplementary Data 1 [file 42003_2022_3423_MOESM4_ESM.zip › Figure S7 (Images)/Raw image files/10x BSC2 P2 Beefy-9 high FGF/XY02/image_XY02_CH2.tif]

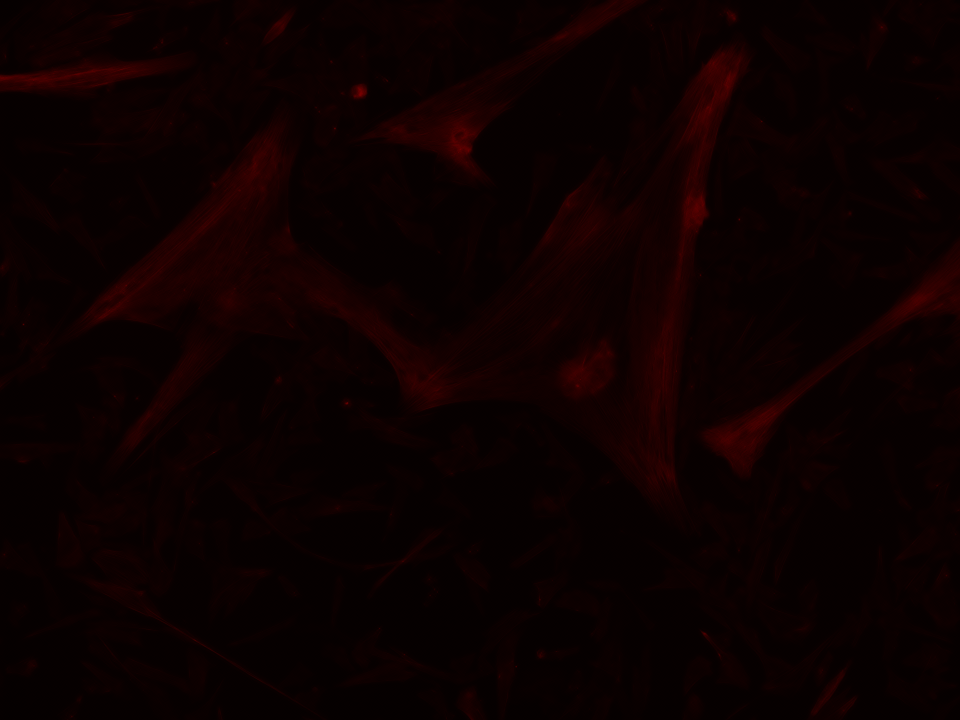

Supplement: Supplementary file 4 — Supplementary Data 1 [file 42003_2022_3423_MOESM4_ESM.zip › Figure S7 (Images)/Raw image files/10x BSC2 P2 Beefy-9 high FGF/XY02/image_XY02_CH3.tif]

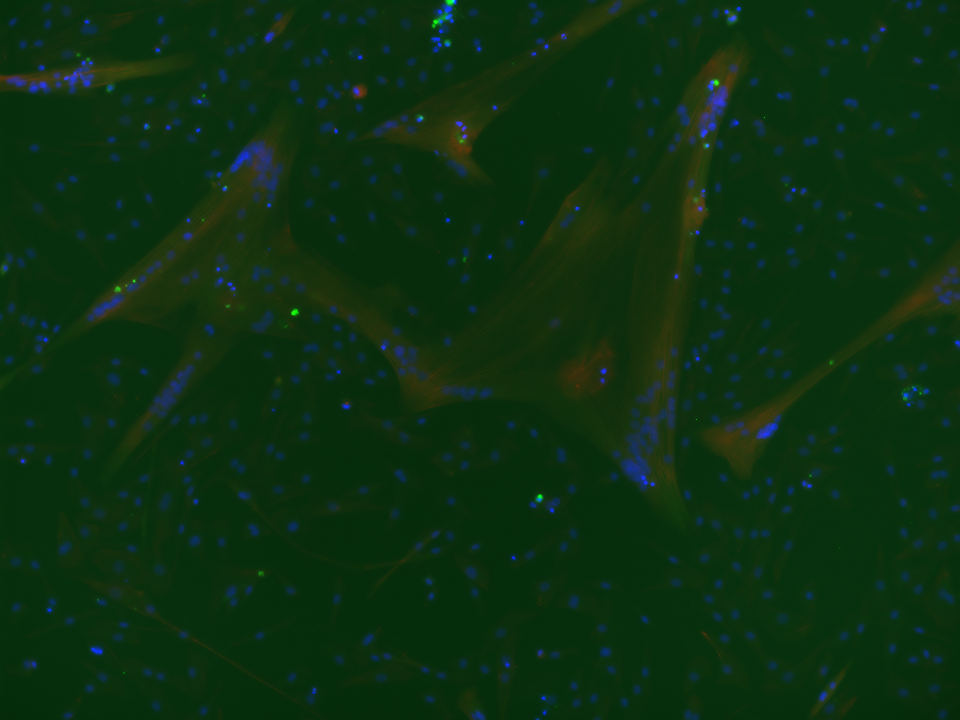

Supplement: Supplementary file 4 — Supplementary Data 1 [file 42003_2022_3423_MOESM4_ESM.zip › Figure S7 (Images)/Raw image files/10x BSC2 P2 Beefy-9 high FGF/XY02/image_XY02_Overlay.tif]

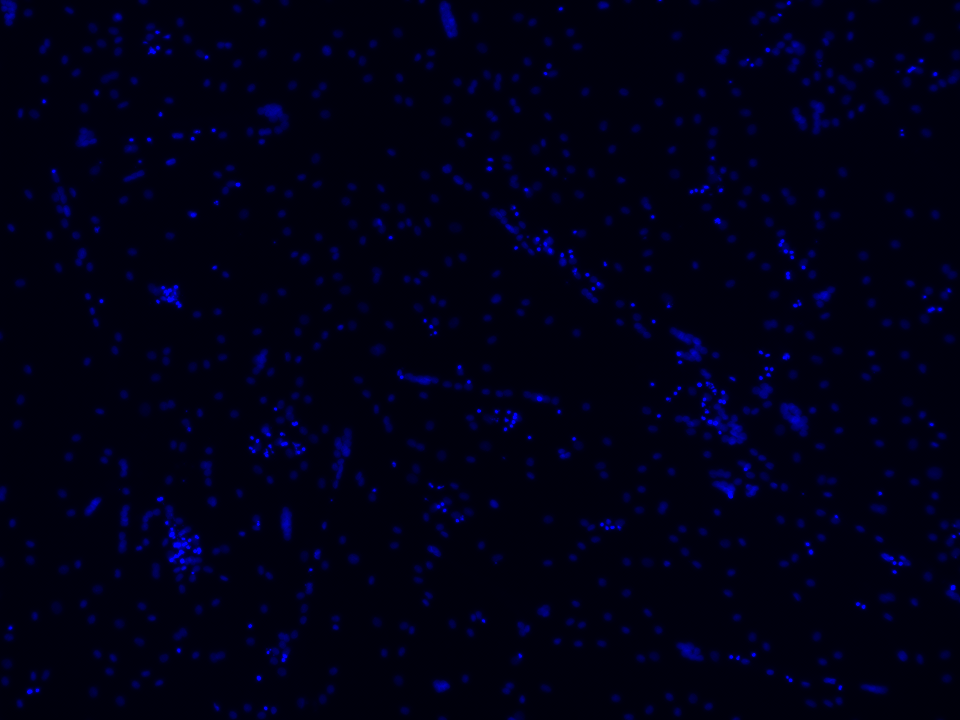

Supplement: Supplementary file 4 — Supplementary Data 1 [file 42003_2022_3423_MOESM4_ESM.zip › Figure S7 (Images)/Raw image files/10x BSC2 P2 Beefy-9 high FGF/XY03/image_XY03_CH1.tif]

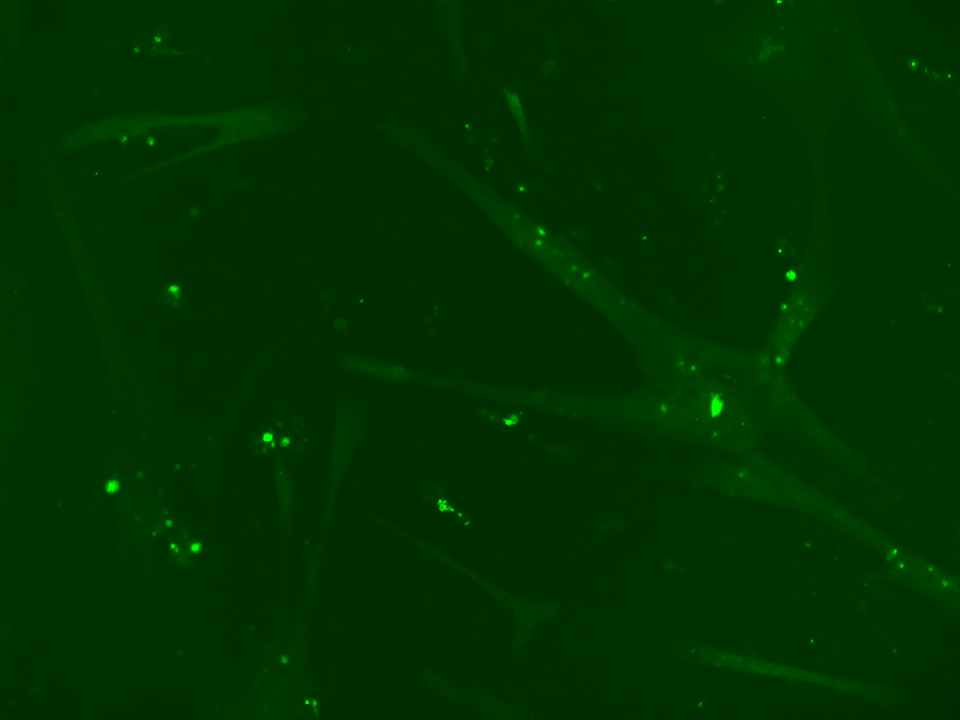

Supplement: Supplementary file 4 — Supplementary Data 1 [file 42003_2022_3423_MOESM4_ESM.zip › Figure S7 (Images)/Raw image files/10x BSC2 P2 Beefy-9 high FGF/XY03/image_XY03_CH2.tif]

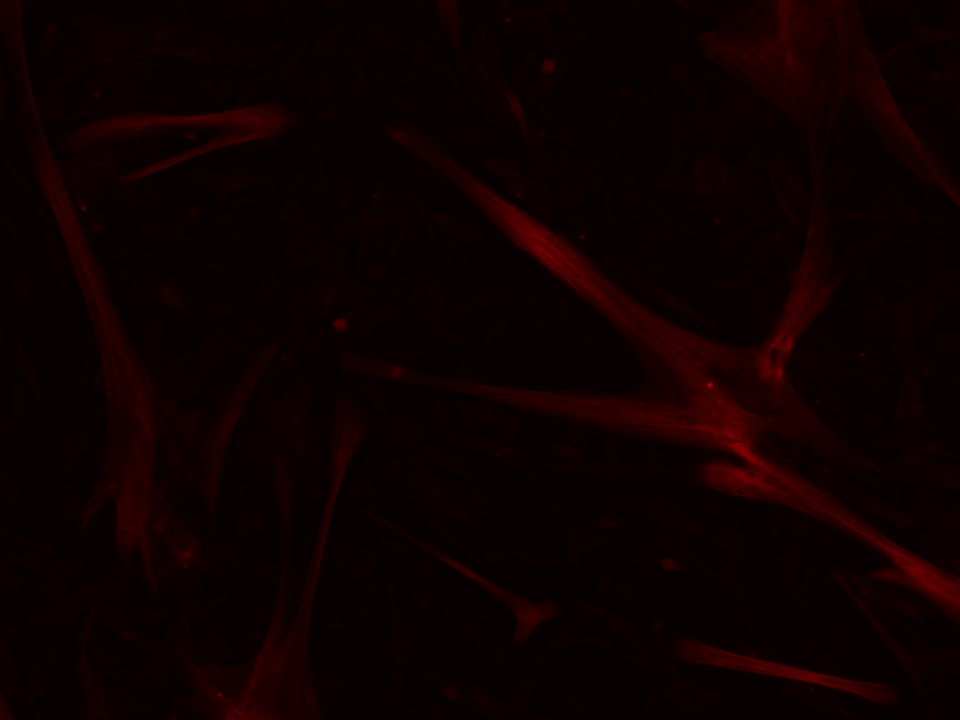

Supplement: Supplementary file 4 — Supplementary Data 1 [file 42003_2022_3423_MOESM4_ESM.zip › Figure S7 (Images)/Raw image files/10x BSC2 P2 Beefy-9 high FGF/XY03/image_XY03_CH3.tif]

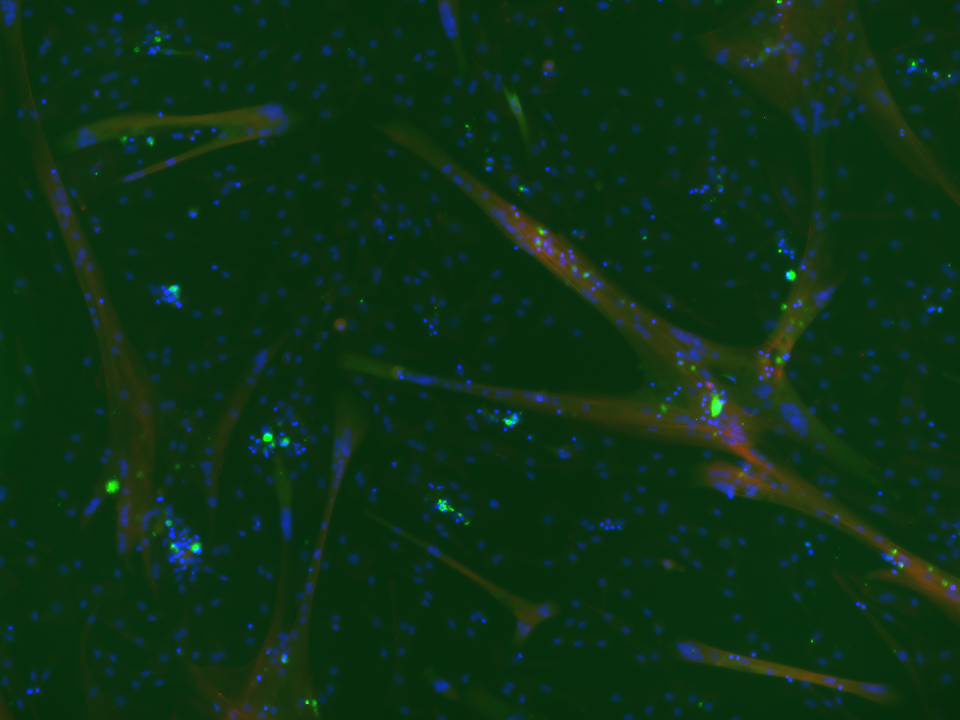

Supplement: Supplementary file 4 — Supplementary Data 1 [file 42003_2022_3423_MOESM4_ESM.zip › Figure S7 (Images)/Raw image files/10x BSC2 P2 Beefy-9 high FGF/XY03/image_XY03_Overlay.tif]

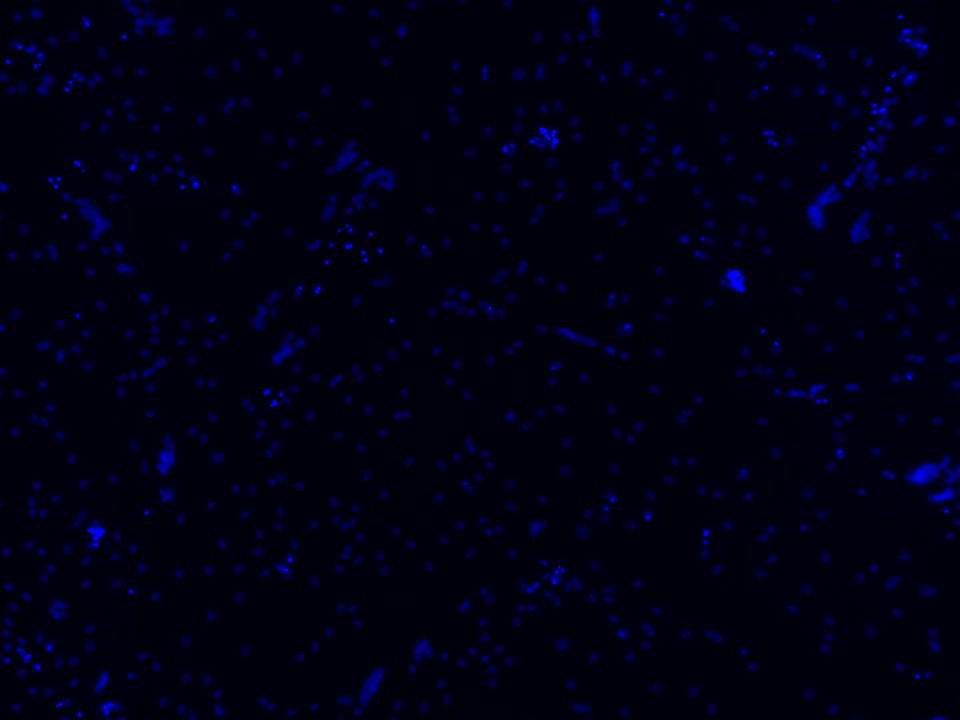

Supplement: Supplementary file 4 — Supplementary Data 1 [file 42003_2022_3423_MOESM4_ESM.zip › Figure S7 (Images)/Raw image files/10x BSC2 P2 Beefy-9 high FGF/XY04/image_XY04_CH1.tif]

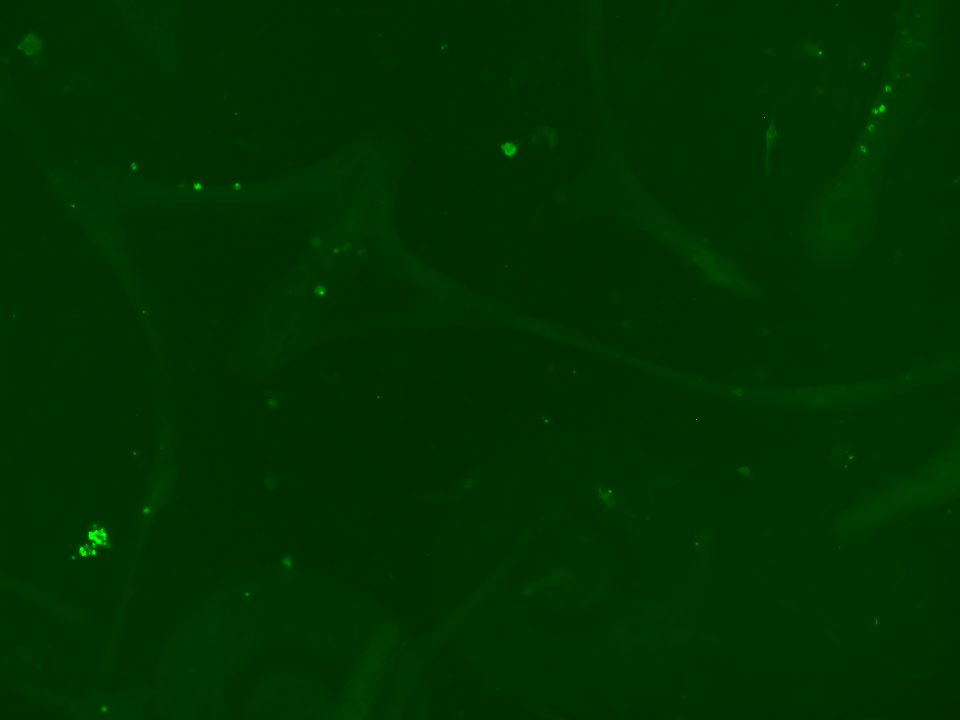

Supplement: Supplementary file 4 — Supplementary Data 1 [file 42003_2022_3423_MOESM4_ESM.zip › Figure S7 (Images)/Raw image files/10x BSC2 P2 Beefy-9 high FGF/XY04/image_XY04_CH2.tif]

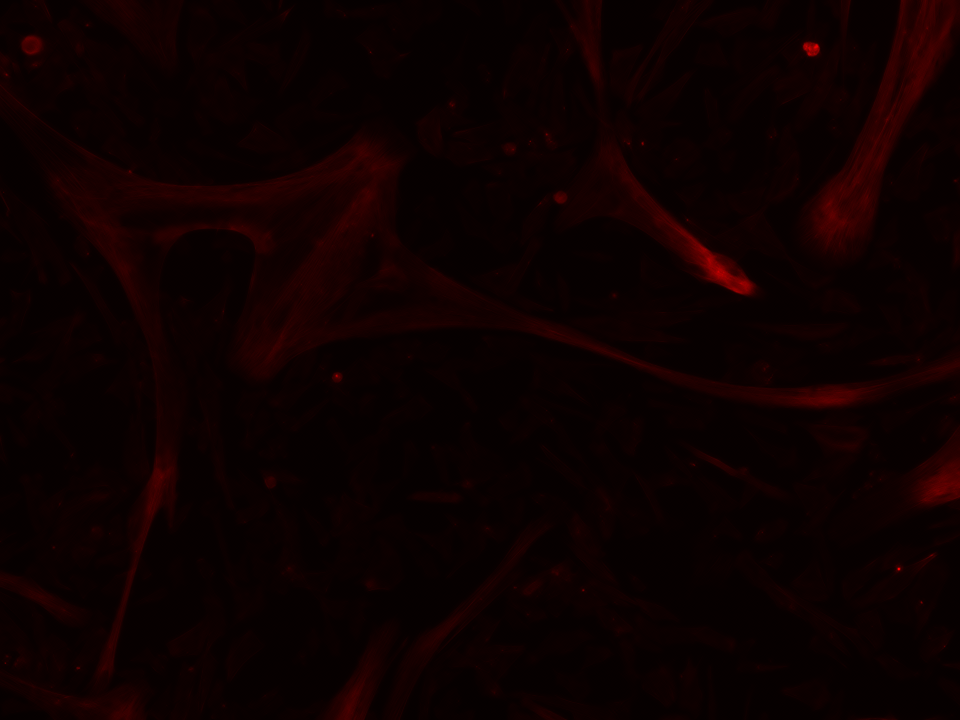

Supplement: Supplementary file 4 — Supplementary Data 1 [file 42003_2022_3423_MOESM4_ESM.zip › Figure S7 (Images)/Raw image files/10x BSC2 P2 Beefy-9 high FGF/XY04/image_XY04_CH3.tif]

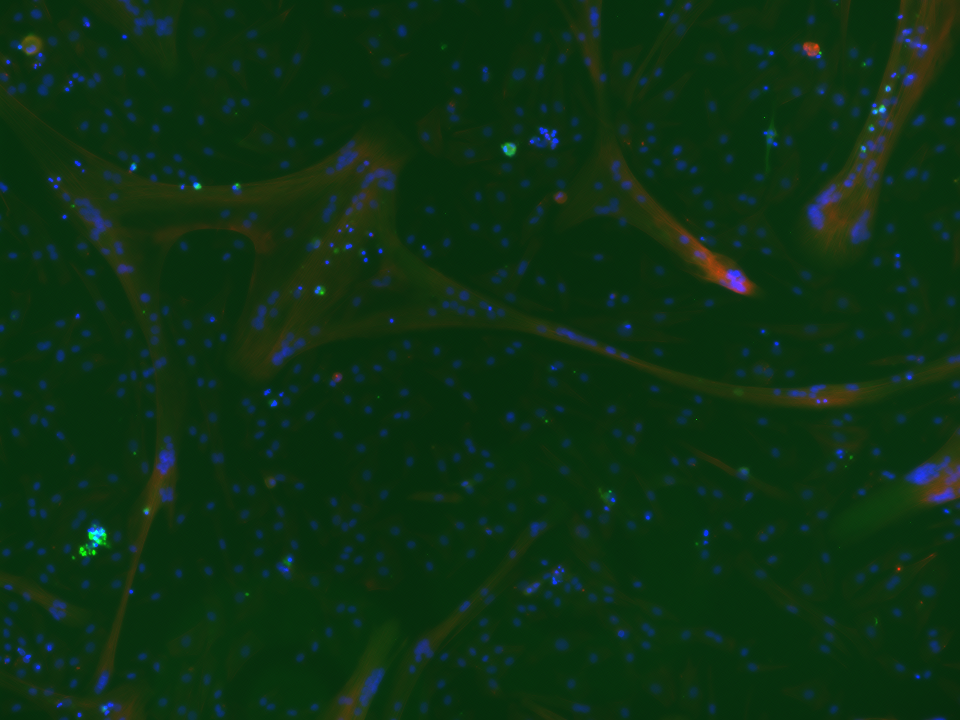

Supplement: Supplementary file 4 — Supplementary Data 1 [file 42003_2022_3423_MOESM4_ESM.zip › Figure S7 (Images)/Raw image files/10x BSC2 P2 Beefy-9 high FGF/XY04/image_XY04_Overlay.tif]

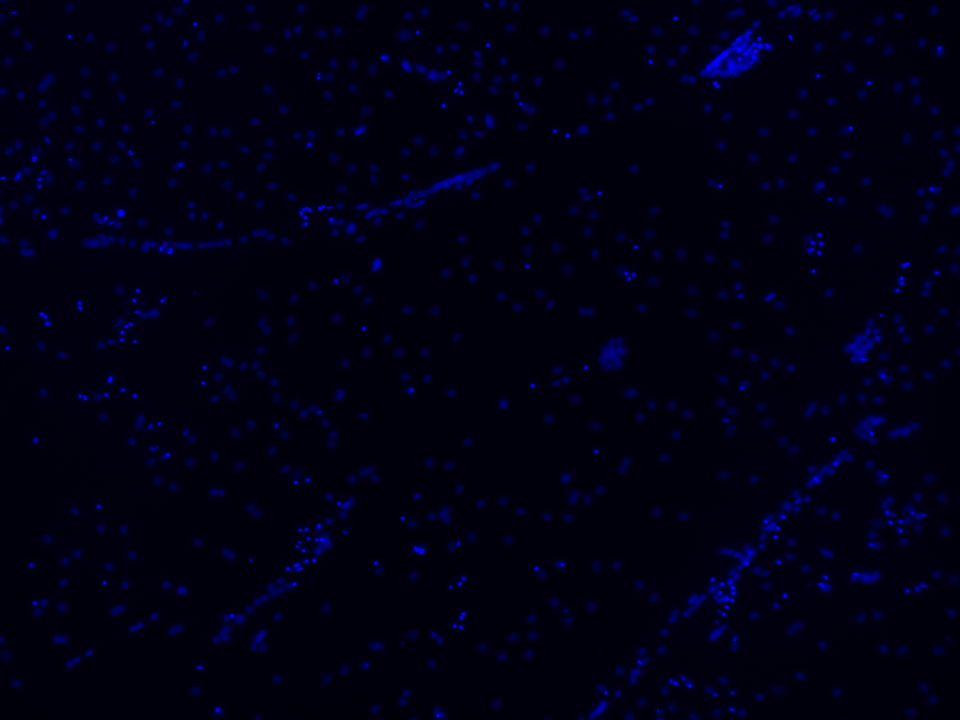

Supplement: Supplementary file 4 — Supplementary Data 1 [file 42003_2022_3423_MOESM4_ESM.zip › Figure S7 (Images)/Raw image files/10x BSC2 P2 Beefy-9 high FGF/XY05/image_XY05_CH1.tif]

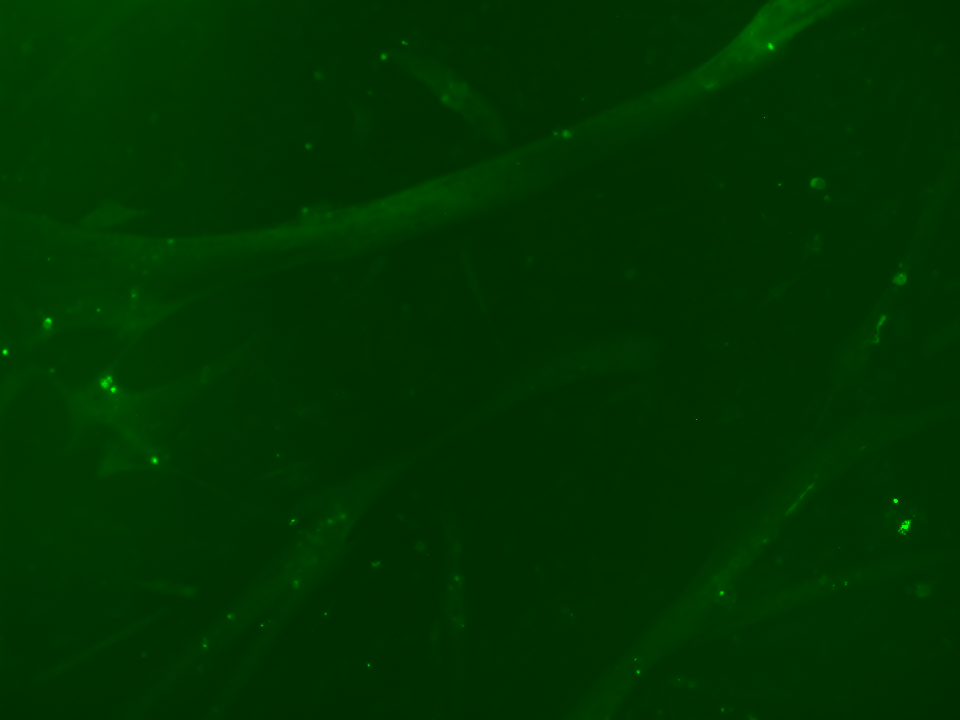

Supplement: Supplementary file 4 — Supplementary Data 1 [file 42003_2022_3423_MOESM4_ESM.zip › Figure S7 (Images)/Raw image files/10x BSC2 P2 Beefy-9 high FGF/XY05/image_XY05_CH2.tif]

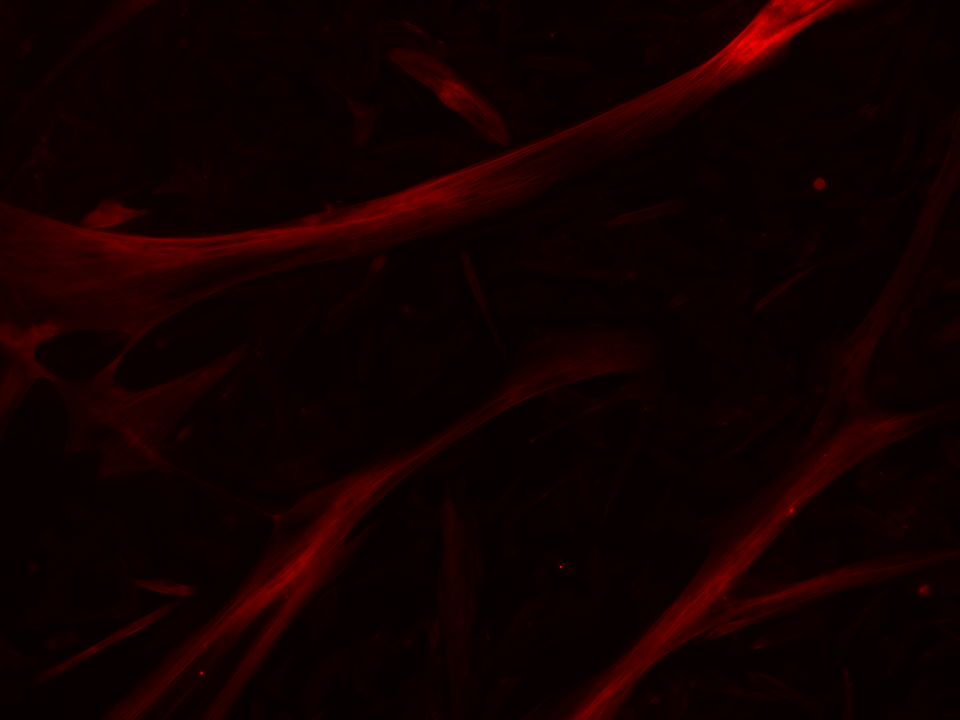

Supplement: Supplementary file 4 — Supplementary Data 1 [file 42003_2022_3423_MOESM4_ESM.zip › Figure S7 (Images)/Raw image files/10x BSC2 P2 Beefy-9 high FGF/XY05/image_XY05_CH3.tif]

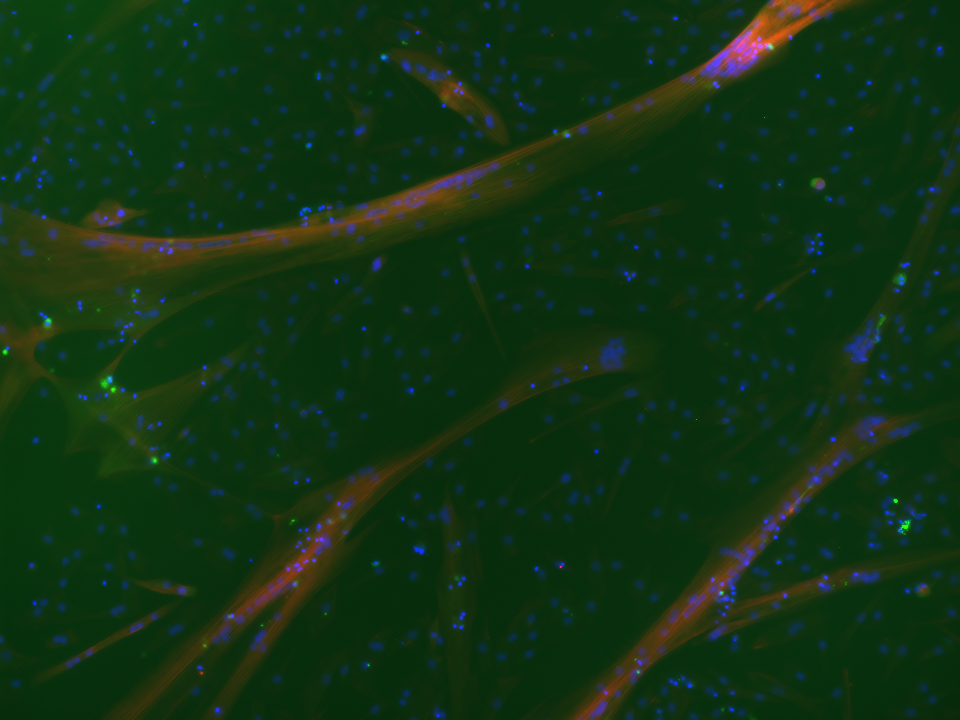

Supplement: Supplementary file 4 — Supplementary Data 1 [file 42003_2022_3423_MOESM4_ESM.zip › Figure S7 (Images)/Raw image files/10x BSC2 P2 Beefy-9 high FGF/XY05/image_XY05_Overlay.tif]

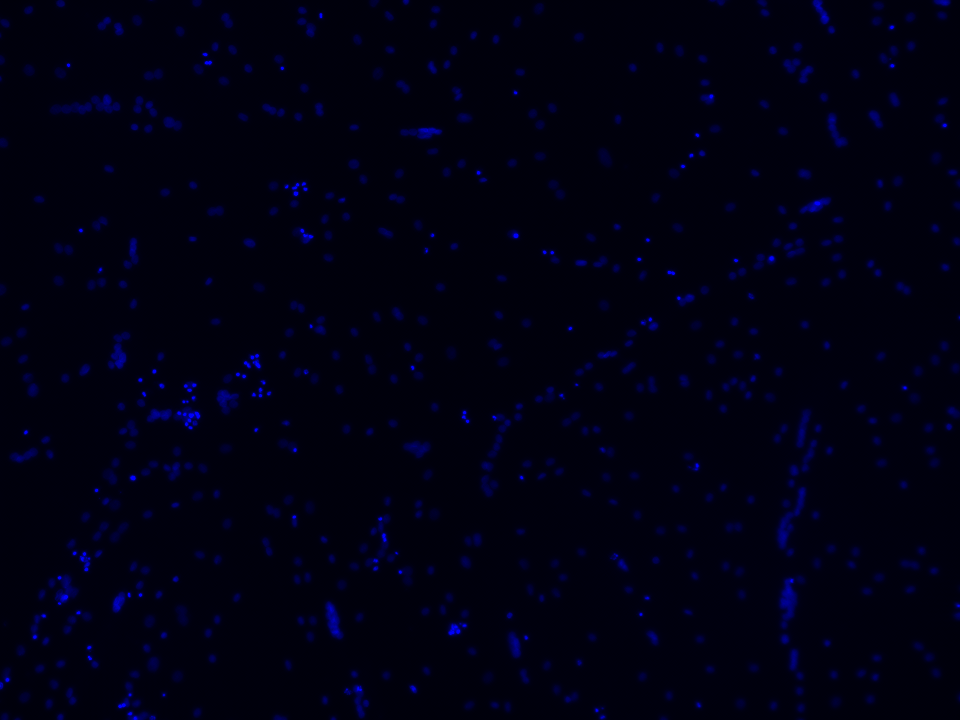

Supplement: Supplementary file 4 — Supplementary Data 1 [file 42003_2022_3423_MOESM4_ESM.zip › Figure S7 (Images)/Raw image files/10x BSC2 P2 Beefy-9 high FGF/XY06/image_XY06_CH1.tif]

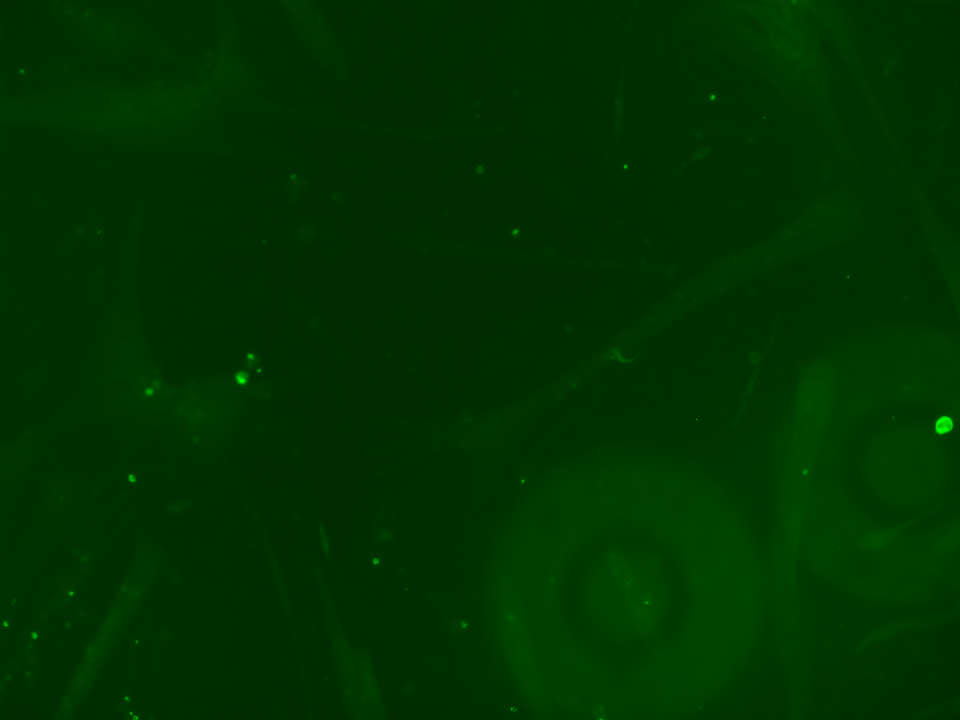

Supplement: Supplementary file 4 — Supplementary Data 1 [file 42003_2022_3423_MOESM4_ESM.zip › Figure S7 (Images)/Raw image files/10x BSC2 P2 Beefy-9 high FGF/XY06/image_XY06_CH2.tif]

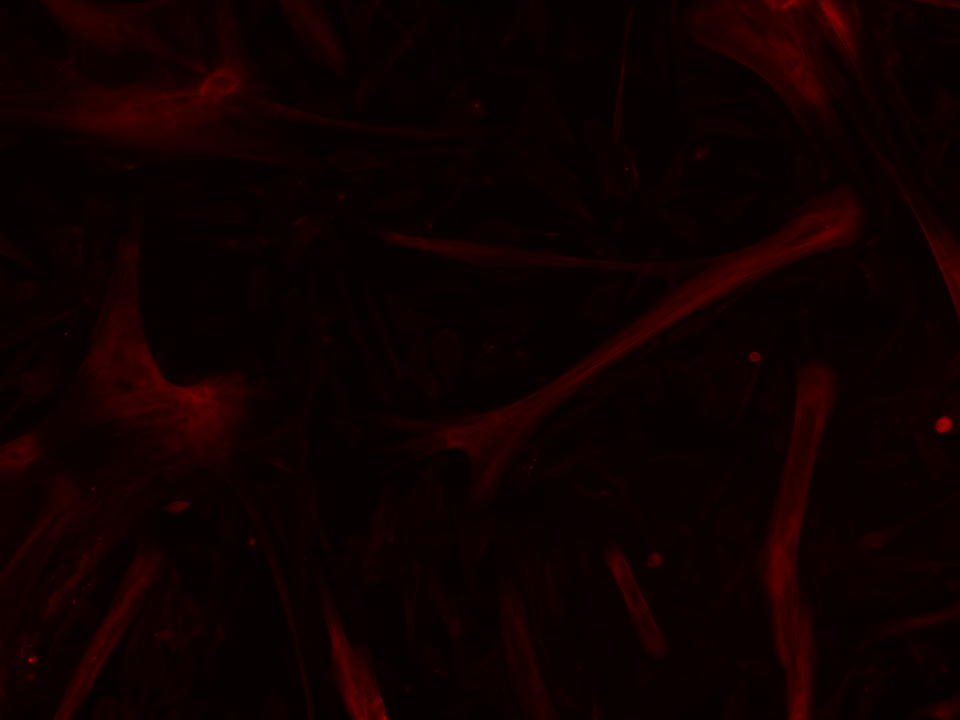

Supplement: Supplementary file 4 — Supplementary Data 1 [file 42003_2022_3423_MOESM4_ESM.zip › Figure S7 (Images)/Raw image files/10x BSC2 P2 Beefy-9 high FGF/XY06/image_XY06_CH3.tif]

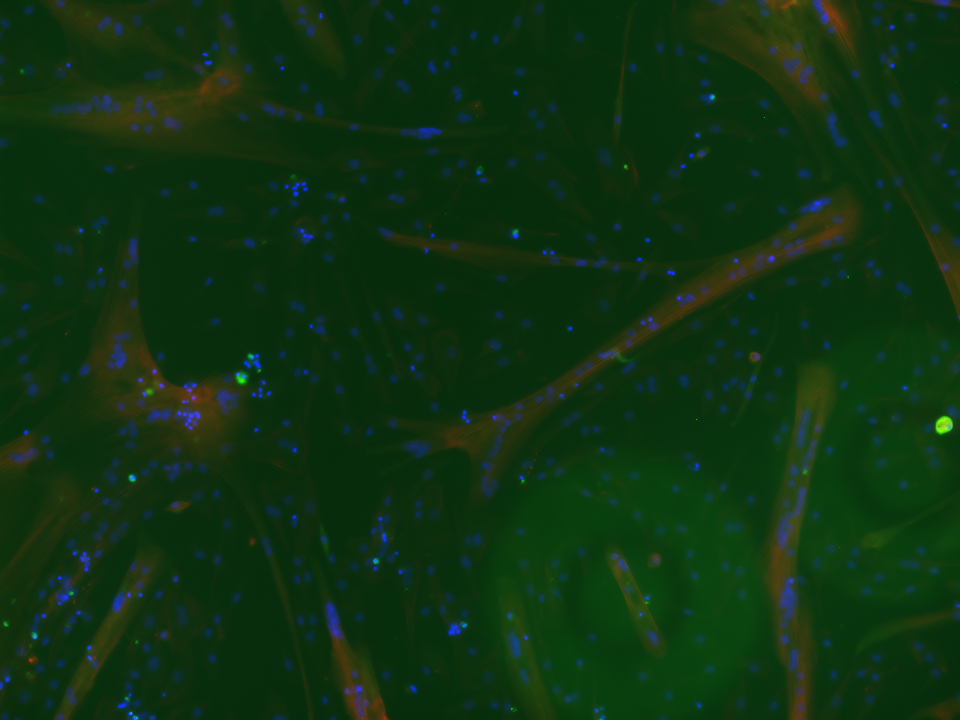

Supplement: Supplementary file 4 — Supplementary Data 1 [file 42003_2022_3423_MOESM4_ESM.zip › Figure S7 (Images)/Raw image files/10x BSC2 P2 Beefy-9 high FGF/XY06/image_XY06_Overlay.tif]

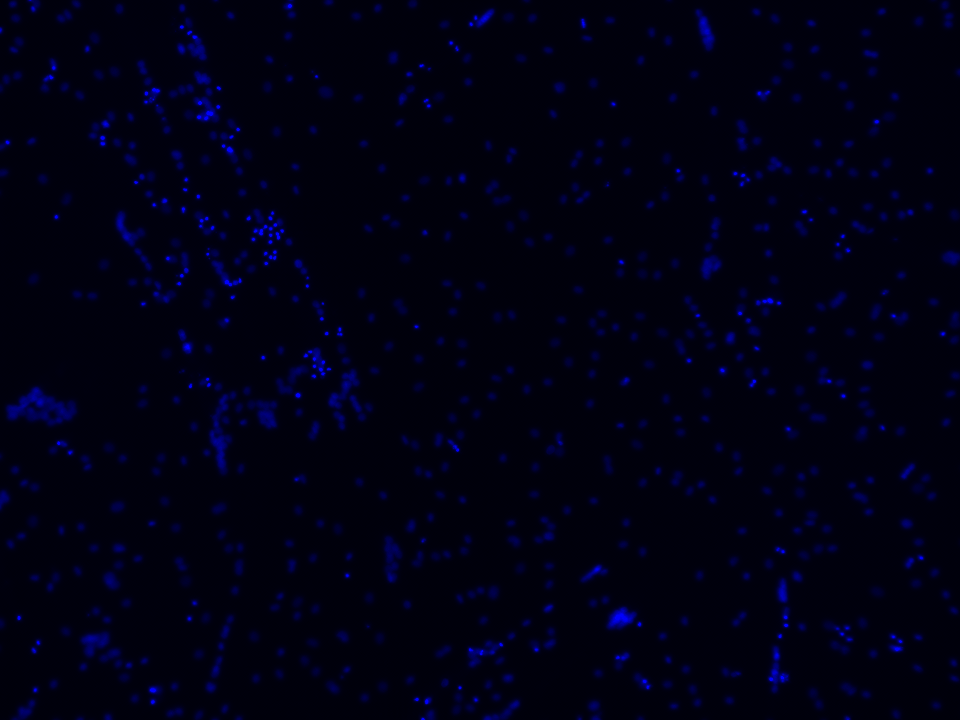

Supplement: Supplementary file 4 — Supplementary Data 1 [file 42003_2022_3423_MOESM4_ESM.zip › Figure S7 (Images)/Raw image files/10x BSC2 P2 Beefy-9 high FGF/XY07/image_XY07_CH1.tif]

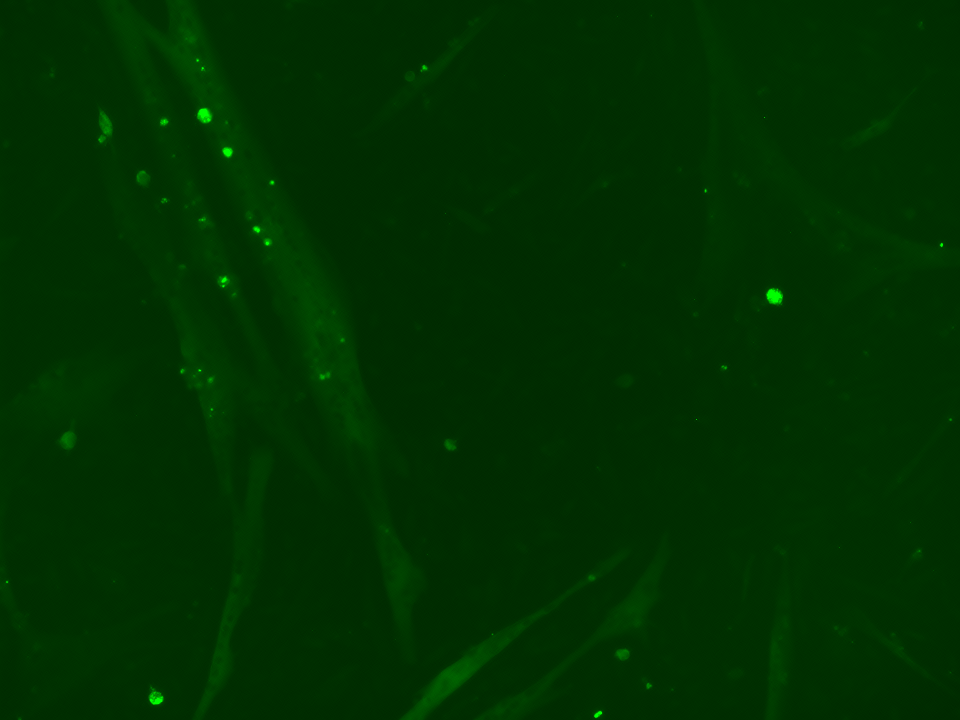

Supplement: Supplementary file 4 — Supplementary Data 1 [file 42003_2022_3423_MOESM4_ESM.zip › Figure S7 (Images)/Raw image files/10x BSC2 P2 Beefy-9 high FGF/XY07/image_XY07_CH2.tif]

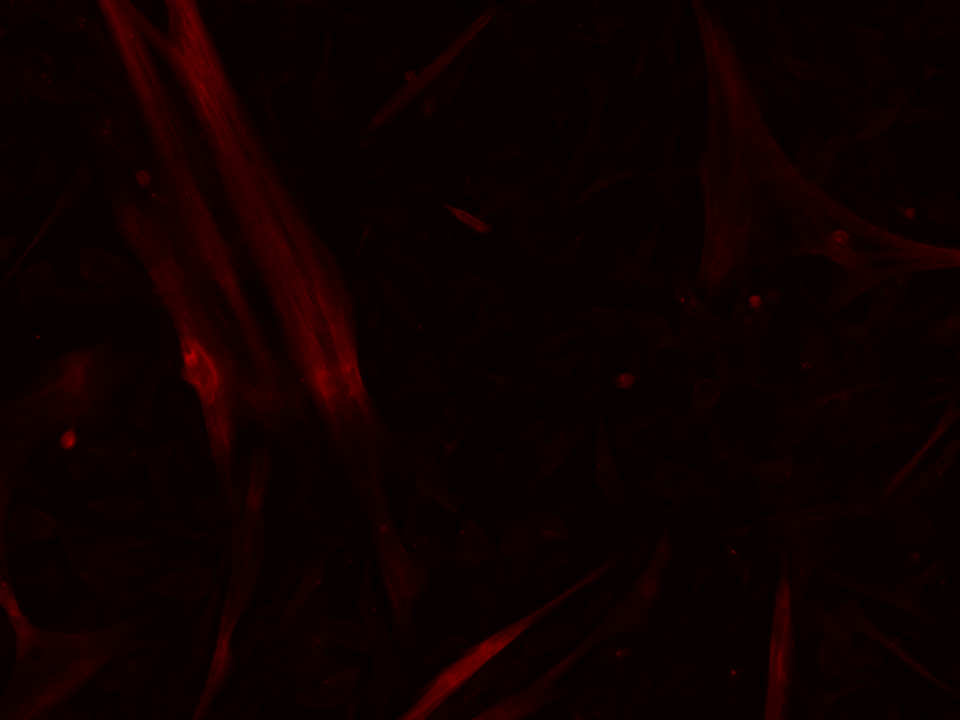

Supplement: Supplementary file 4 — Supplementary Data 1 [file 42003_2022_3423_MOESM4_ESM.zip › Figure S7 (Images)/Raw image files/10x BSC2 P2 Beefy-9 high FGF/XY07/image_XY07_CH3.tif]

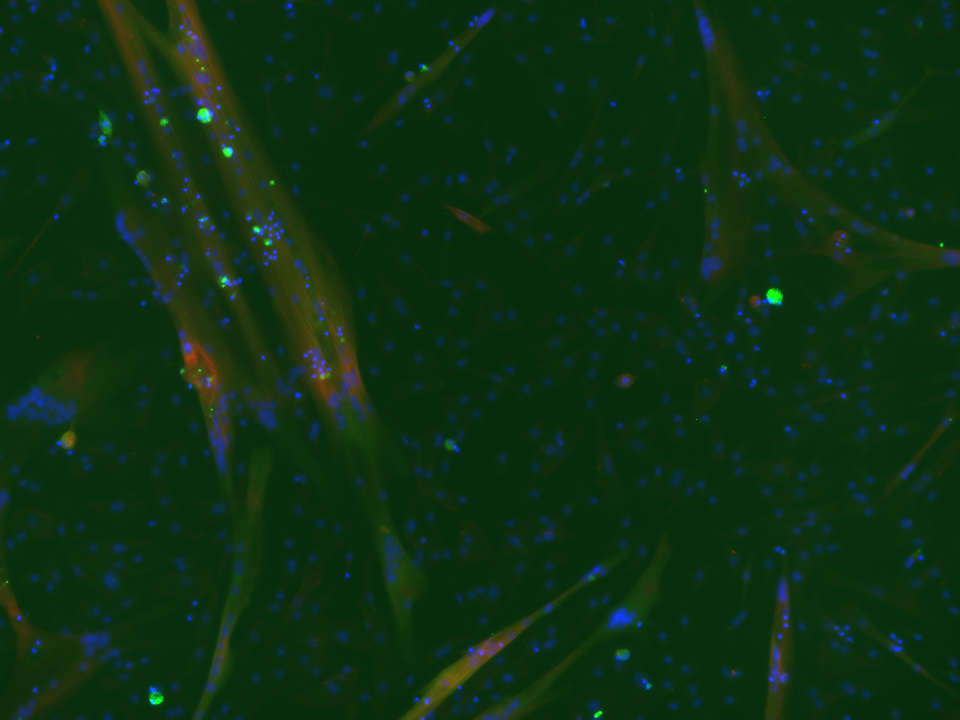

Supplement: Supplementary file 4 — Supplementary Data 1 [file 42003_2022_3423_MOESM4_ESM.zip › Figure S7 (Images)/Raw image files/10x BSC2 P2 Beefy-9 high FGF/XY07/image_XY07_Overlay.tif]

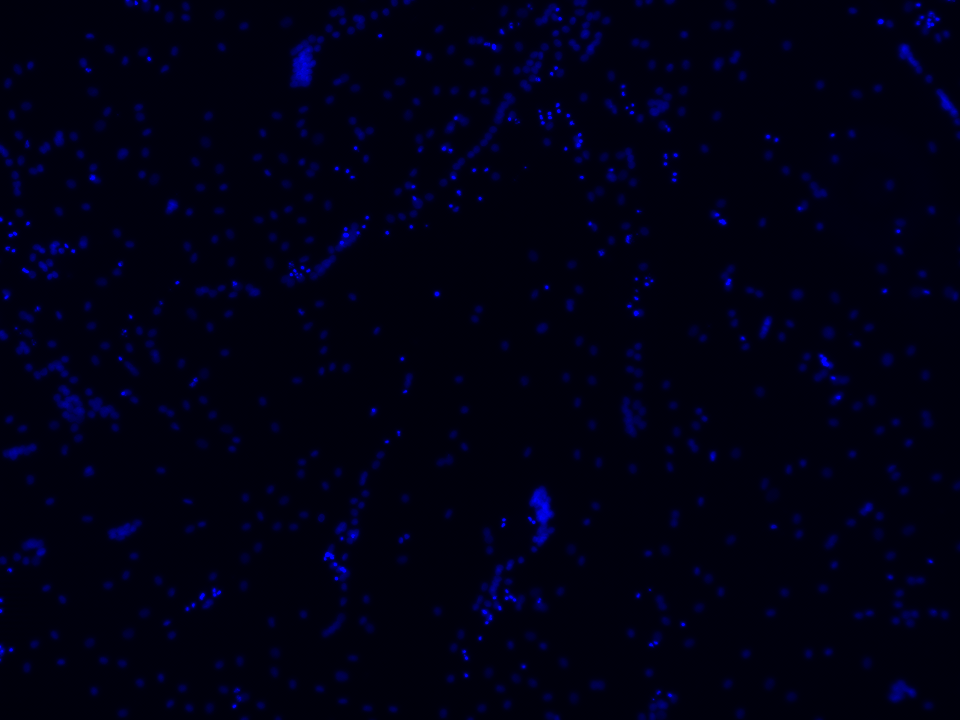

Supplement: Supplementary file 4 — Supplementary Data 1 [file 42003_2022_3423_MOESM4_ESM.zip › Figure S7 (Images)/Raw image files/10x BSC2 P2 Beefy-9 high FGF/XY08/image_XY08_CH1.tif]

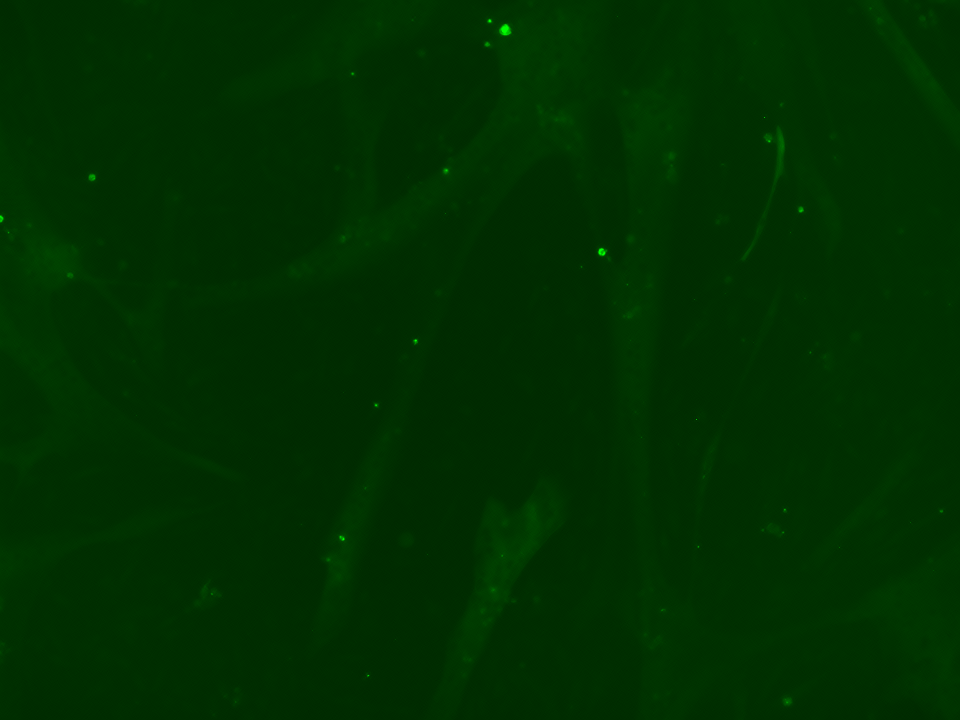

Supplement: Supplementary file 4 — Supplementary Data 1 [file 42003_2022_3423_MOESM4_ESM.zip › Figure S7 (Images)/Raw image files/10x BSC2 P2 Beefy-9 high FGF/XY08/image_XY08_CH2.tif]

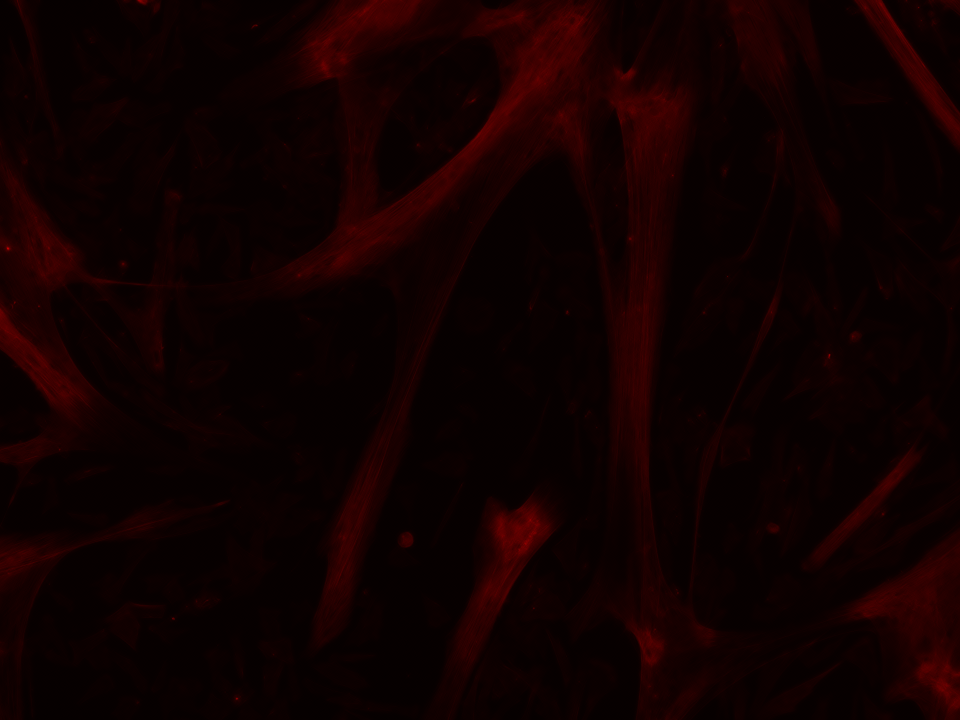

Supplement: Supplementary file 4 — Supplementary Data 1 [file 42003_2022_3423_MOESM4_ESM.zip › Figure S7 (Images)/Raw image files/10x BSC2 P2 Beefy-9 high FGF/XY08/image_XY08_CH3.tif]

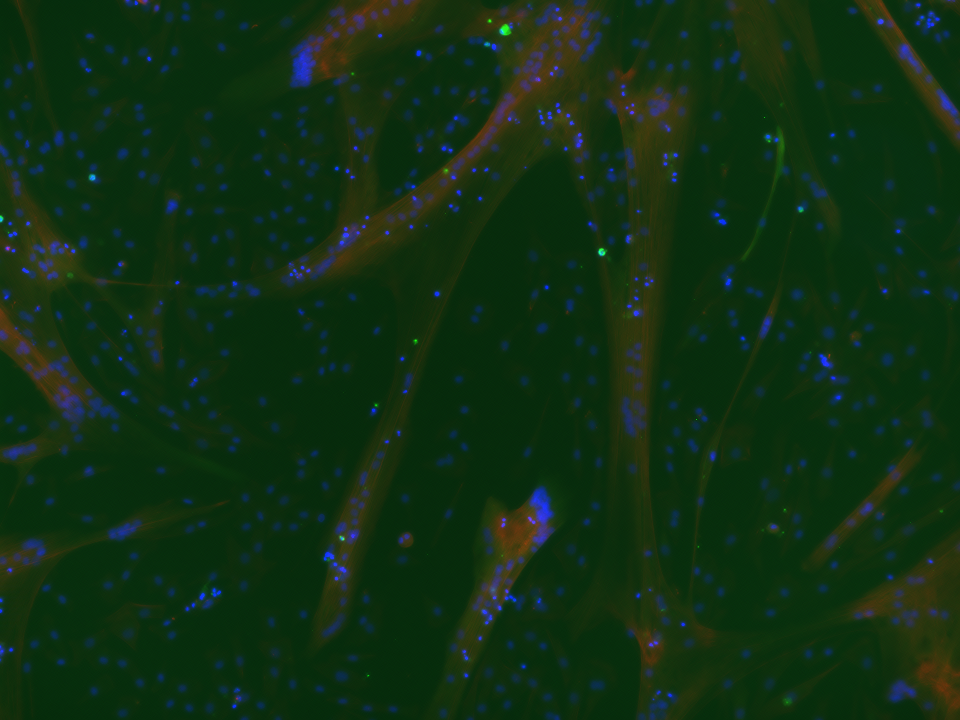

Supplement: Supplementary file 4 — Supplementary Data 1 [file 42003_2022_3423_MOESM4_ESM.zip › Figure S7 (Images)/Raw image files/10x BSC2 P2 Beefy-9 high FGF/XY08/image_XY08_Overlay.tif]

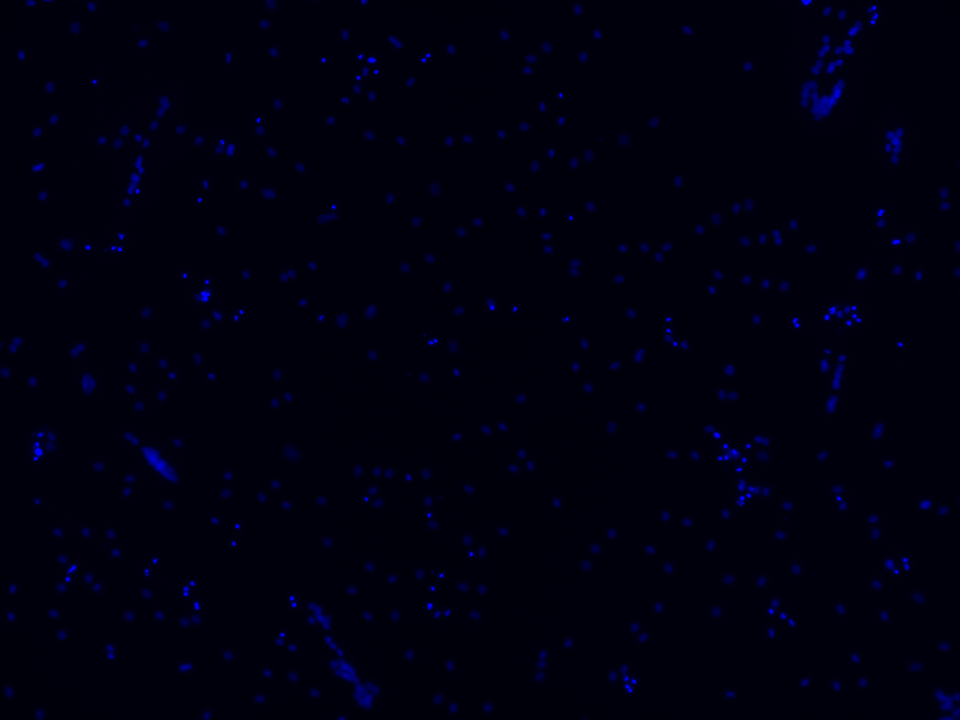

Supplement: Supplementary file 4 — Supplementary Data 1 [file 42003_2022_3423_MOESM4_ESM.zip › Figure S7 (Images)/Raw image files/10x BSC2 P2 Beefy-9 low FGF/XY01/image_XY01_CH1.tif]

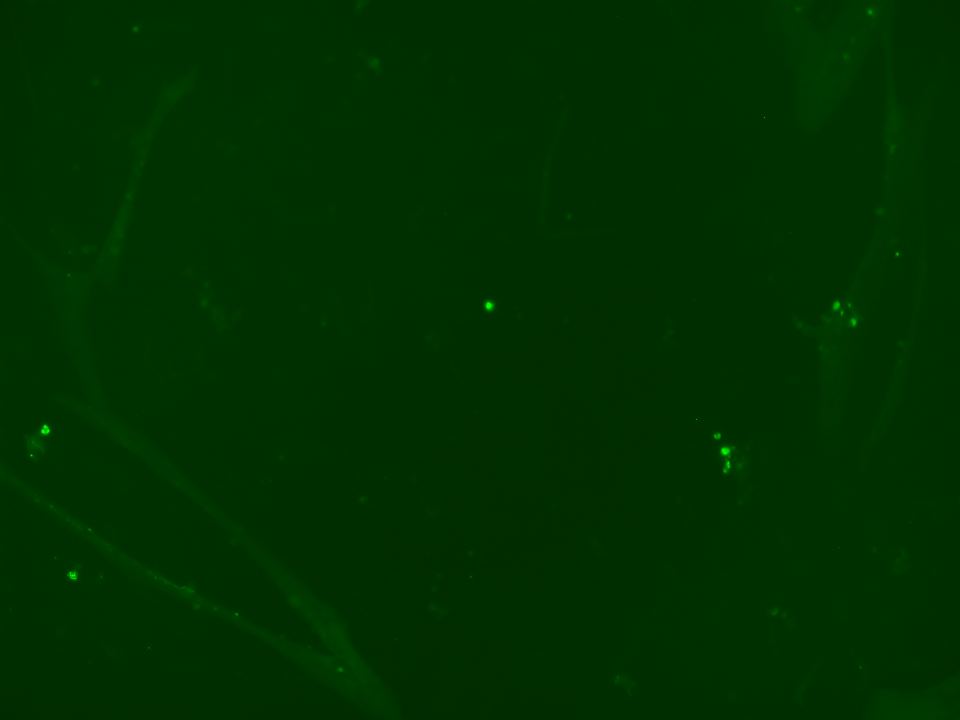

Supplement: Supplementary file 4 — Supplementary Data 1 [file 42003_2022_3423_MOESM4_ESM.zip › Figure S7 (Images)/Raw image files/10x BSC2 P2 Beefy-9 low FGF/XY01/image_XY01_CH2.tif]

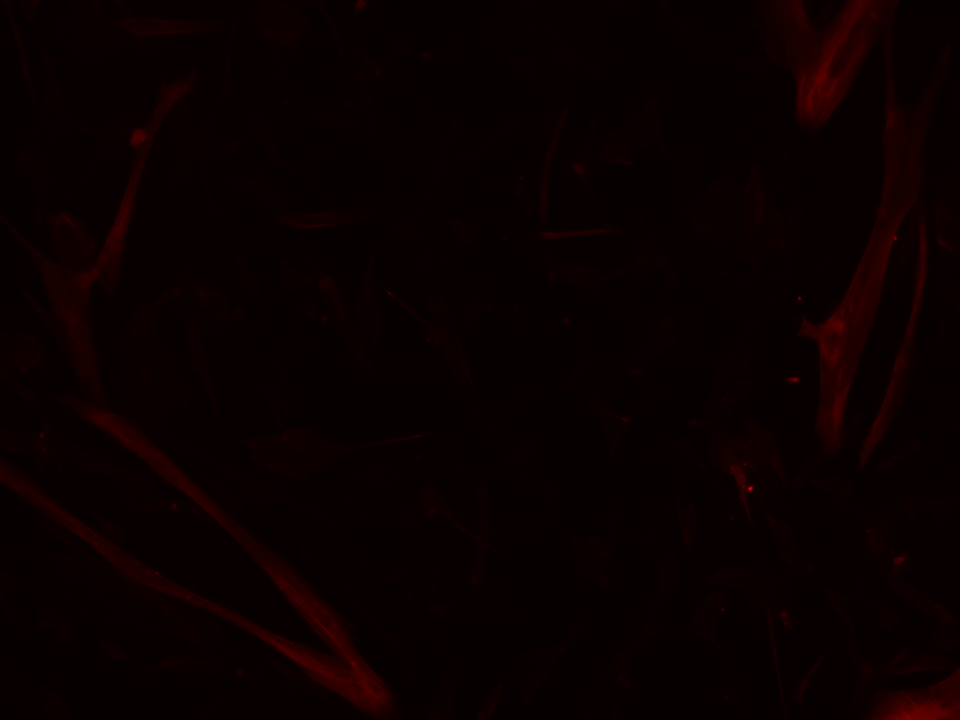

Supplement: Supplementary file 4 — Supplementary Data 1 [file 42003_2022_3423_MOESM4_ESM.zip › Figure S7 (Images)/Raw image files/10x BSC2 P2 Beefy-9 low FGF/XY01/image_XY01_CH3.tif]

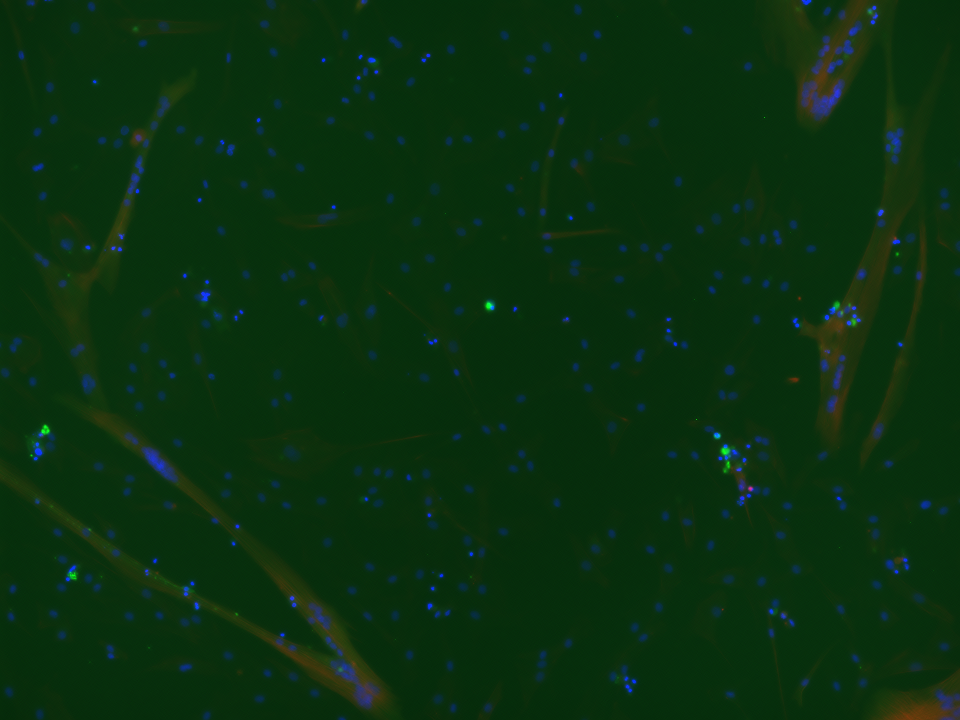

Supplement: Supplementary file 4 — Supplementary Data 1 [file 42003_2022_3423_MOESM4_ESM.zip › Figure S7 (Images)/Raw image files/10x BSC2 P2 Beefy-9 low FGF/XY01/image_XY01_Overlay.tif]

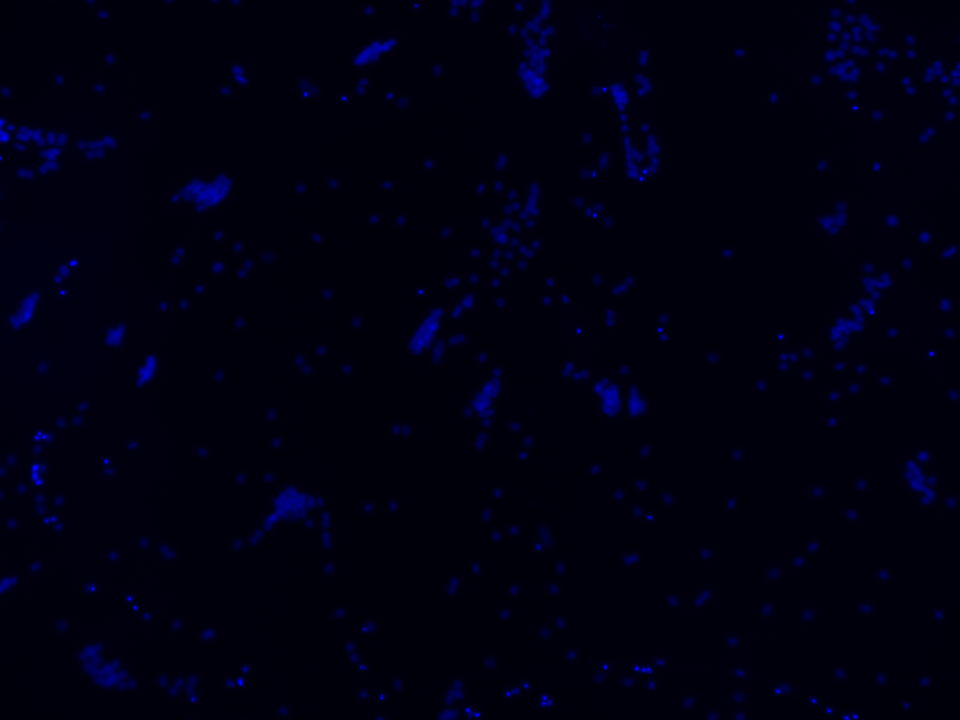

Supplement: Supplementary file 4 — Supplementary Data 1 [file 42003_2022_3423_MOESM4_ESM.zip › Figure S7 (Images)/Raw image files/10x BSC2 P2 Beefy-9 low FGF/XY02/image_XY02_CH1.tif]

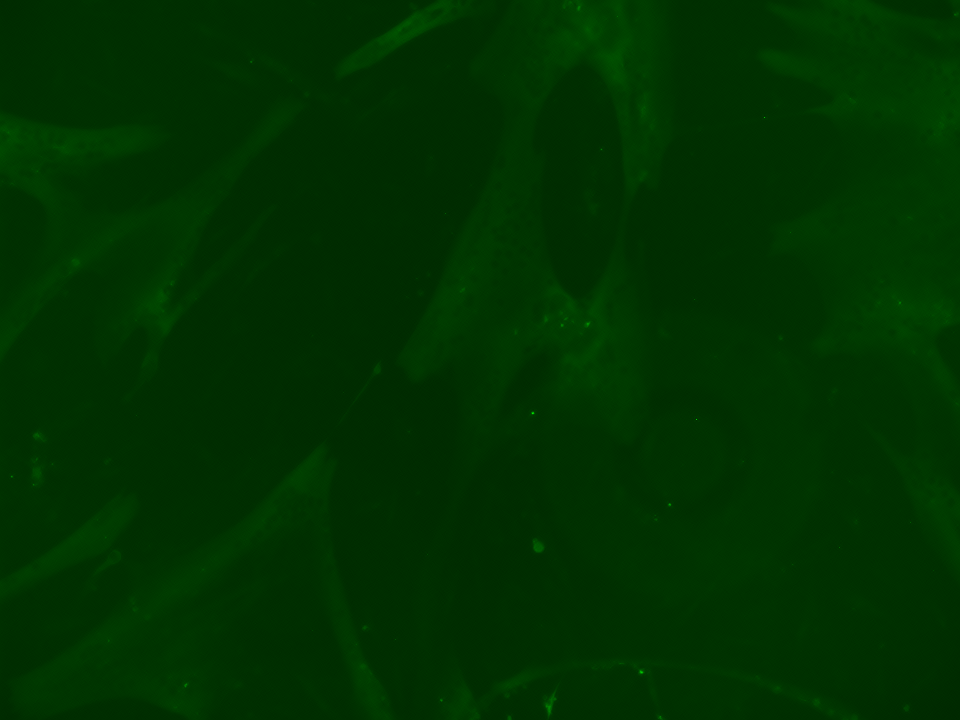

Supplement: Supplementary file 4 — Supplementary Data 1 [file 42003_2022_3423_MOESM4_ESM.zip › Figure S7 (Images)/Raw image files/10x BSC2 P2 Beefy-9 low FGF/XY02/image_XY02_CH2.tif]

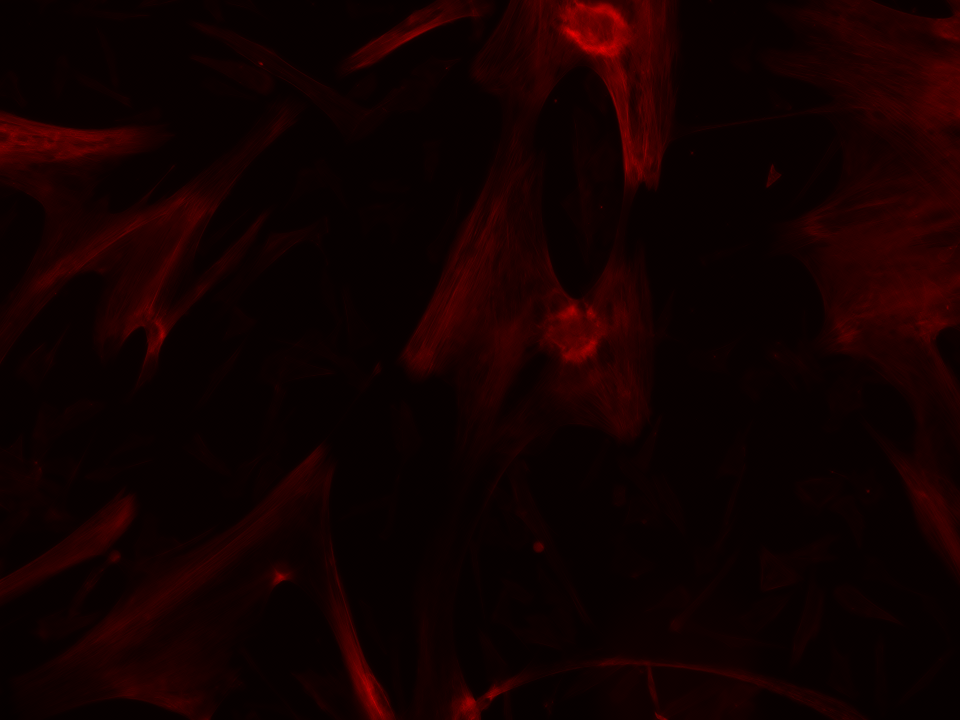

Supplement: Supplementary file 4 — Supplementary Data 1 [file 42003_2022_3423_MOESM4_ESM.zip › Figure S7 (Images)/Raw image files/10x BSC2 P2 Beefy-9 low FGF/XY02/image_XY02_CH3.tif]

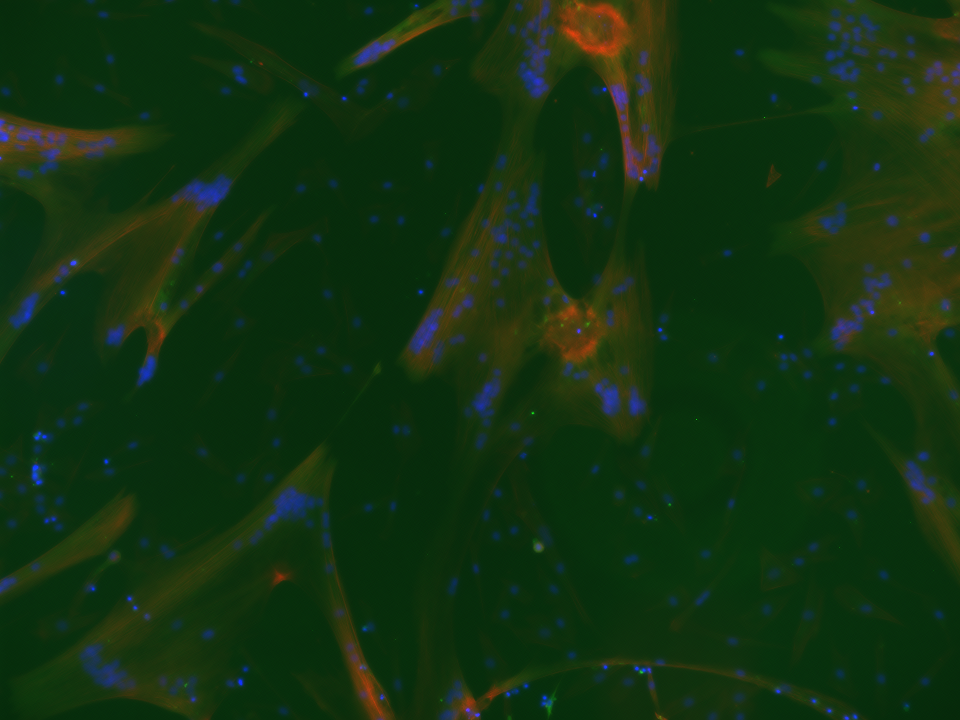

Supplement: Supplementary file 4 — Supplementary Data 1 [file 42003_2022_3423_MOESM4_ESM.zip › Figure S7 (Images)/Raw image files/10x BSC2 P2 Beefy-9 low FGF/XY02/image_XY02_Overlay.tif]

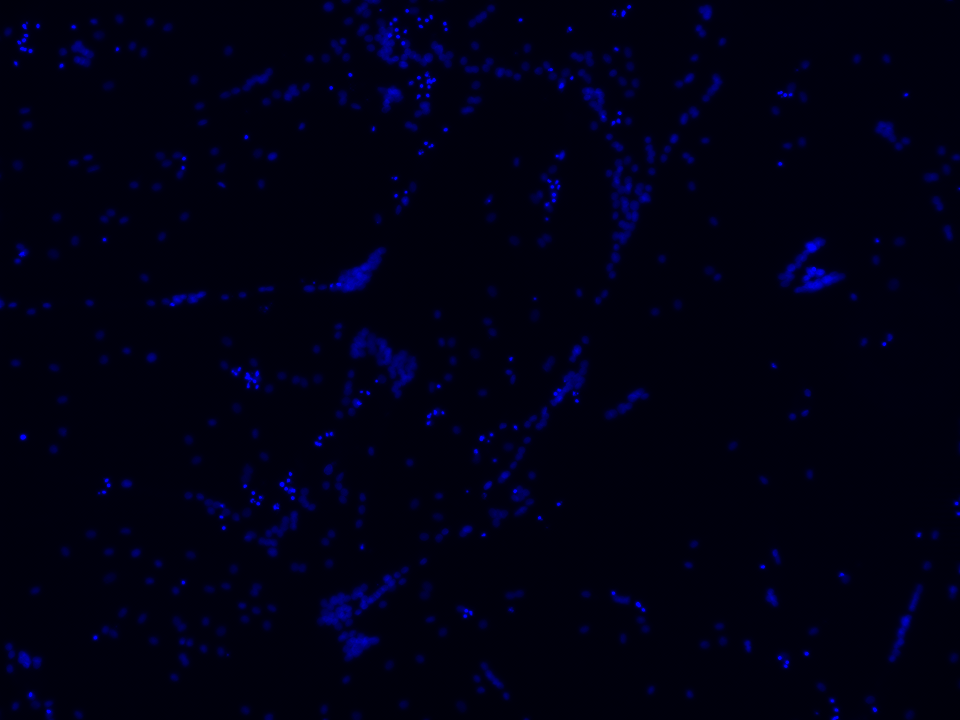

Supplement: Supplementary file 4 — Supplementary Data 1 [file 42003_2022_3423_MOESM4_ESM.zip › Figure S7 (Images)/Raw image files/10x BSC2 P2 Beefy-9 low FGF/XY03/image_XY03_CH1.tif]

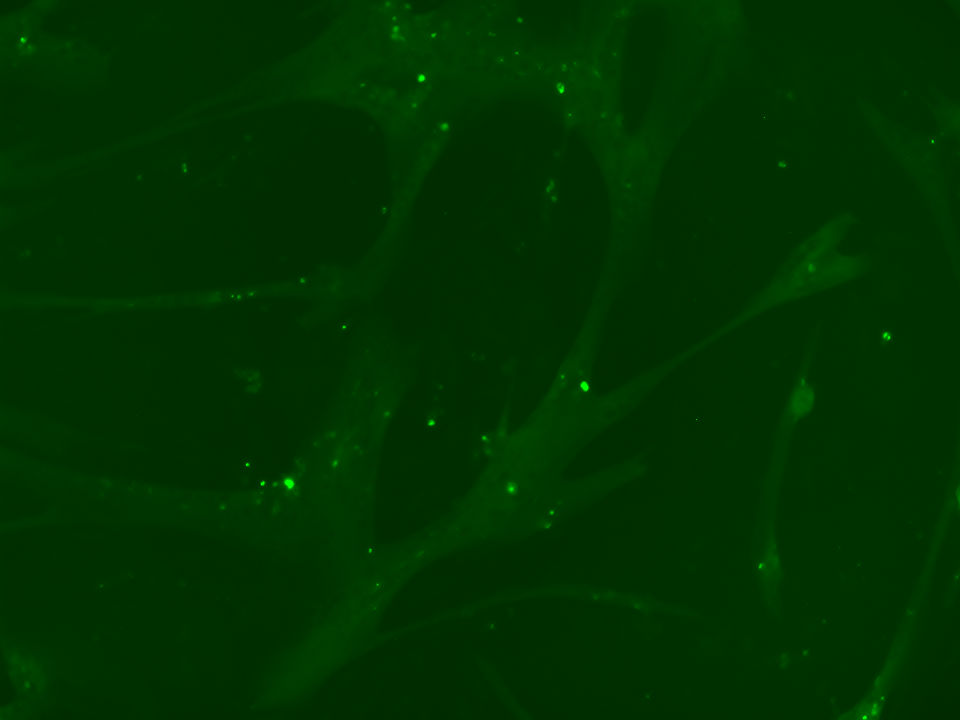

Supplement: Supplementary file 4 — Supplementary Data 1 [file 42003_2022_3423_MOESM4_ESM.zip › Figure S7 (Images)/Raw image files/10x BSC2 P2 Beefy-9 low FGF/XY03/image_XY03_CH2.tif]

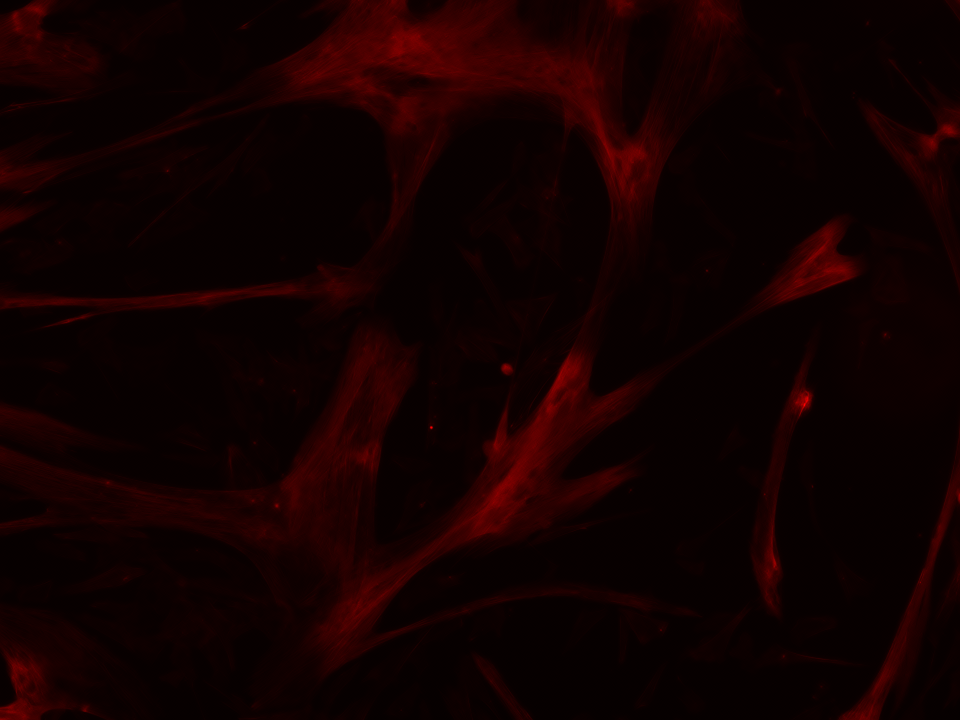

Supplement: Supplementary file 4 — Supplementary Data 1 [file 42003_2022_3423_MOESM4_ESM.zip › Figure S7 (Images)/Raw image files/10x BSC2 P2 Beefy-9 low FGF/XY03/image_XY03_CH3.tif]

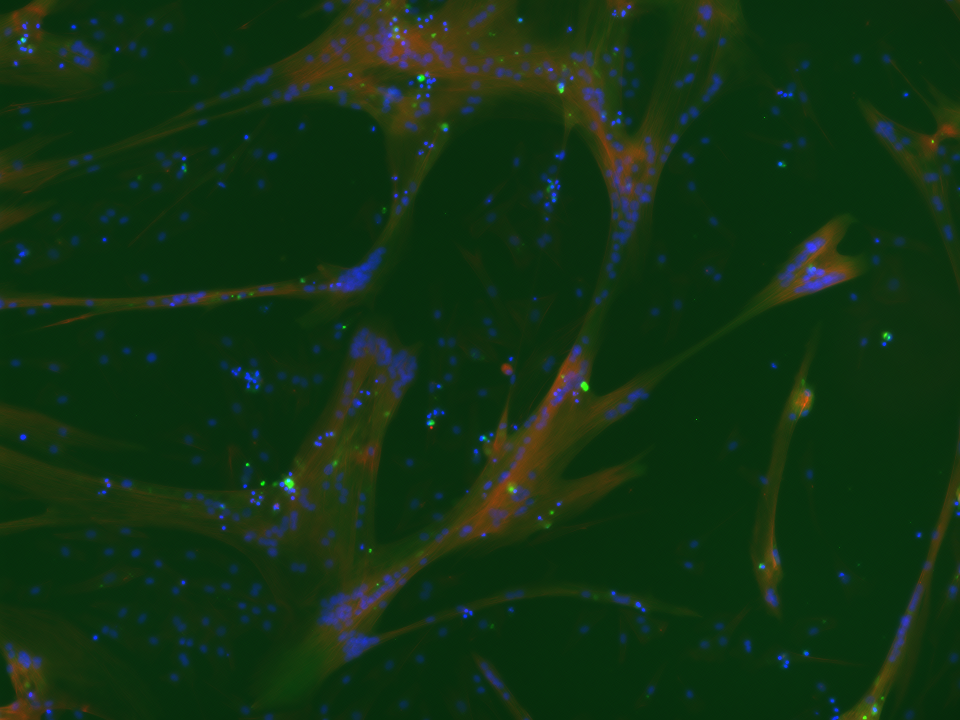

Supplement: Supplementary file 4 — Supplementary Data 1 [file 42003_2022_3423_MOESM4_ESM.zip › Figure S7 (Images)/Raw image files/10x BSC2 P2 Beefy-9 low FGF/XY03/image_XY03_Overlay.tif]

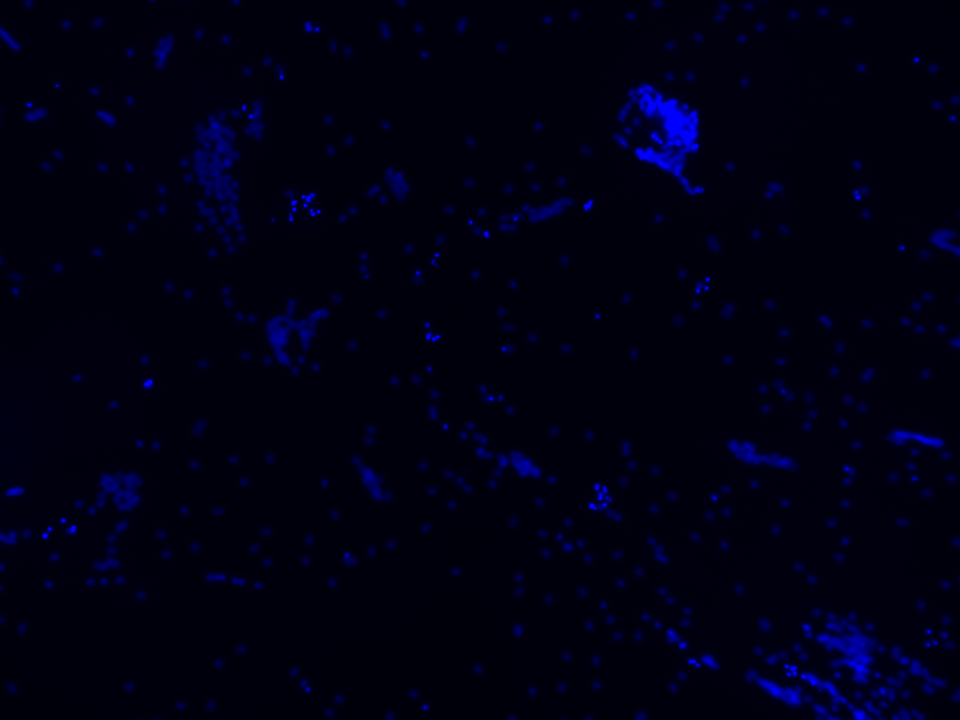

Supplement: Supplementary file 4 — Supplementary Data 1 [file 42003_2022_3423_MOESM4_ESM.zip › Figure S7 (Images)/Raw image files/10x BSC2 P2 Beefy-9 low FGF/XY04/image_XY04_CH1.tif]

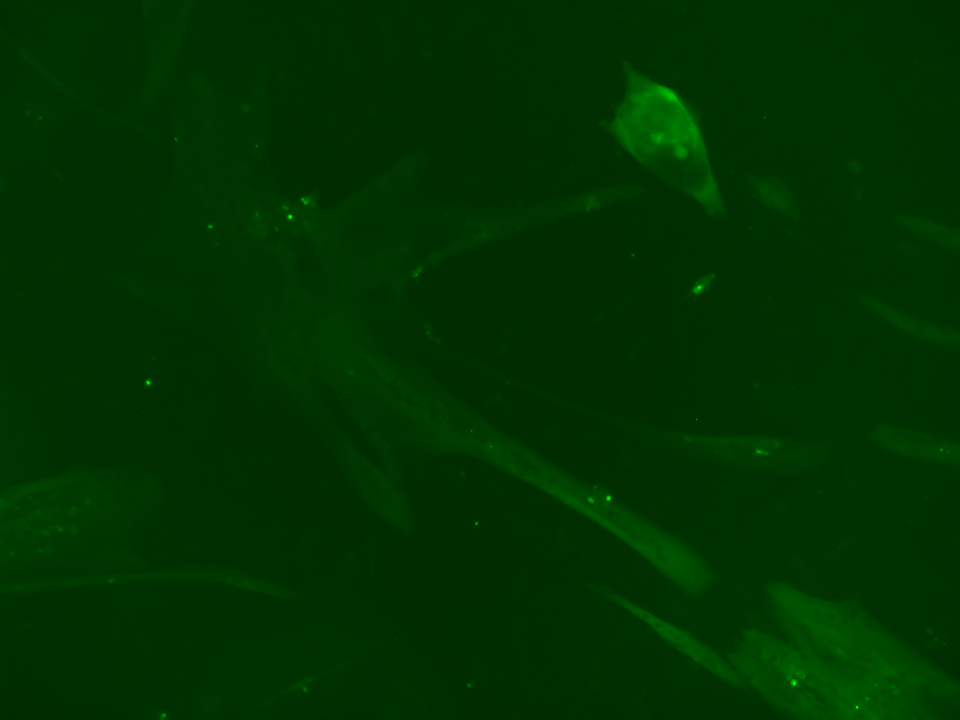

Supplement: Supplementary file 4 — Supplementary Data 1 [file 42003_2022_3423_MOESM4_ESM.zip › Figure S7 (Images)/Raw image files/10x BSC2 P2 Beefy-9 low FGF/XY04/image_XY04_CH2.tif]

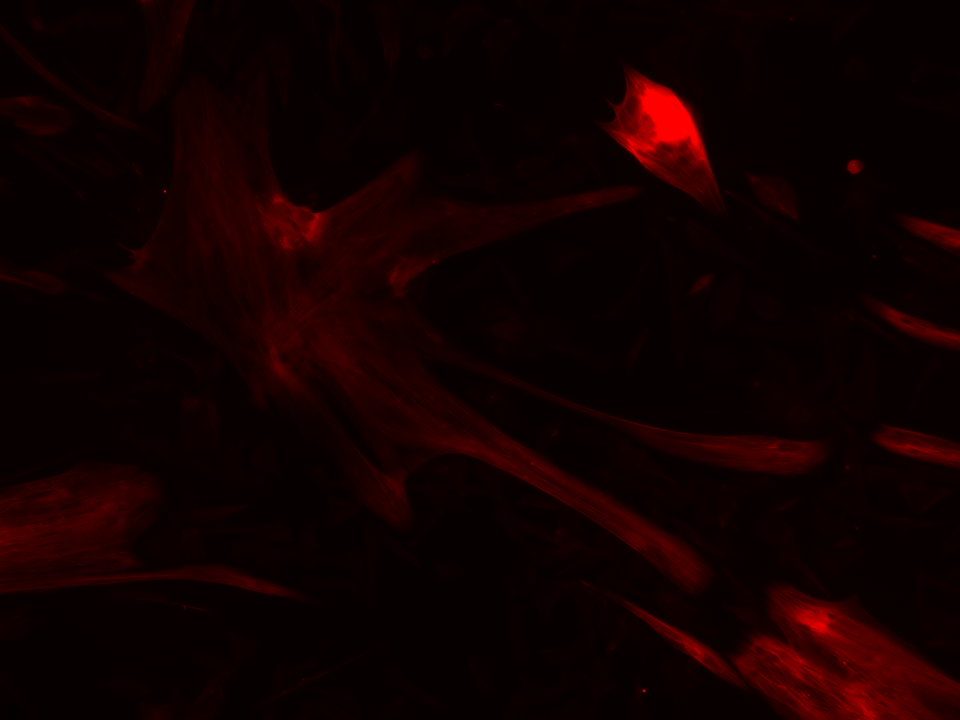

Supplement: Supplementary file 4 — Supplementary Data 1 [file 42003_2022_3423_MOESM4_ESM.zip › Figure S7 (Images)/Raw image files/10x BSC2 P2 Beefy-9 low FGF/XY04/image_XY04_CH3.tif]

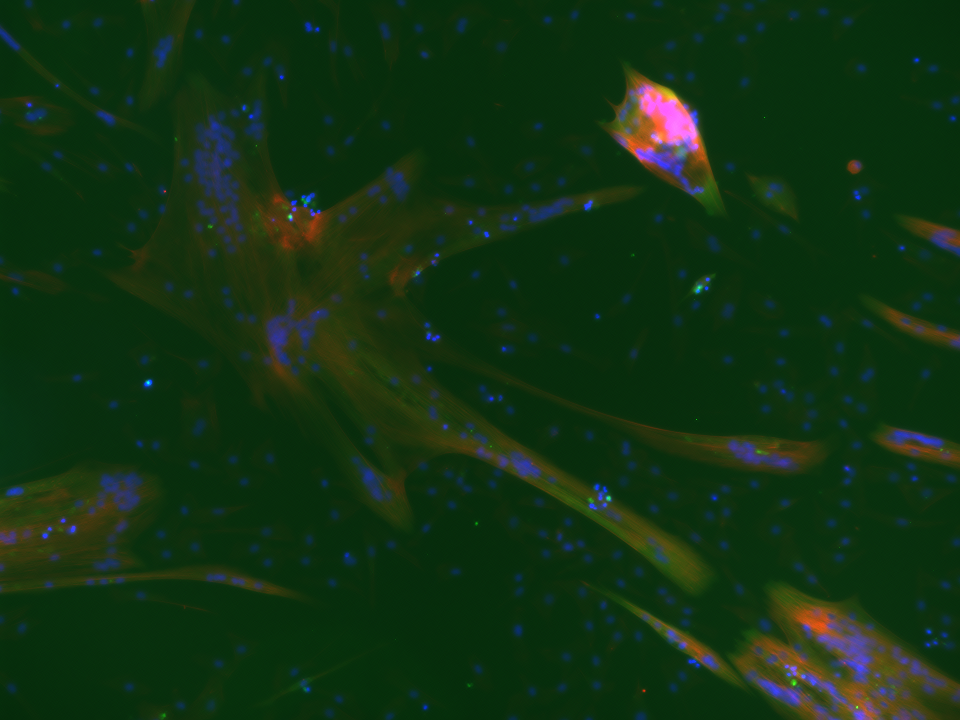

Supplement: Supplementary file 4 — Supplementary Data 1 [file 42003_2022_3423_MOESM4_ESM.zip › Figure S7 (Images)/Raw image files/10x BSC2 P2 Beefy-9 low FGF/XY04/image_XY04_Overlay.tif]

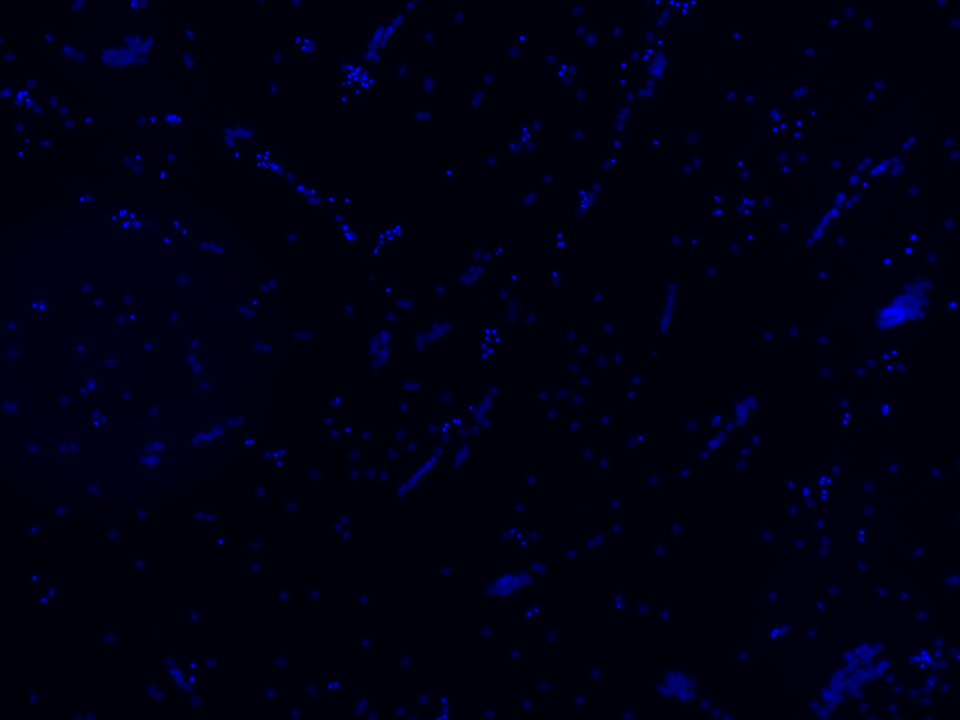

Supplement: Supplementary file 4 — Supplementary Data 1 [file 42003_2022_3423_MOESM4_ESM.zip › Figure S7 (Images)/Raw image files/10x BSC2 P2 Beefy-9 low FGF/XY05/image_XY05_CH1.tif]

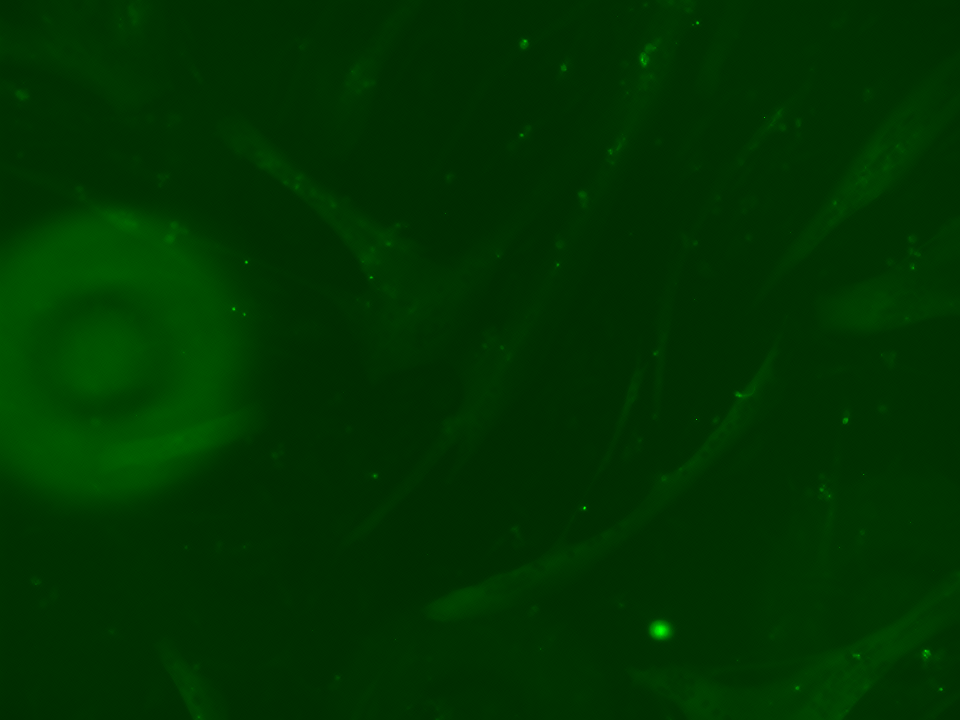

Supplement: Supplementary file 4 — Supplementary Data 1 [file 42003_2022_3423_MOESM4_ESM.zip › Figure S7 (Images)/Raw image files/10x BSC2 P2 Beefy-9 low FGF/XY05/image_XY05_CH2.tif]

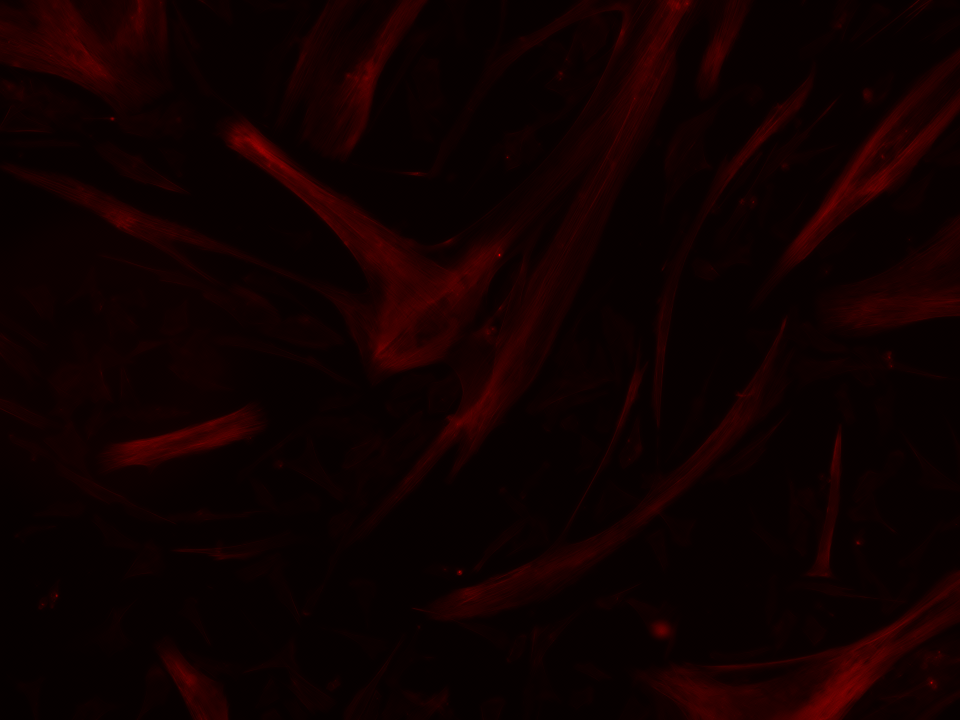

Supplement: Supplementary file 4 — Supplementary Data 1 [file 42003_2022_3423_MOESM4_ESM.zip › Figure S7 (Images)/Raw image files/10x BSC2 P2 Beefy-9 low FGF/XY05/image_XY05_CH3.tif]

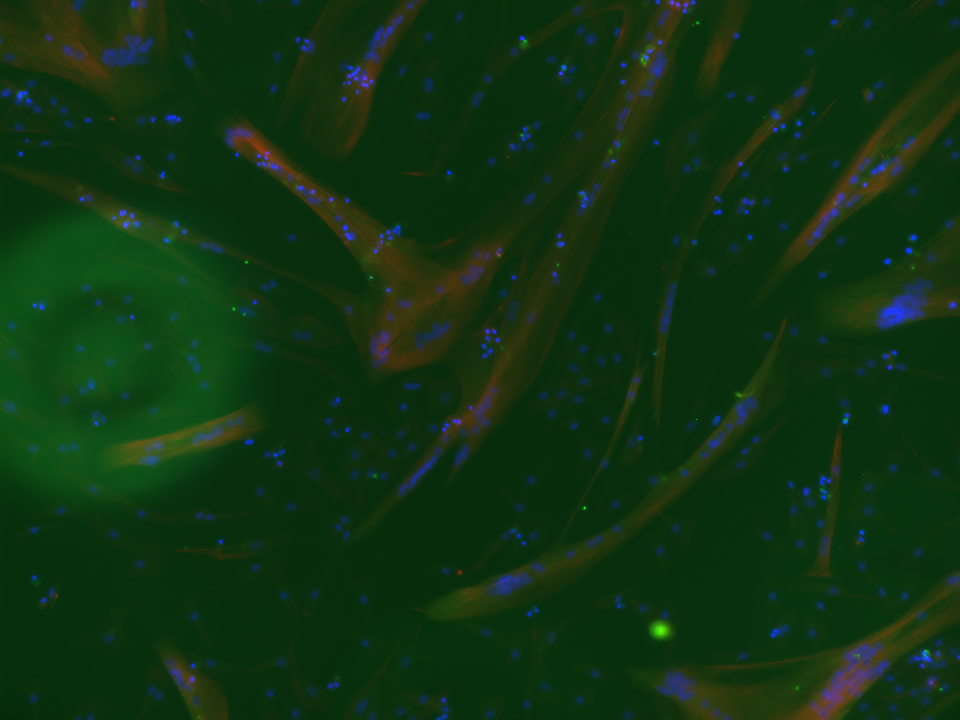

Supplement: Supplementary file 4 — Supplementary Data 1 [file 42003_2022_3423_MOESM4_ESM.zip › Figure S7 (Images)/Raw image files/10x BSC2 P2 Beefy-9 low FGF/XY05/image_XY05_Overlay.tif]

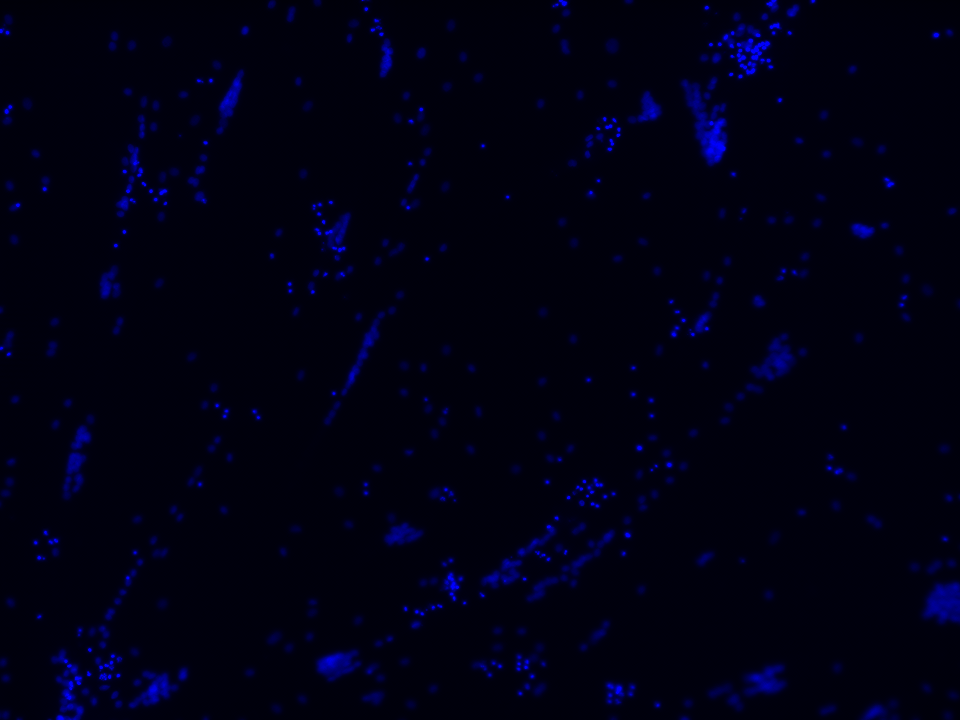

Supplement: Supplementary file 4 — Supplementary Data 1 [file 42003_2022_3423_MOESM4_ESM.zip › Figure S7 (Images)/Raw image files/10x BSC2 P2 Beefy-9 low FGF/XY06/image_XY06_CH1.tif]

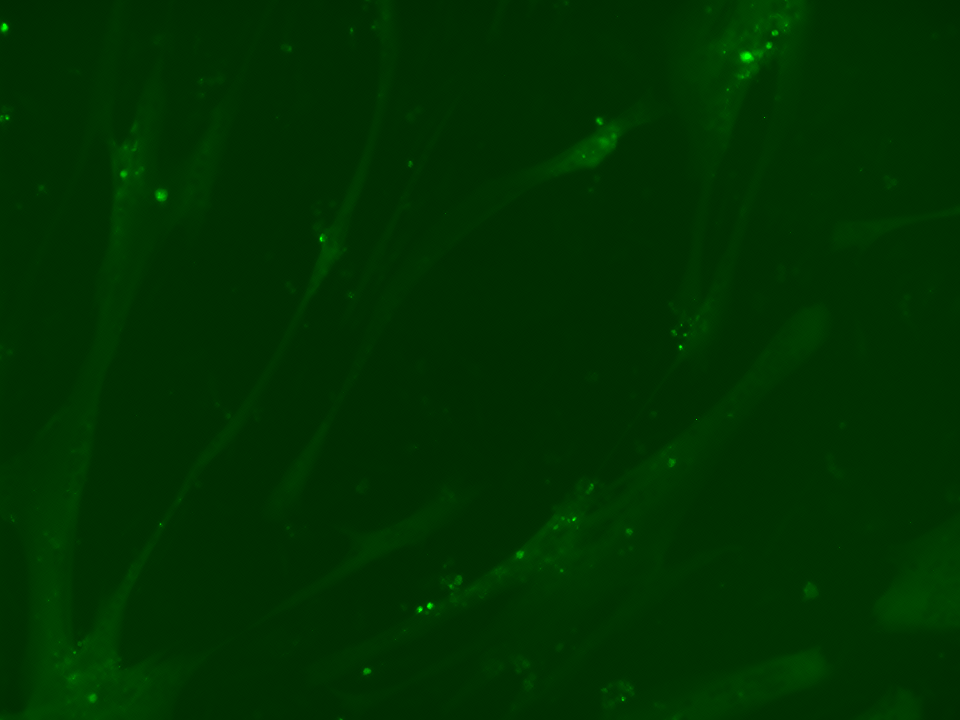

Supplement: Supplementary file 4 — Supplementary Data 1 [file 42003_2022_3423_MOESM4_ESM.zip › Figure S7 (Images)/Raw image files/10x BSC2 P2 Beefy-9 low FGF/XY06/image_XY06_CH2.tif]

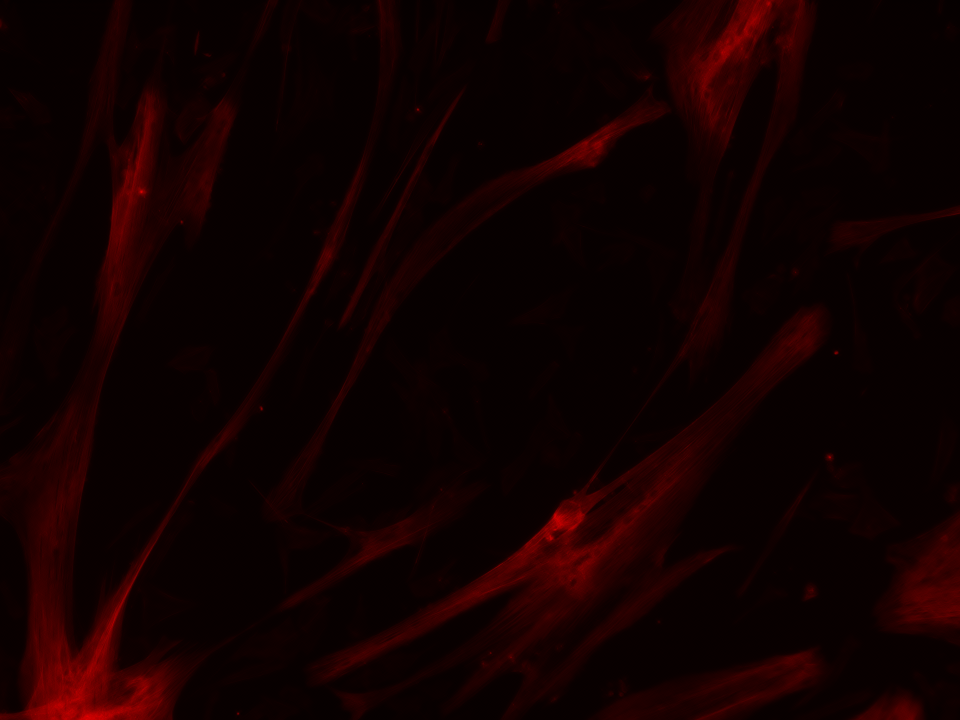

Supplement: Supplementary file 4 — Supplementary Data 1 [file 42003_2022_3423_MOESM4_ESM.zip › Figure S7 (Images)/Raw image files/10x BSC2 P2 Beefy-9 low FGF/XY06/image_XY06_CH3.tif]

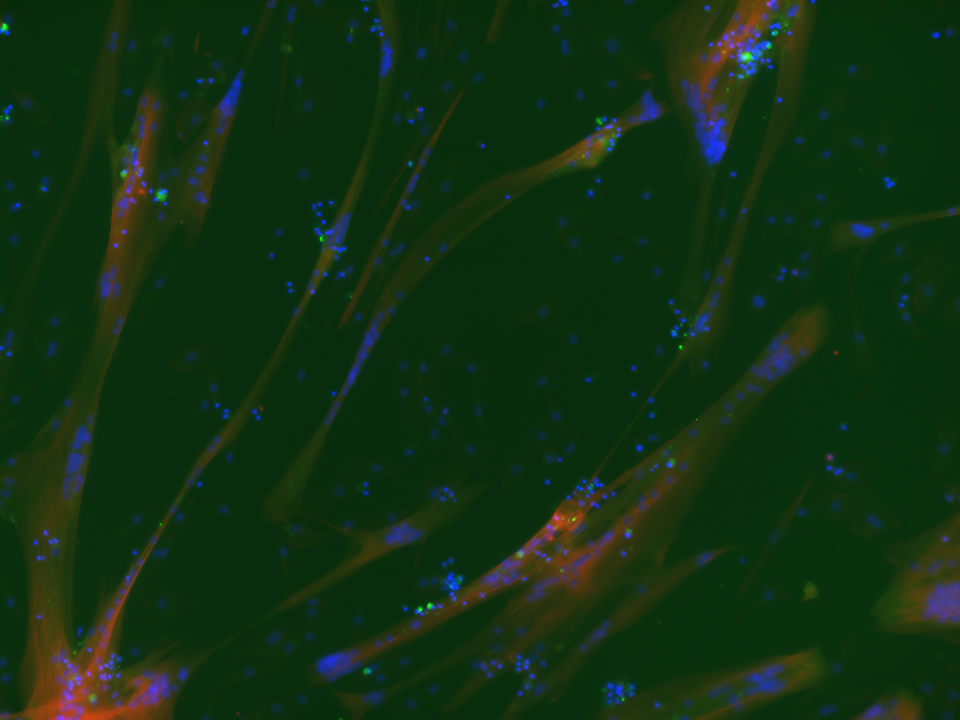

Supplement: Supplementary file 4 — Supplementary Data 1 [file 42003_2022_3423_MOESM4_ESM.zip › Figure S7 (Images)/Raw image files/10x BSC2 P2 Beefy-9 low FGF/XY06/image_XY06_Overlay.tif]

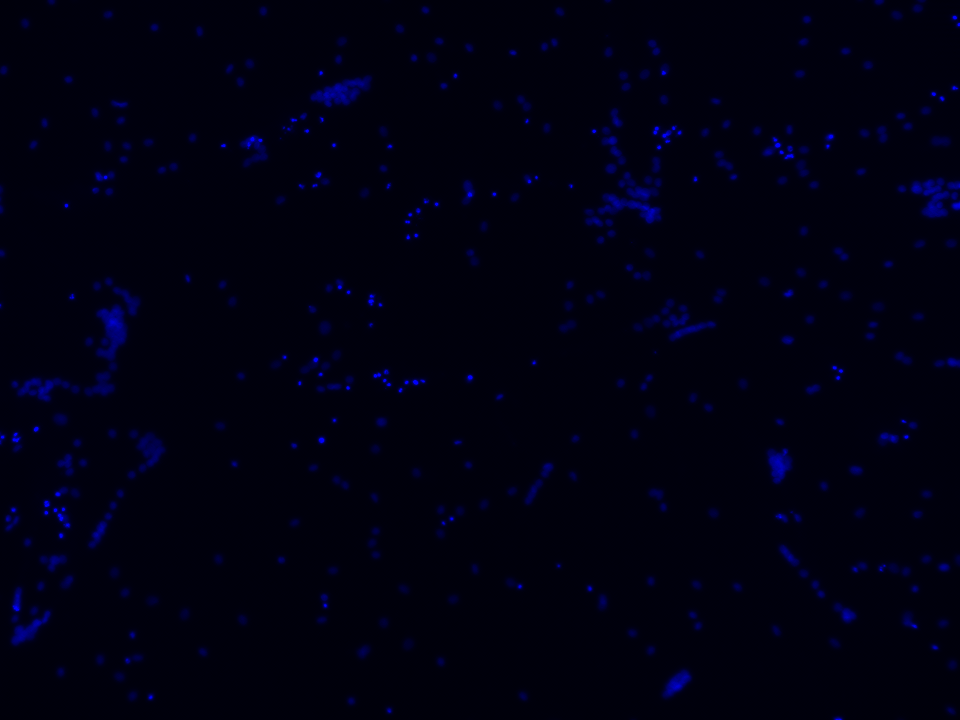

Supplement: Supplementary file 4 — Supplementary Data 1 [file 42003_2022_3423_MOESM4_ESM.zip › Figure S7 (Images)/Raw image files/10x BSC2 P2 Beefy-9 low FGF/XY07/image_XY07_CH1.tif]

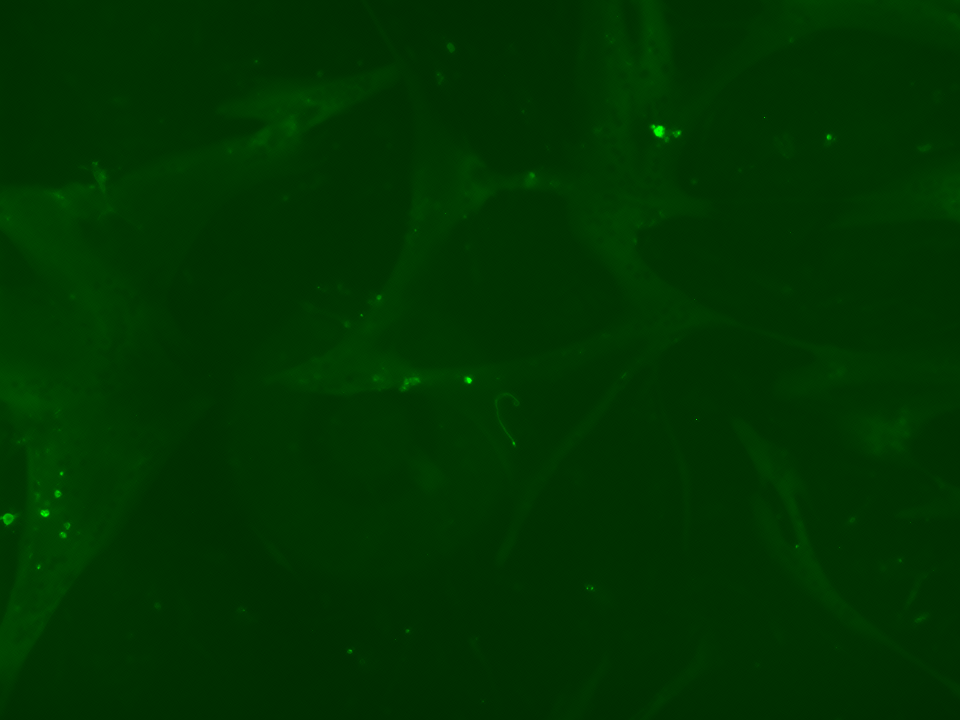

Supplement: Supplementary file 4 — Supplementary Data 1 [file 42003_2022_3423_MOESM4_ESM.zip › Figure S7 (Images)/Raw image files/10x BSC2 P2 Beefy-9 low FGF/XY07/image_XY07_CH2.tif]

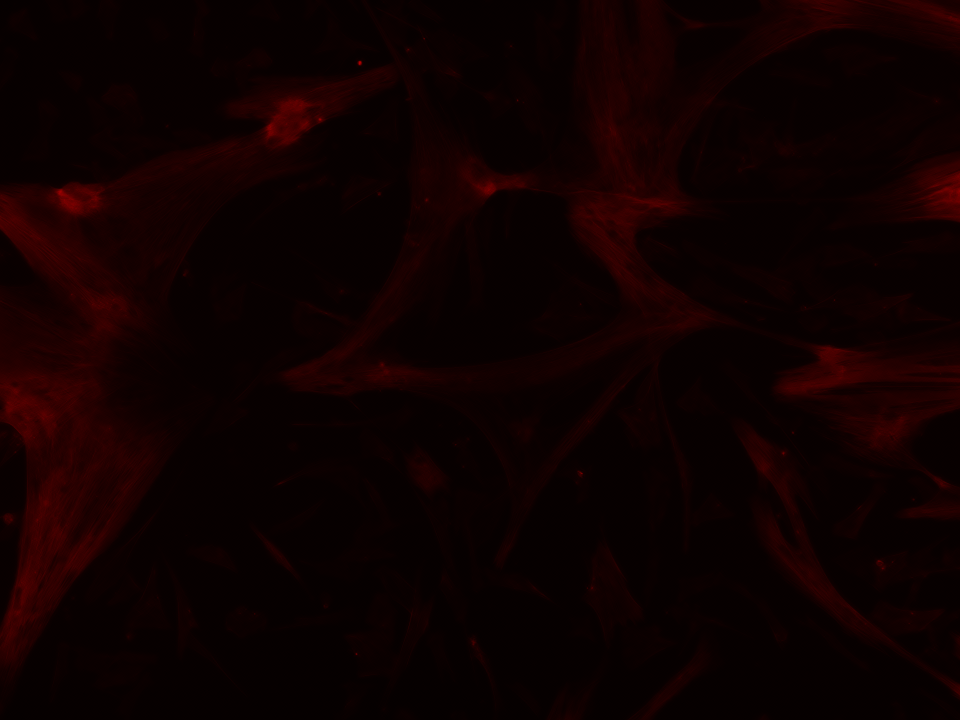

Supplement: Supplementary file 4 — Supplementary Data 1 [file 42003_2022_3423_MOESM4_ESM.zip › Figure S7 (Images)/Raw image files/10x BSC2 P2 Beefy-9 low FGF/XY07/image_XY07_CH3.tif]

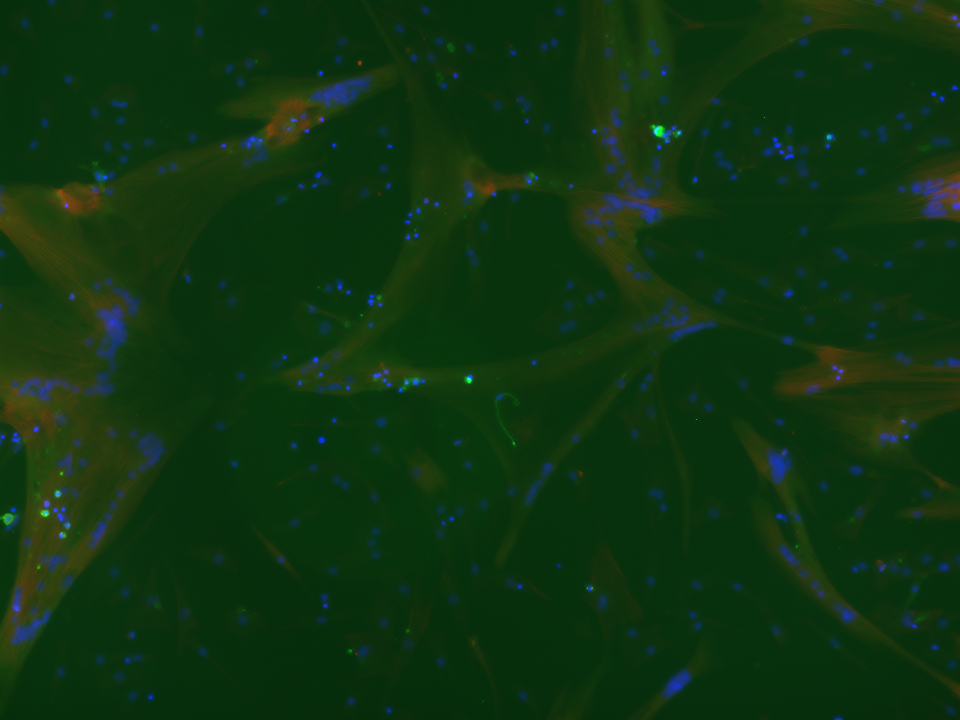

Supplement: Supplementary file 4 — Supplementary Data 1 [file 42003_2022_3423_MOESM4_ESM.zip › Figure S7 (Images)/Raw image files/10x BSC2 P2 Beefy-9 low FGF/XY07/image_XY07_Overlay.tif]

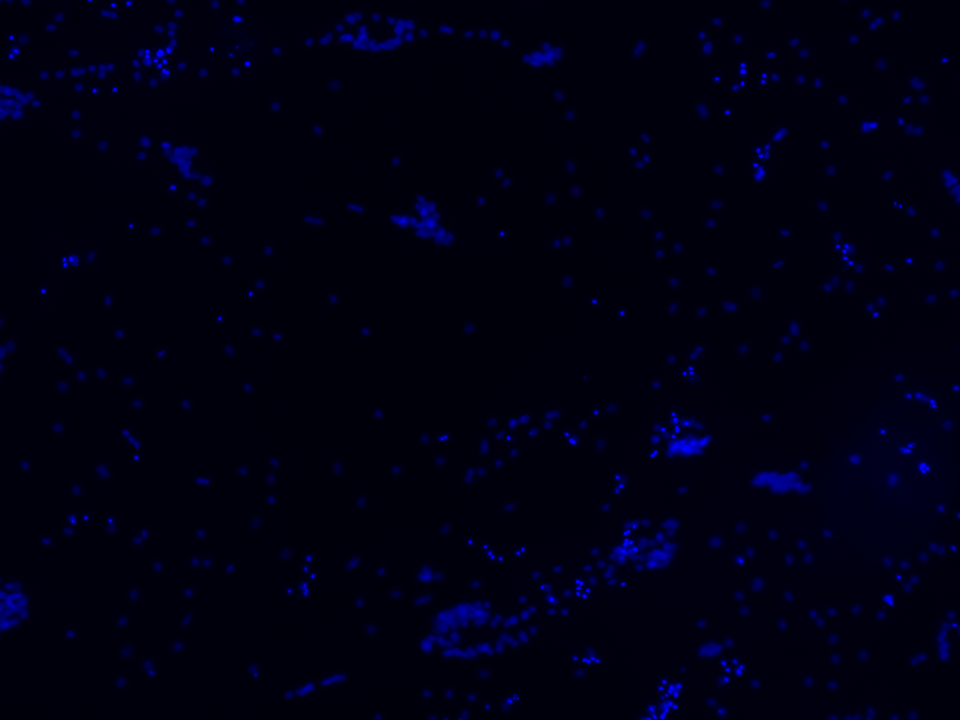

Supplement: Supplementary file 4 — Supplementary Data 1 [file 42003_2022_3423_MOESM4_ESM.zip › Figure S7 (Images)/Raw image files/10x BSC2 P2 Beefy-9 low FGF/XY08/image_XY08_CH1.tif]

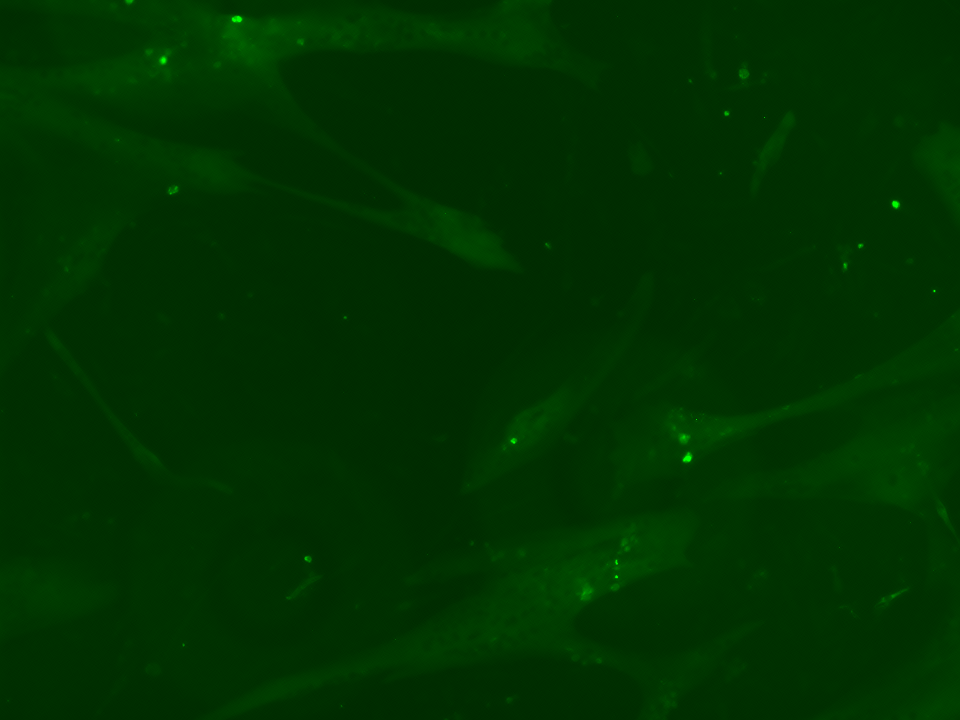

Supplement: Supplementary file 4 — Supplementary Data 1 [file 42003_2022_3423_MOESM4_ESM.zip › Figure S7 (Images)/Raw image files/10x BSC2 P2 Beefy-9 low FGF/XY08/image_XY08_CH2.tif]

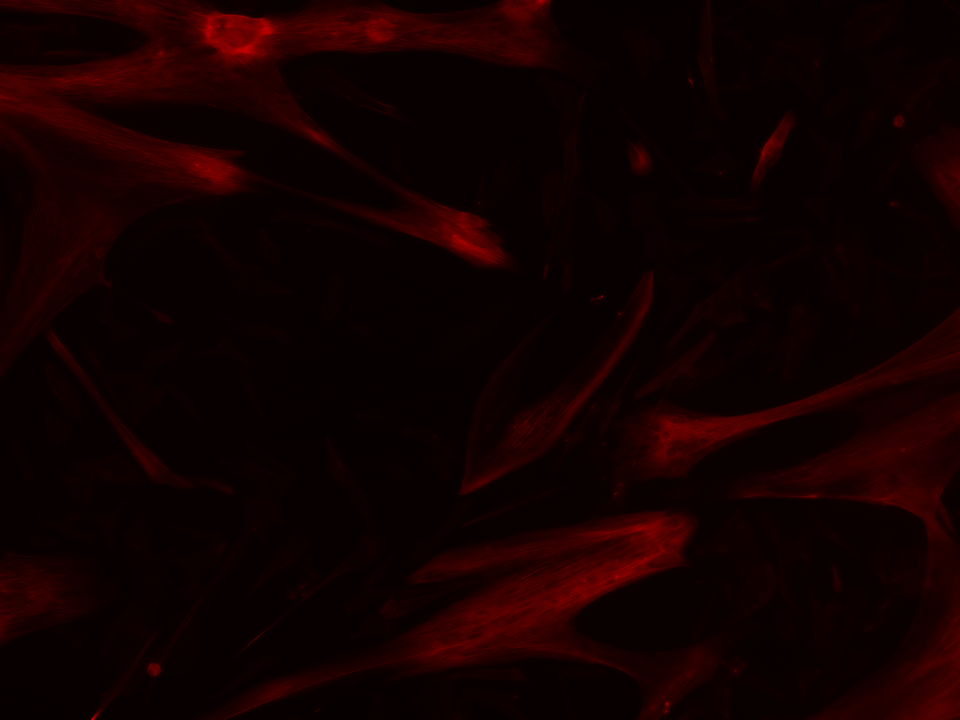

Supplement: Supplementary file 4 — Supplementary Data 1 [file 42003_2022_3423_MOESM4_ESM.zip › Figure S7 (Images)/Raw image files/10x BSC2 P2 Beefy-9 low FGF/XY08/image_XY08_CH3.tif]

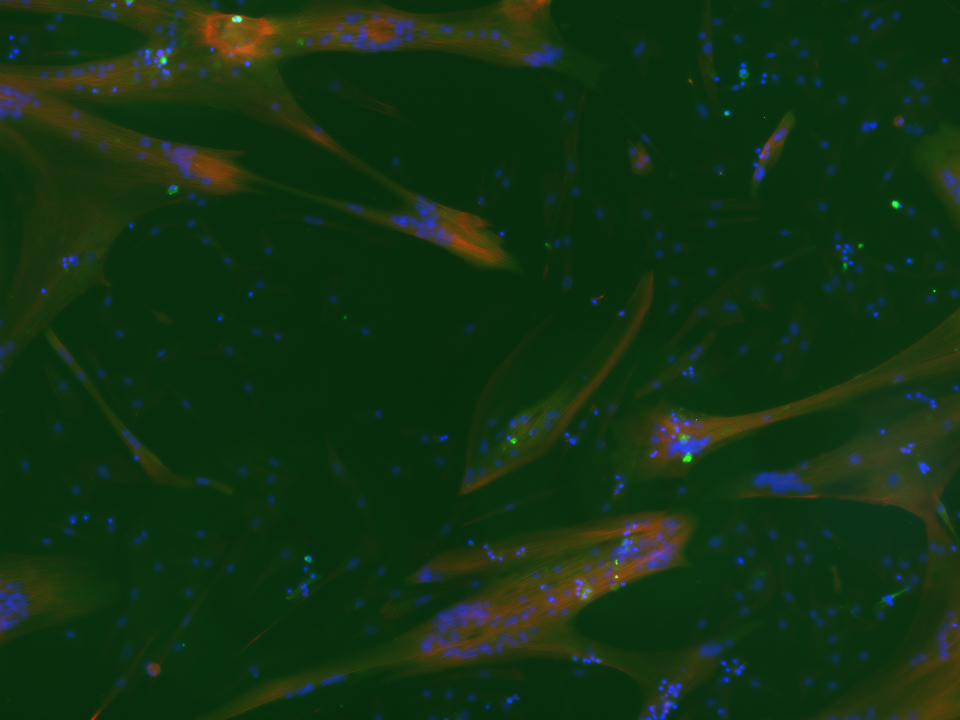

Supplement: Supplementary file 4 — Supplementary Data 1 [file 42003_2022_3423_MOESM4_ESM.zip › Figure S7 (Images)/Raw image files/10x BSC2 P2 Beefy-9 low FGF/XY08/image_XY08_Overlay.tif]

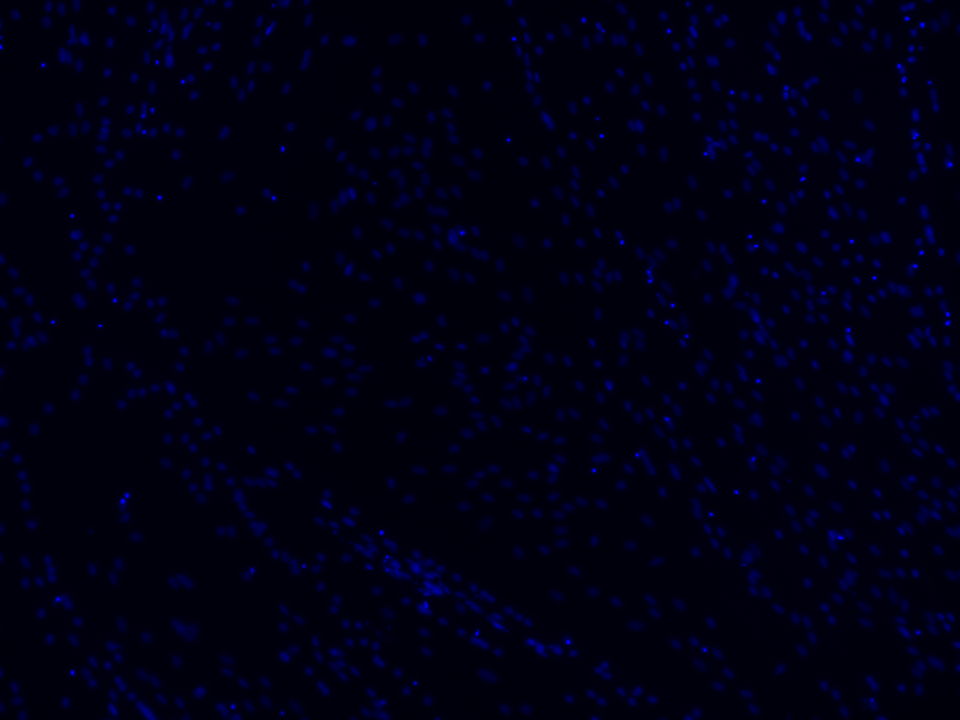

Supplement: Supplementary file 4 — Supplementary Data 1 [file 42003_2022_3423_MOESM4_ESM.zip › Figure S7 (Images)/Raw image files/10x BSC2 P2 BSC-GM/XY01/image_XY01_CH1.tif]

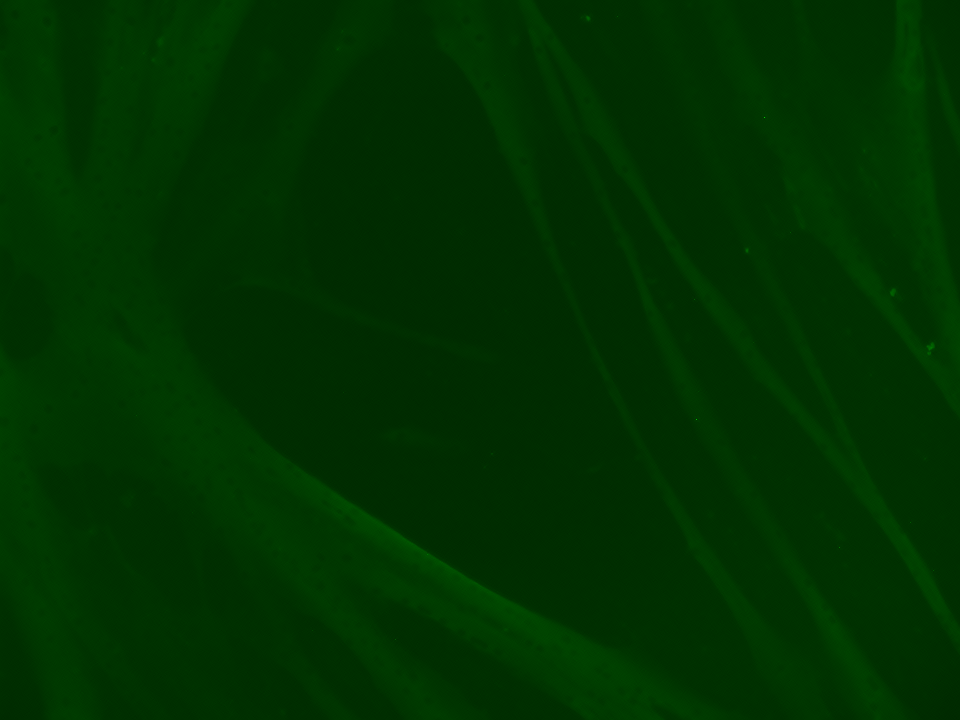

Supplement: Supplementary file 4 — Supplementary Data 1 [file 42003_2022_3423_MOESM4_ESM.zip › Figure S7 (Images)/Raw image files/10x BSC2 P2 BSC-GM/XY01/image_XY01_CH2.tif]

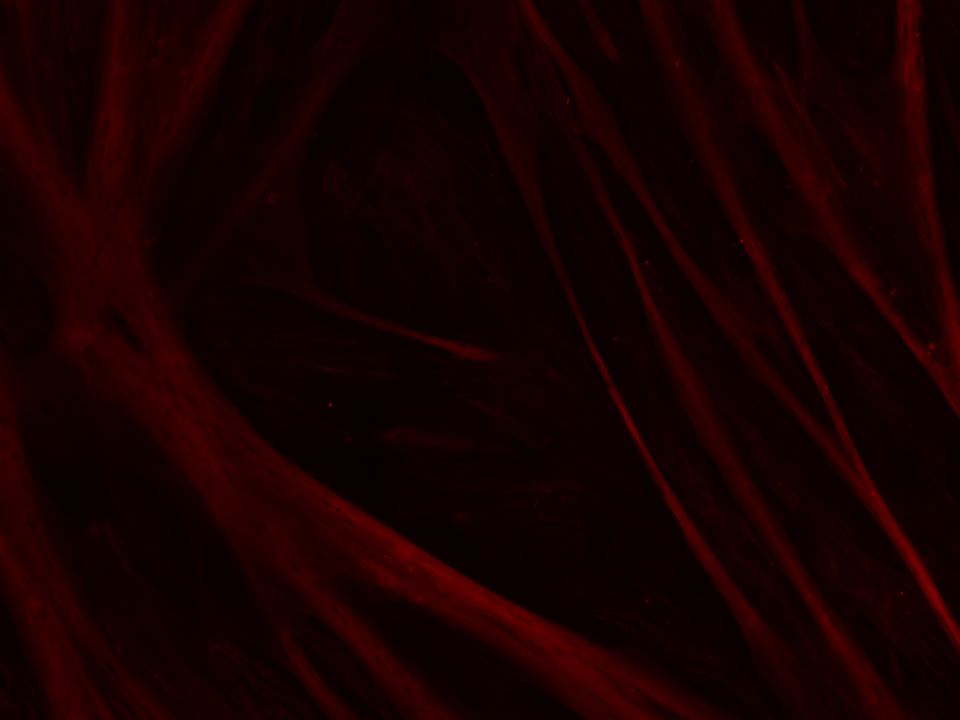

Supplement: Supplementary file 4 — Supplementary Data 1 [file 42003_2022_3423_MOESM4_ESM.zip › Figure S7 (Images)/Raw image files/10x BSC2 P2 BSC-GM/XY01/image_XY01_CH3.tif]

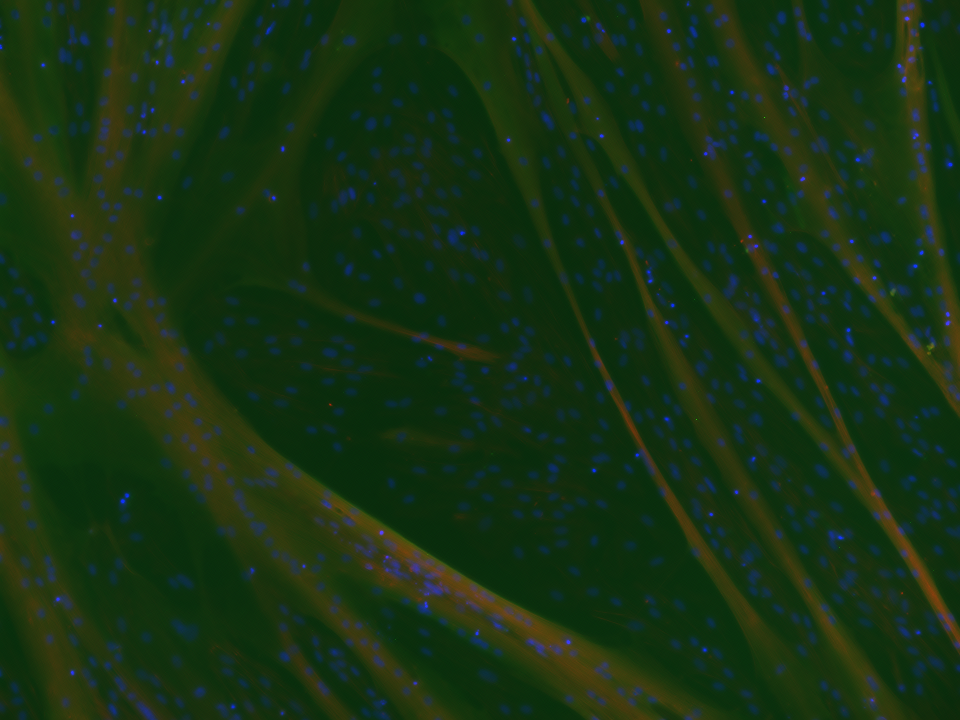

Supplement: Supplementary file 4 — Supplementary Data 1 [file 42003_2022_3423_MOESM4_ESM.zip › Figure S7 (Images)/Raw image files/10x BSC2 P2 BSC-GM/XY01/image_XY01_Overlay.tif]

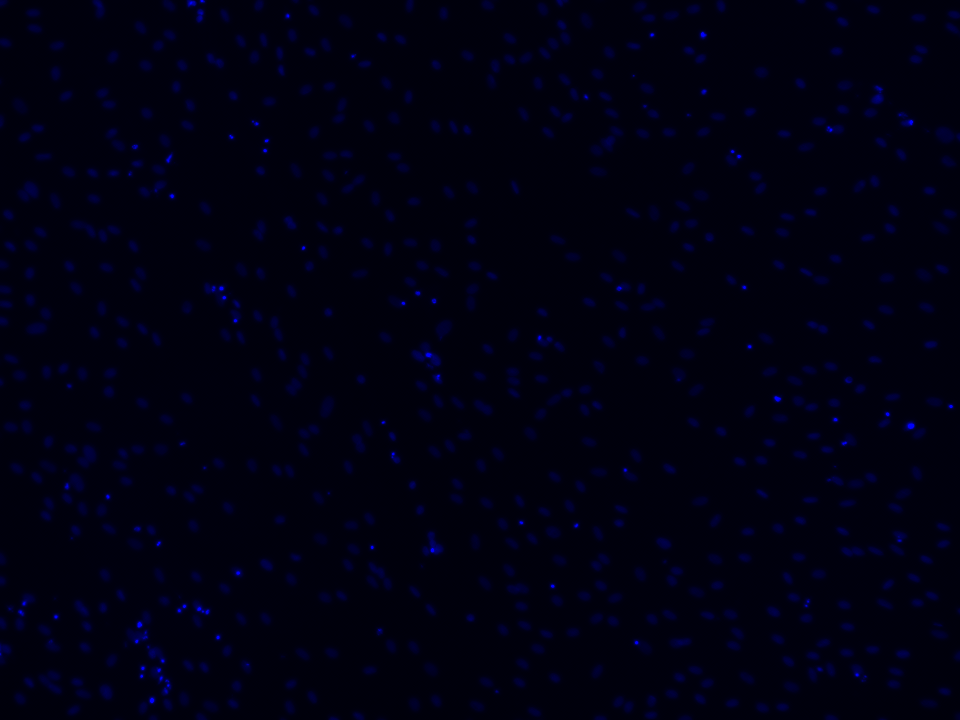

Supplement: Supplementary file 4 — Supplementary Data 1 [file 42003_2022_3423_MOESM4_ESM.zip › Figure S7 (Images)/Raw image files/10x BSC2 P2 BSC-GM/XY02/image_XY02_CH1.tif]

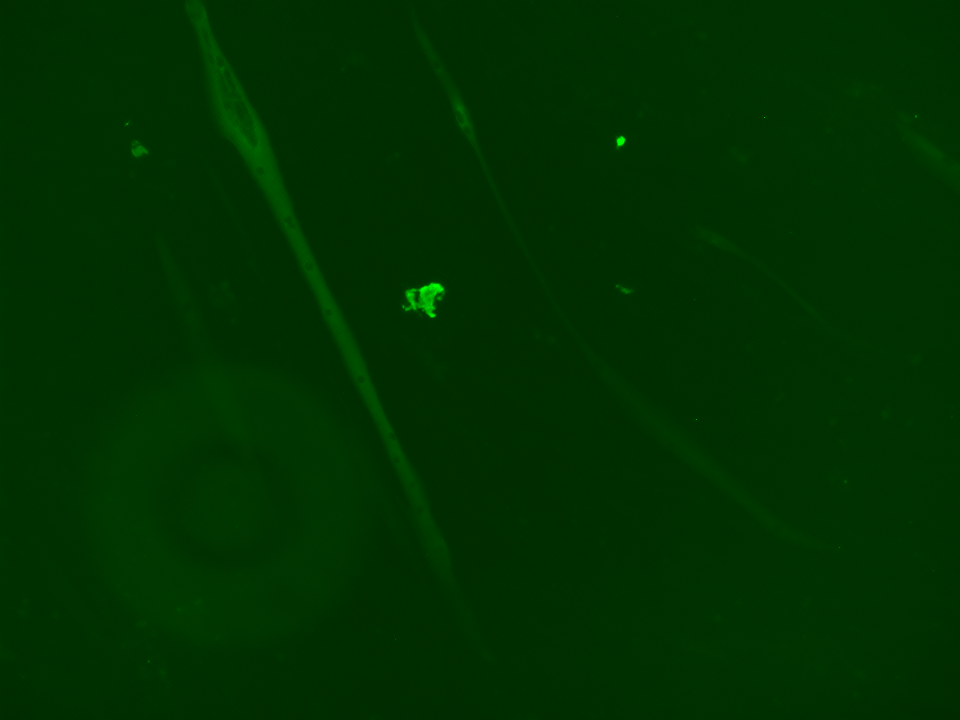

Supplement: Supplementary file 4 — Supplementary Data 1 [file 42003_2022_3423_MOESM4_ESM.zip › Figure S7 (Images)/Raw image files/10x BSC2 P2 BSC-GM/XY02/image_XY02_CH2.tif]

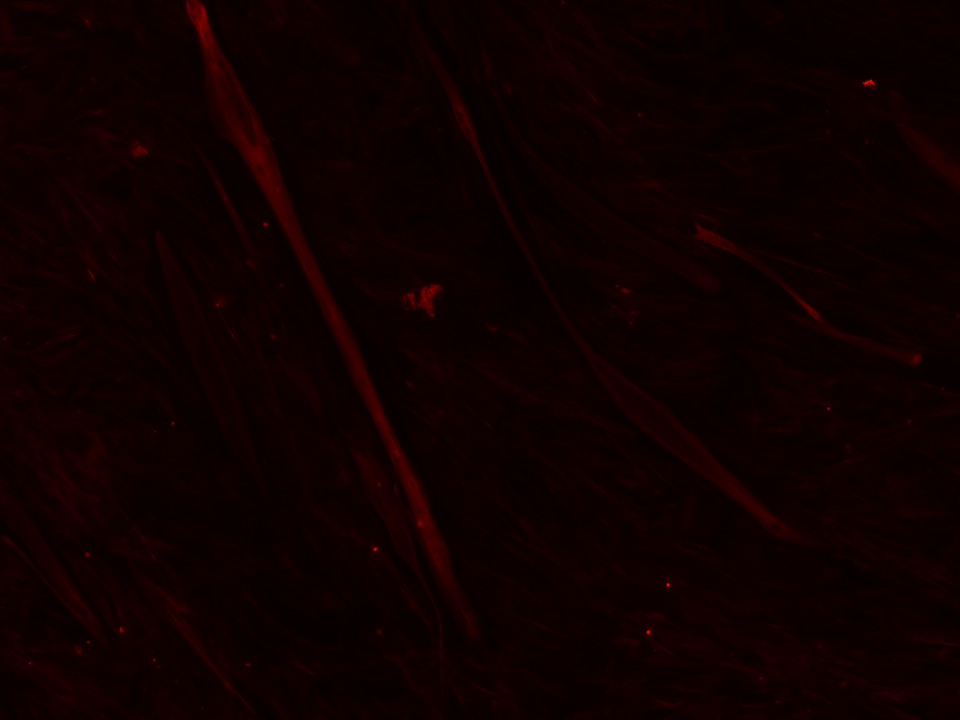

Supplement: Supplementary file 4 — Supplementary Data 1 [file 42003_2022_3423_MOESM4_ESM.zip › Figure S7 (Images)/Raw image files/10x BSC2 P2 BSC-GM/XY02/image_XY02_CH3.tif]

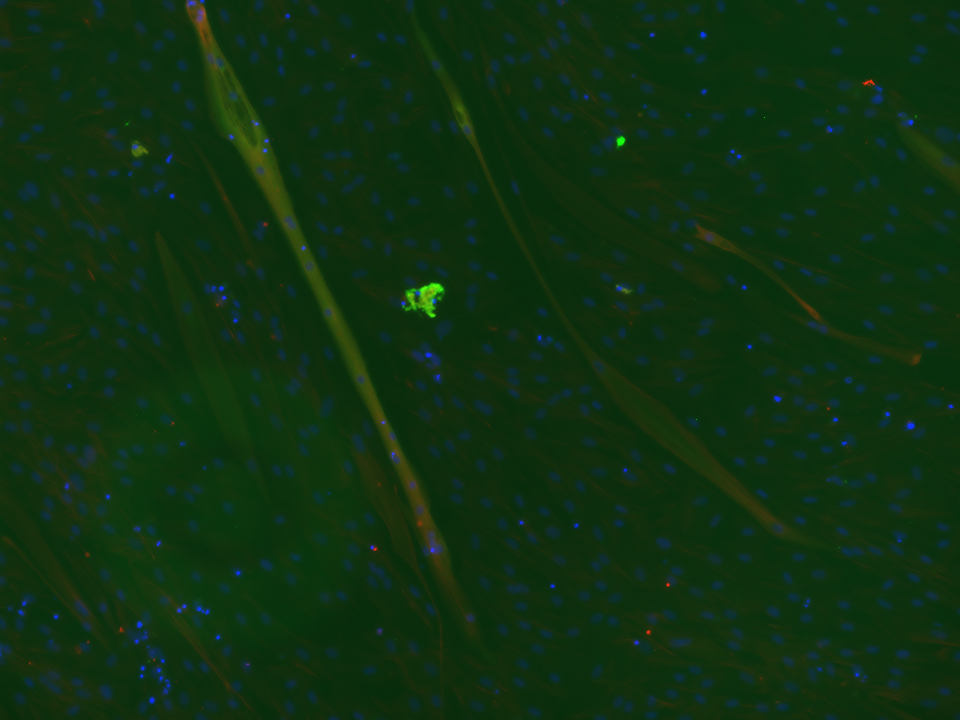

Supplement: Supplementary file 4 — Supplementary Data 1 [file 42003_2022_3423_MOESM4_ESM.zip › Figure S7 (Images)/Raw image files/10x BSC2 P2 BSC-GM/XY02/image_XY02_Overlay.tif]

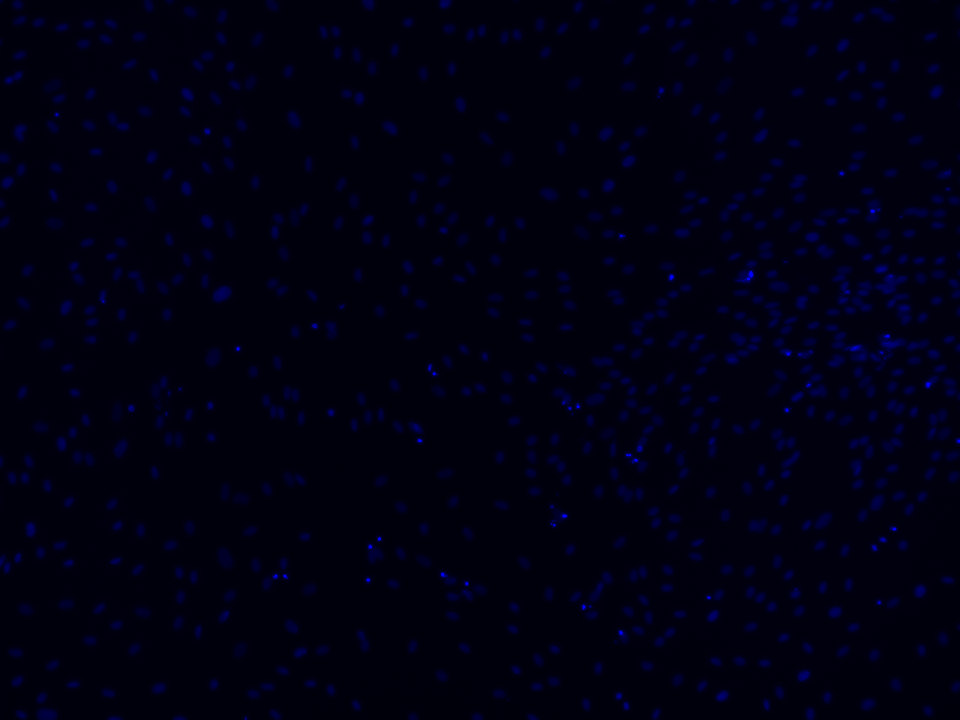

Supplement: Supplementary file 4 — Supplementary Data 1 [file 42003_2022_3423_MOESM4_ESM.zip › Figure S7 (Images)/Raw image files/10x BSC2 P2 BSC-GM/XY03/image_XY03_CH1.tif]

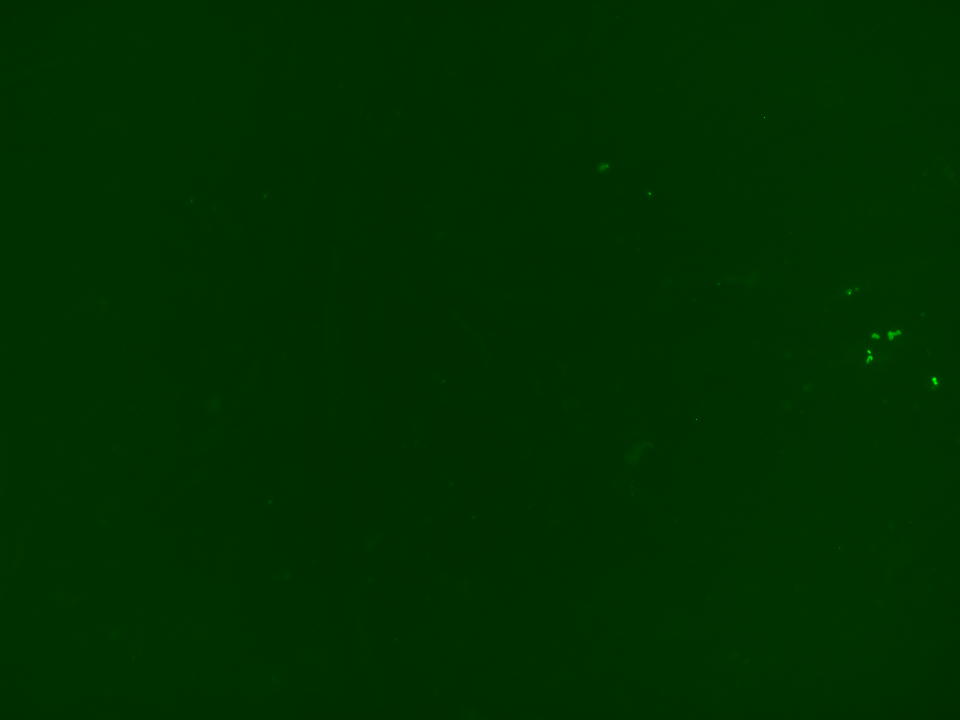

Supplement: Supplementary file 4 — Supplementary Data 1 [file 42003_2022_3423_MOESM4_ESM.zip › Figure S7 (Images)/Raw image files/10x BSC2 P2 BSC-GM/XY03/image_XY03_CH2.tif]

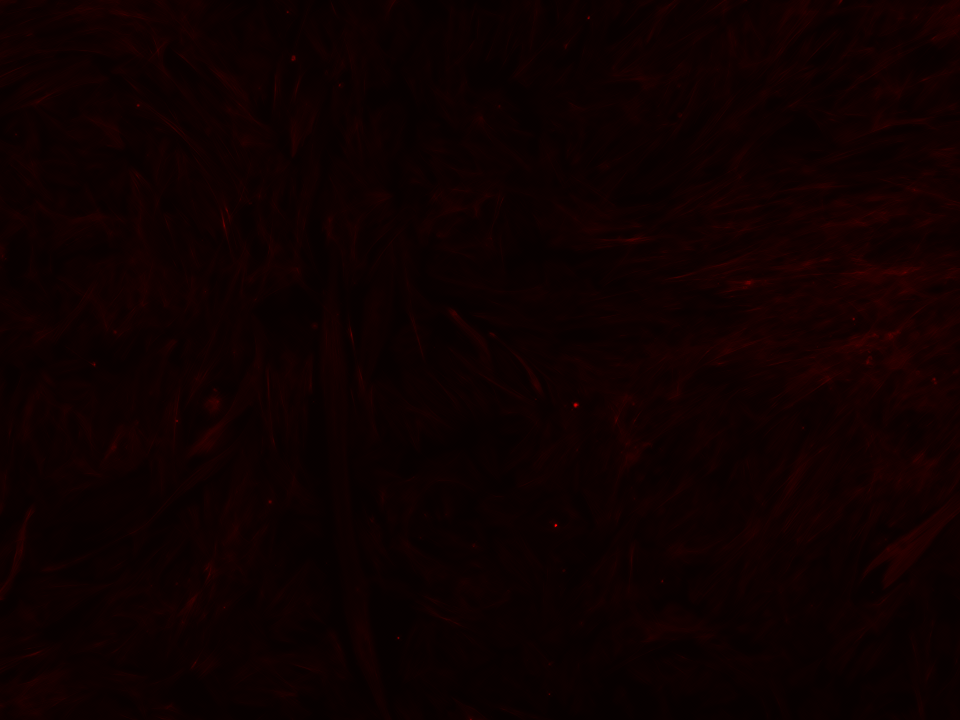

Supplement: Supplementary file 4 — Supplementary Data 1 [file 42003_2022_3423_MOESM4_ESM.zip › Figure S7 (Images)/Raw image files/10x BSC2 P2 BSC-GM/XY03/image_XY03_CH3.tif]

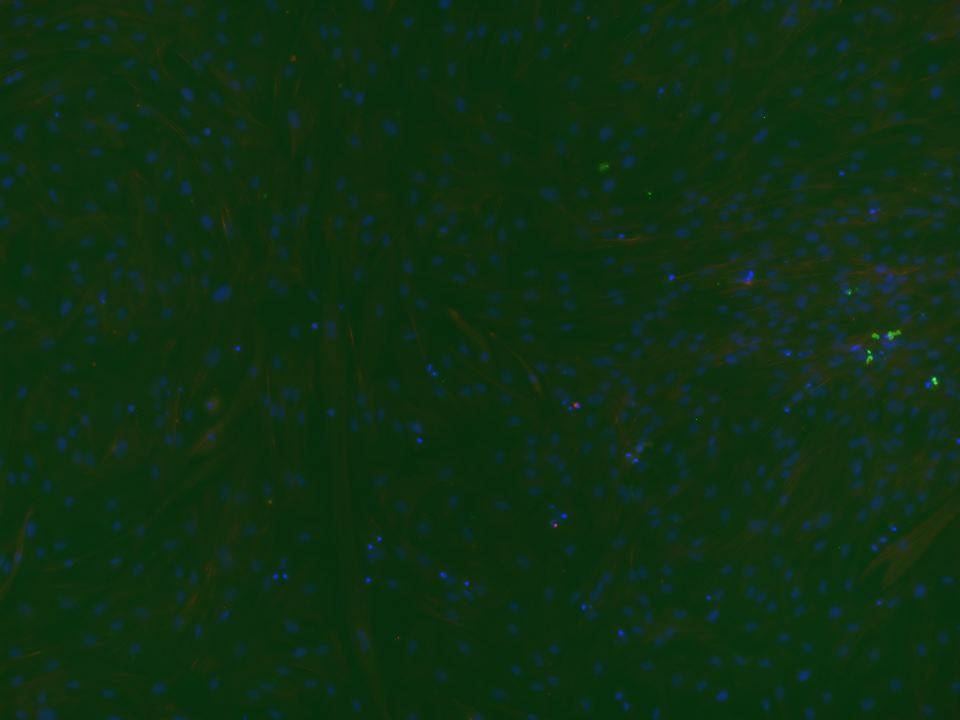

Supplement: Supplementary file 4 — Supplementary Data 1 [file 42003_2022_3423_MOESM4_ESM.zip › Figure S7 (Images)/Raw image files/10x BSC2 P2 BSC-GM/XY03/image_XY03_Overlay.tif]

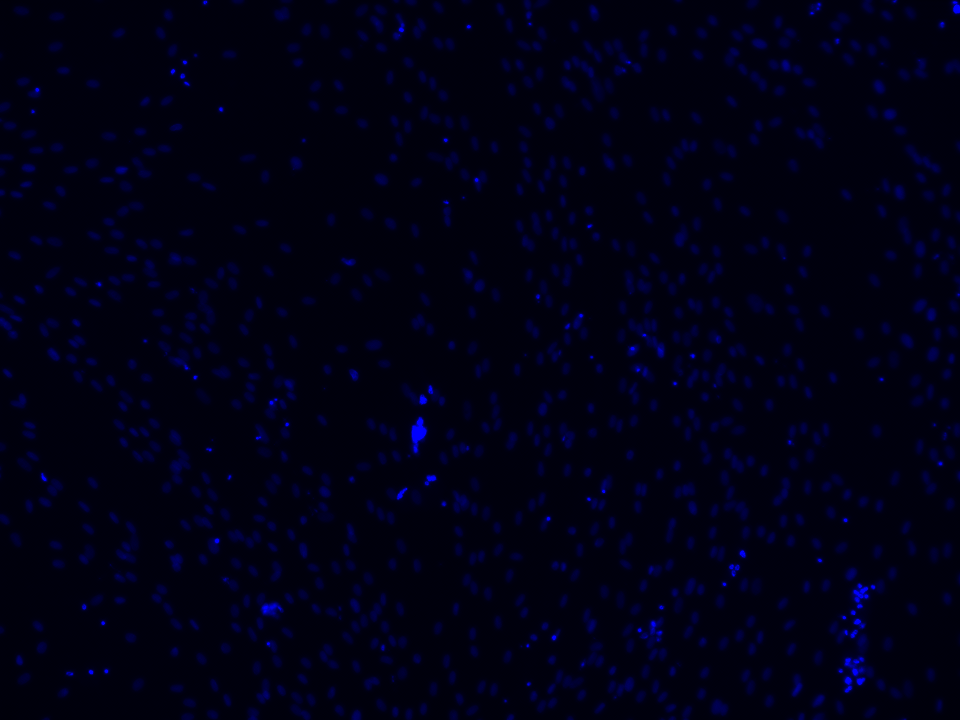

Supplement: Supplementary file 4 — Supplementary Data 1 [file 42003_2022_3423_MOESM4_ESM.zip › Figure S7 (Images)/Raw image files/10x BSC2 P2 BSC-GM/XY04/image_XY04_CH1.tif]

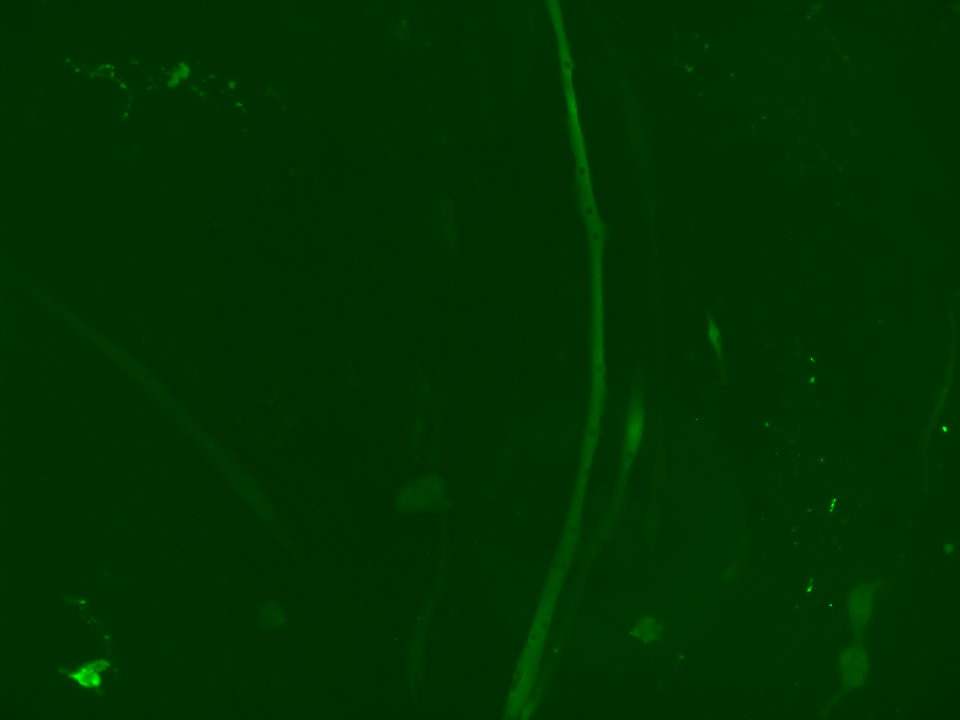

Supplement: Supplementary file 4 — Supplementary Data 1 [file 42003_2022_3423_MOESM4_ESM.zip › Figure S7 (Images)/Raw image files/10x BSC2 P2 BSC-GM/XY04/image_XY04_CH2.tif]

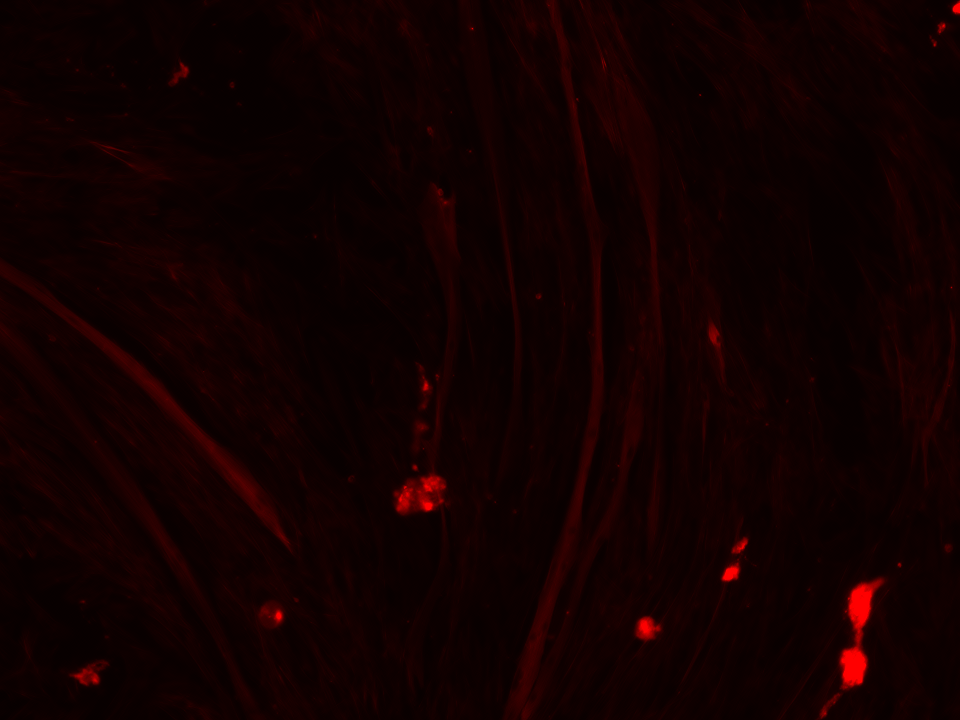

Supplement: Supplementary file 4 — Supplementary Data 1 [file 42003_2022_3423_MOESM4_ESM.zip › Figure S7 (Images)/Raw image files/10x BSC2 P2 BSC-GM/XY04/image_XY04_CH3.tif]

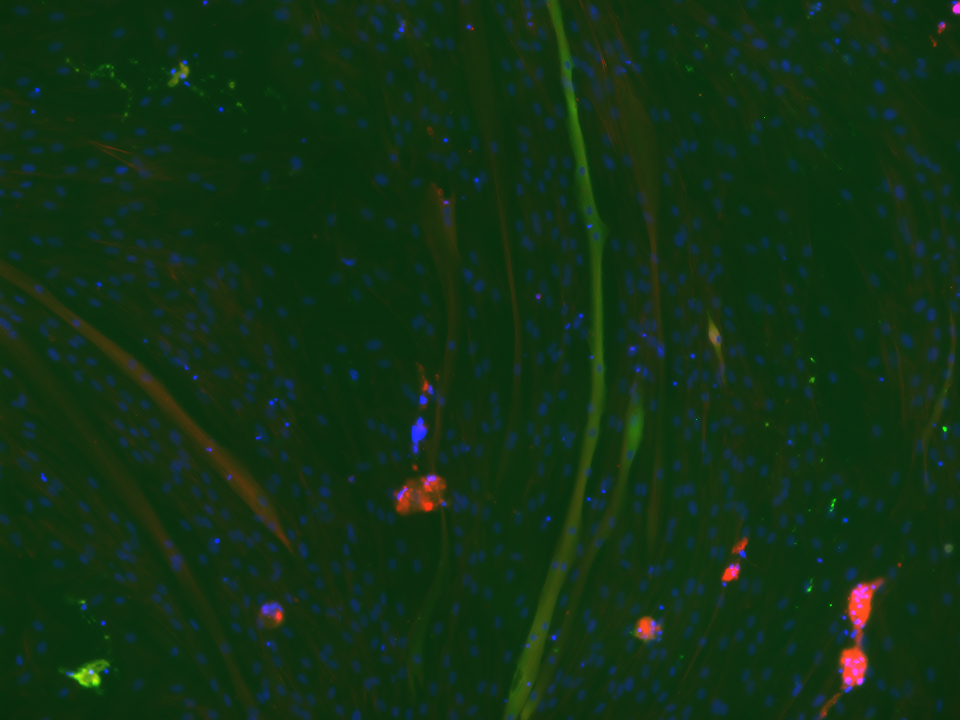

Supplement: Supplementary file 4 — Supplementary Data 1 [file 42003_2022_3423_MOESM4_ESM.zip › Figure S7 (Images)/Raw image files/10x BSC2 P2 BSC-GM/XY04/image_XY04_Overlay.tif]

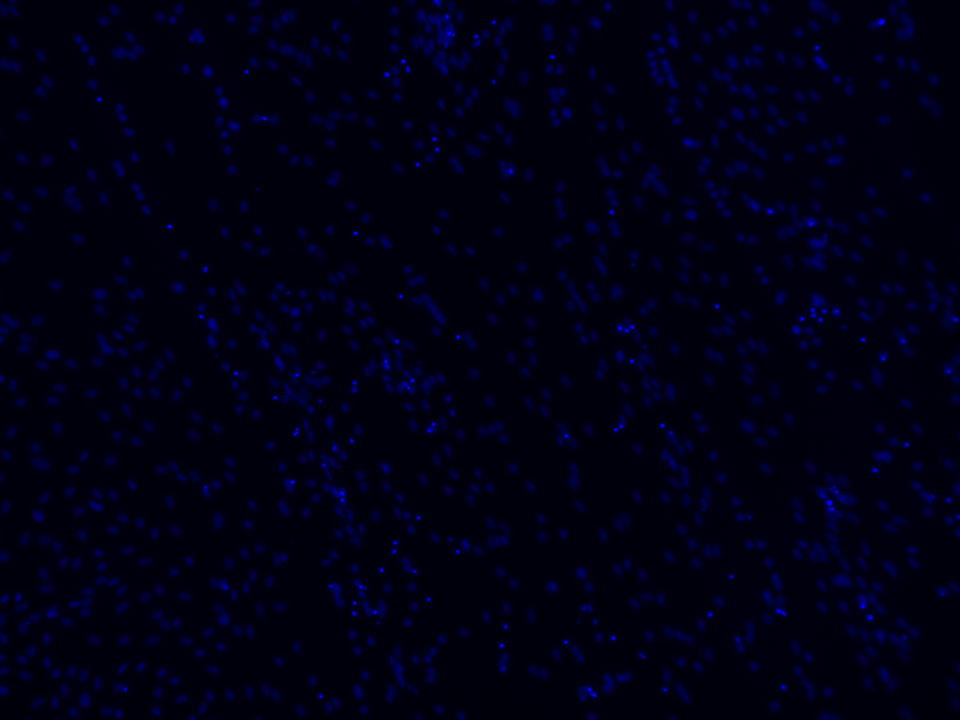

Supplement: Supplementary file 4 — Supplementary Data 1 [file 42003_2022_3423_MOESM4_ESM.zip › Figure S7 (Images)/Raw image files/10x BSC2 P2 BSC-GM/XY05/image_XY05_CH1.tif]

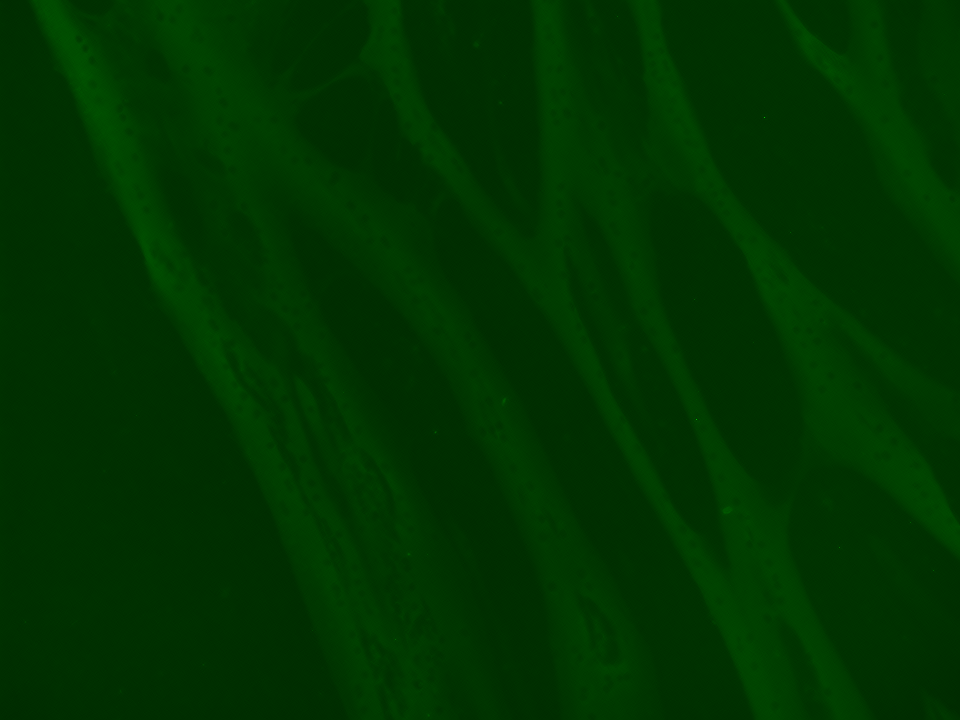

Supplement: Supplementary file 4 — Supplementary Data 1 [file 42003_2022_3423_MOESM4_ESM.zip › Figure S7 (Images)/Raw image files/10x BSC2 P2 BSC-GM/XY05/image_XY05_CH2.tif]

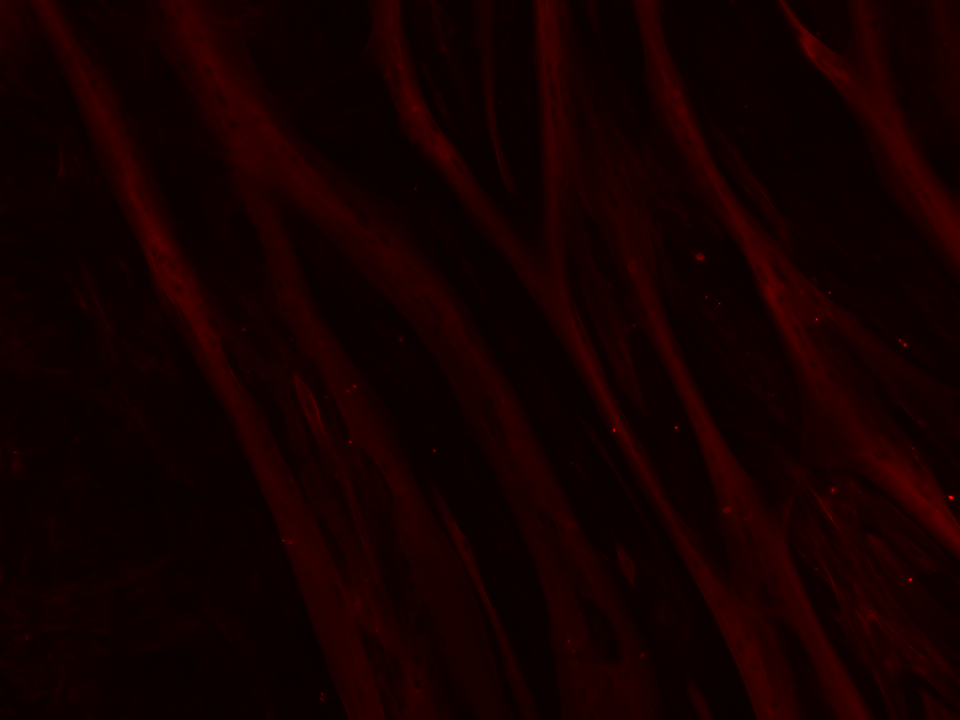

Supplement: Supplementary file 4 — Supplementary Data 1 [file 42003_2022_3423_MOESM4_ESM.zip › Figure S7 (Images)/Raw image files/10x BSC2 P2 BSC-GM/XY05/image_XY05_CH3.tif]

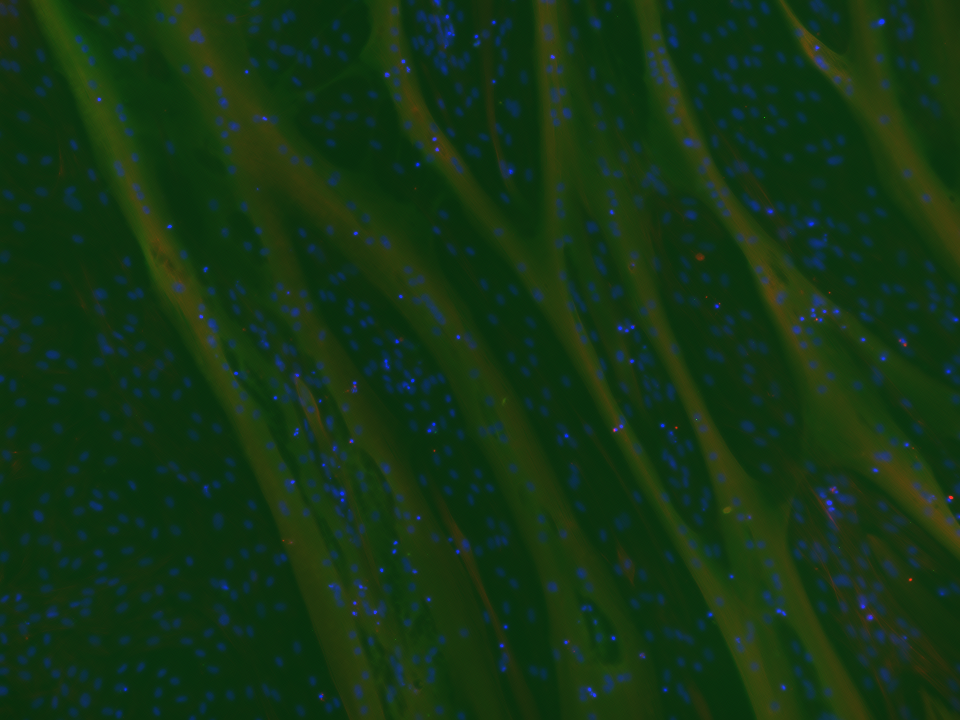

Supplement: Supplementary file 4 — Supplementary Data 1 [file 42003_2022_3423_MOESM4_ESM.zip › Figure S7 (Images)/Raw image files/10x BSC2 P2 BSC-GM/XY05/image_XY05_Overlay.tif]

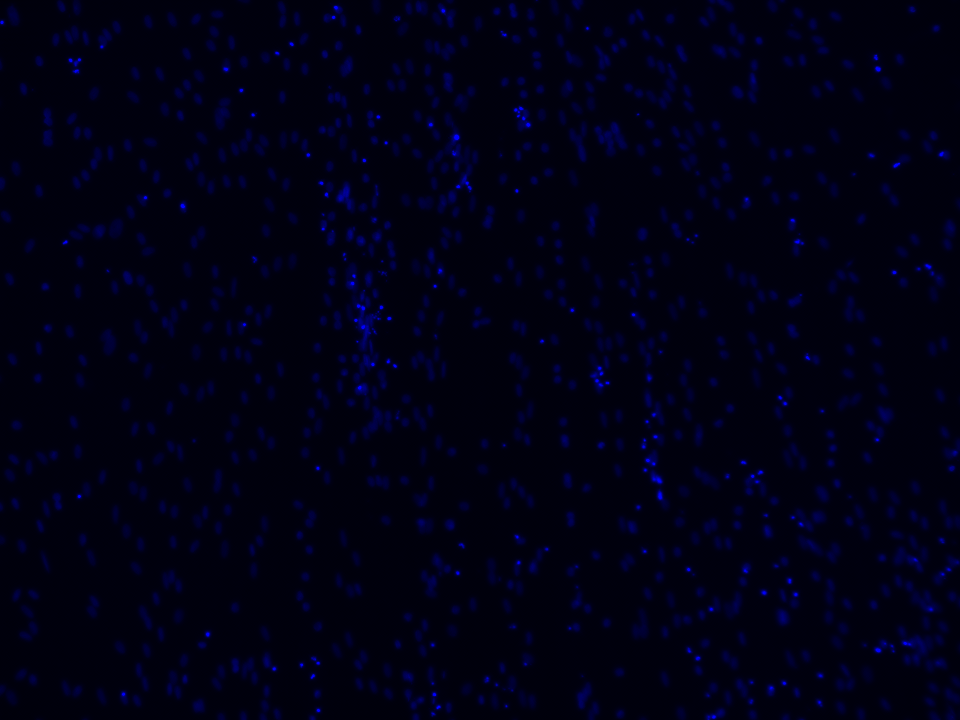

Supplement: Supplementary file 4 — Supplementary Data 1 [file 42003_2022_3423_MOESM4_ESM.zip › Figure S7 (Images)/Raw image files/10x BSC2 P2 BSC-GM/XY06/image_XY06_CH1.tif]

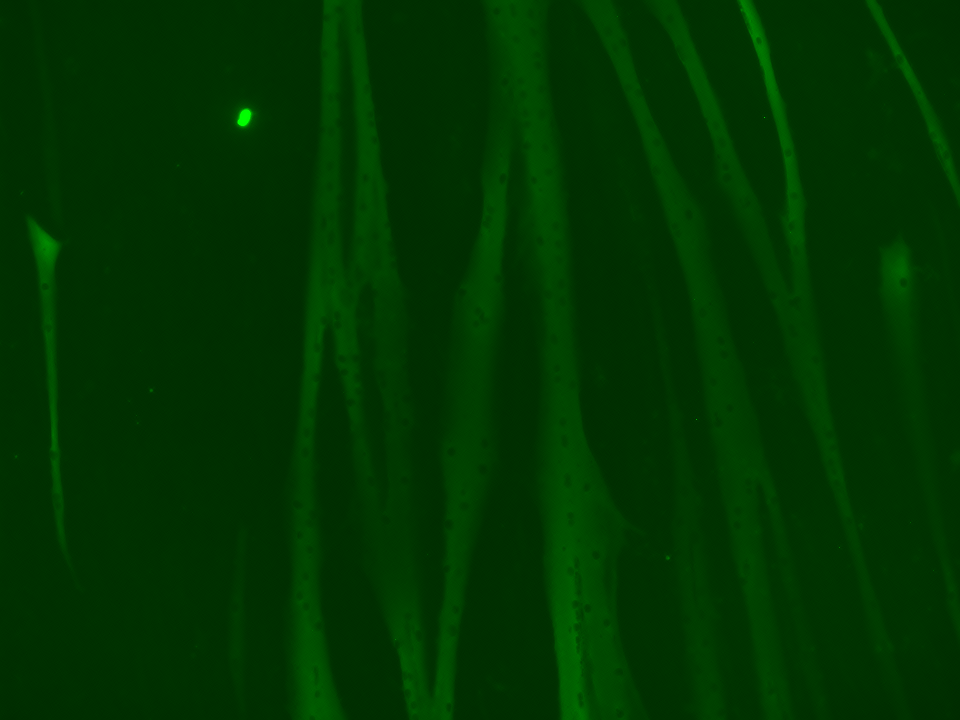

Supplement: Supplementary file 4 — Supplementary Data 1 [file 42003_2022_3423_MOESM4_ESM.zip › Figure S7 (Images)/Raw image files/10x BSC2 P2 BSC-GM/XY06/image_XY06_CH2.tif]

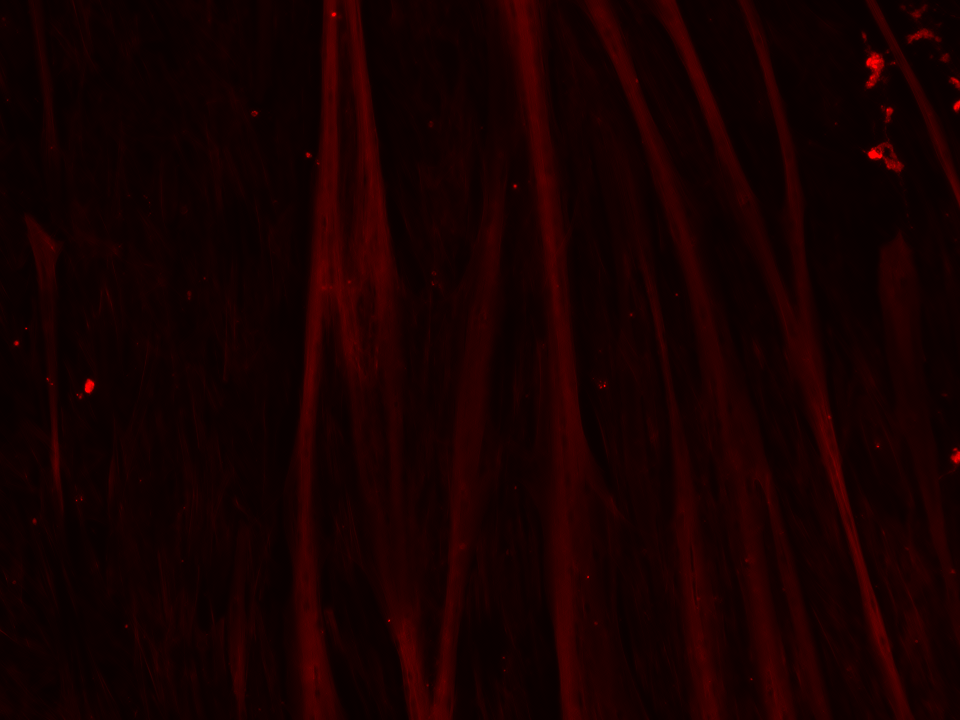

Supplement: Supplementary file 4 — Supplementary Data 1 [file 42003_2022_3423_MOESM4_ESM.zip › Figure S7 (Images)/Raw image files/10x BSC2 P2 BSC-GM/XY06/image_XY06_CH3.tif]

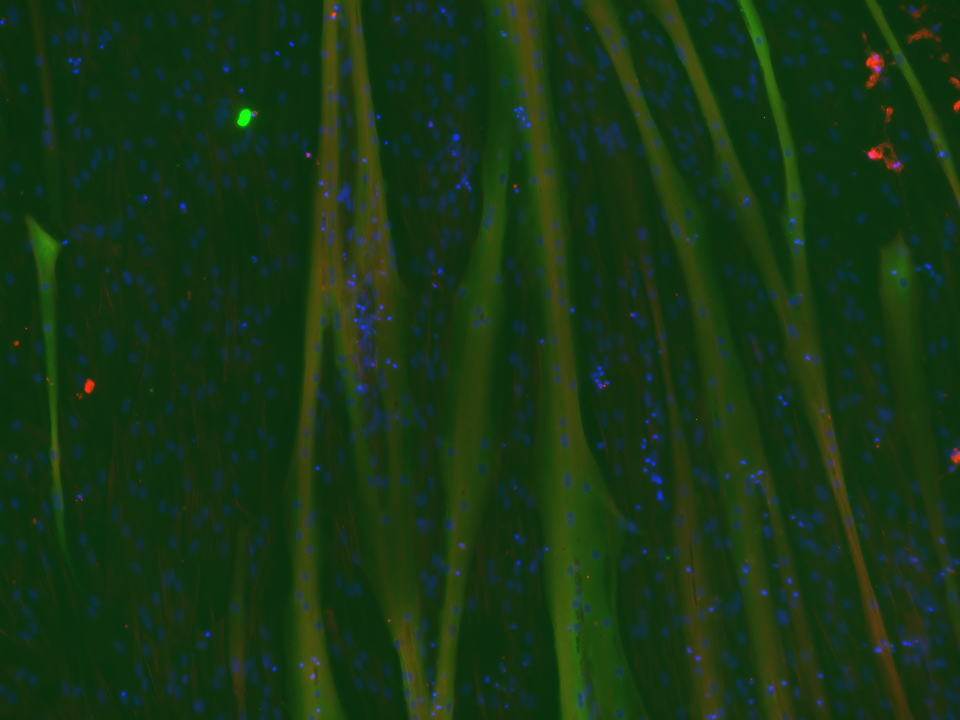

Supplement: Supplementary file 4 — Supplementary Data 1 [file 42003_2022_3423_MOESM4_ESM.zip › Figure S7 (Images)/Raw image files/10x BSC2 P2 BSC-GM/XY06/image_XY06_Overlay.tif]

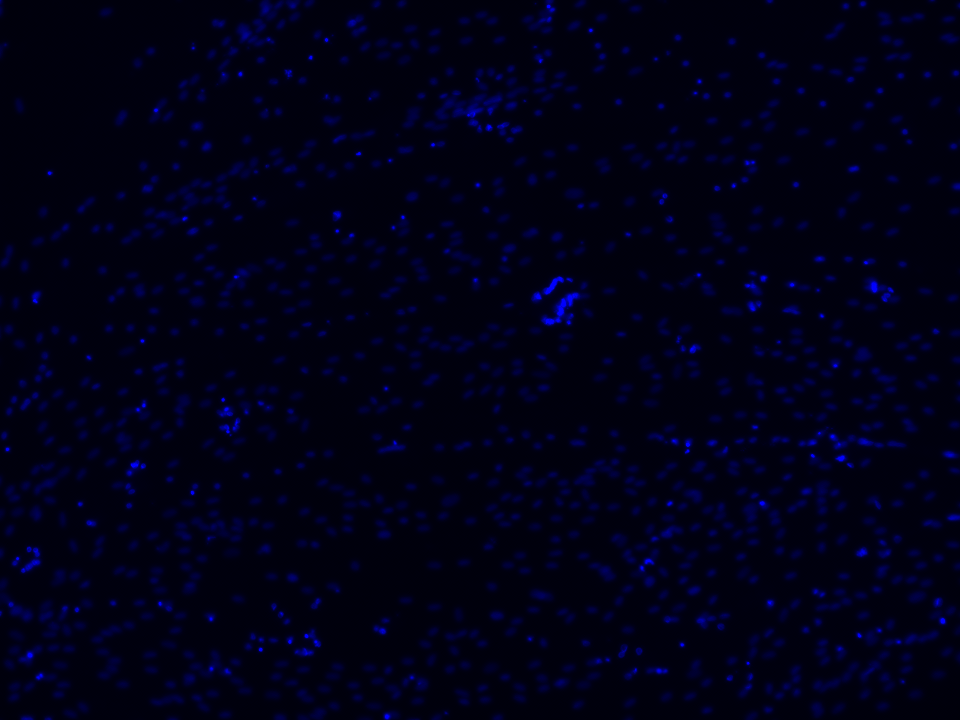

Supplement: Supplementary file 4 — Supplementary Data 1 [file 42003_2022_3423_MOESM4_ESM.zip › Figure S7 (Images)/Raw image files/10x BSC2 P2 BSC-GM/XY07/image_XY07_CH1.tif]

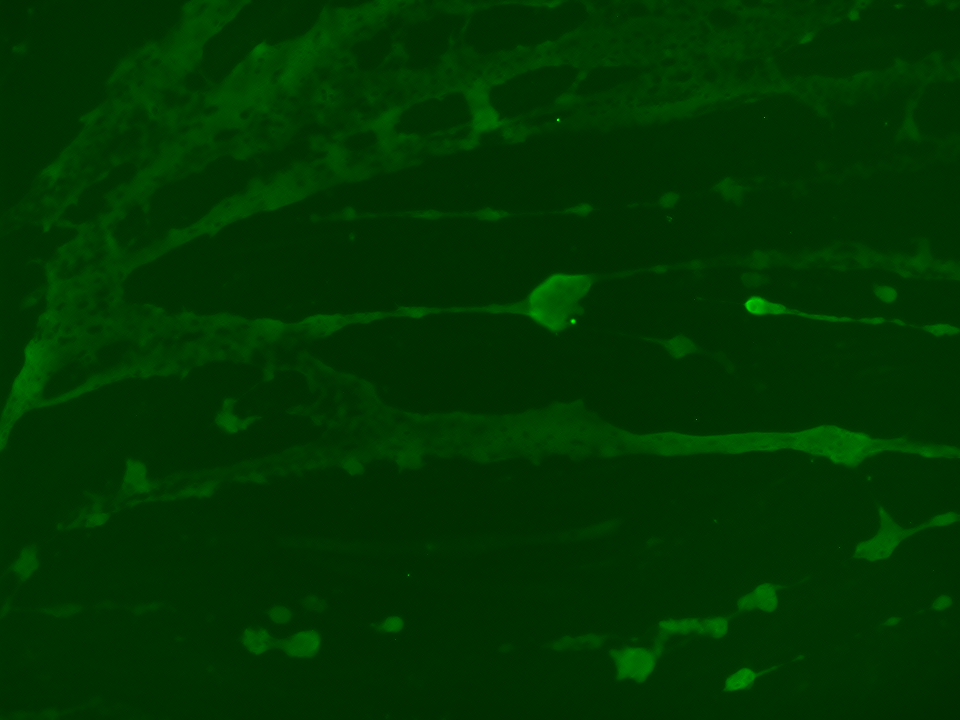

Supplement: Supplementary file 4 — Supplementary Data 1 [file 42003_2022_3423_MOESM4_ESM.zip › Figure S7 (Images)/Raw image files/10x BSC2 P2 BSC-GM/XY07/image_XY07_CH2.tif]

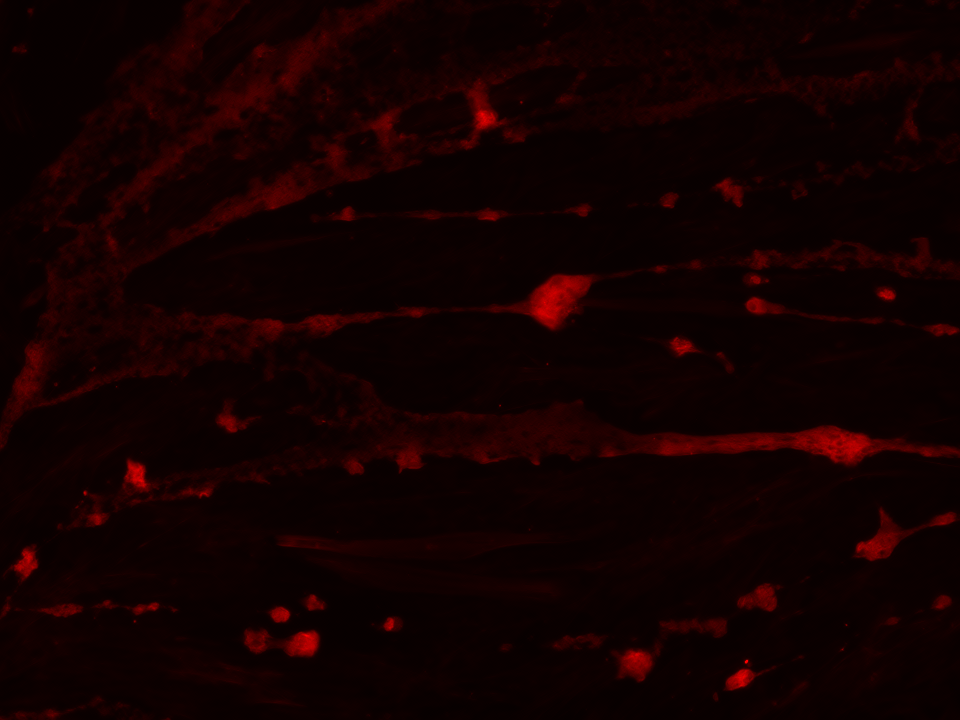

Supplement: Supplementary file 4 — Supplementary Data 1 [file 42003_2022_3423_MOESM4_ESM.zip › Figure S7 (Images)/Raw image files/10x BSC2 P2 BSC-GM/XY07/image_XY07_CH3.tif]

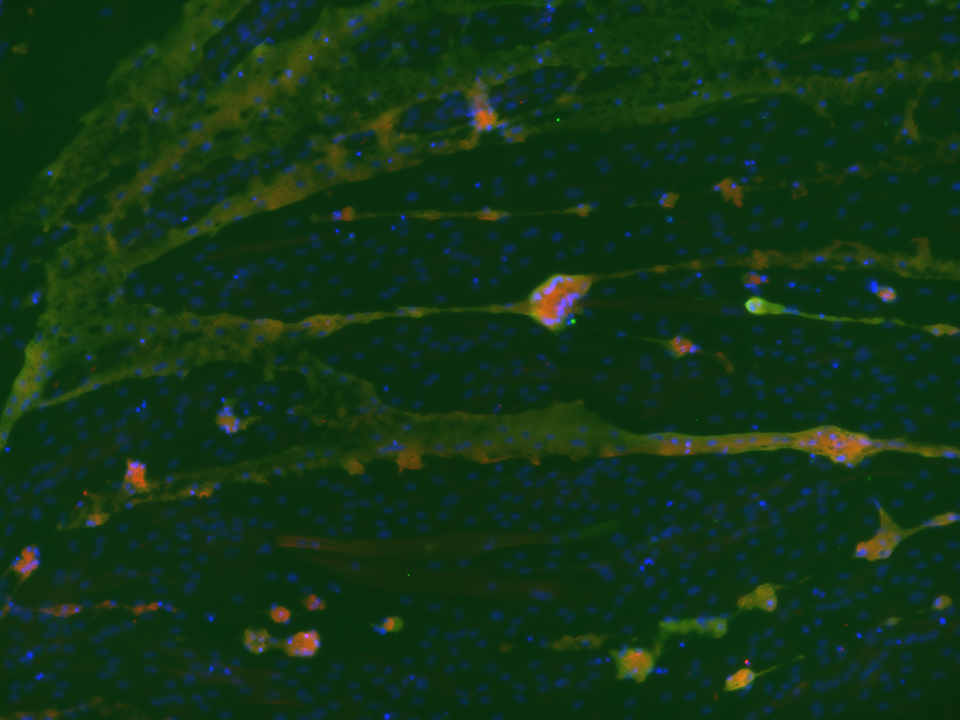

Supplement: Supplementary file 4 — Supplementary Data 1 [file 42003_2022_3423_MOESM4_ESM.zip › Figure S7 (Images)/Raw image files/10x BSC2 P2 BSC-GM/XY07/image_XY07_Overlay.tif]

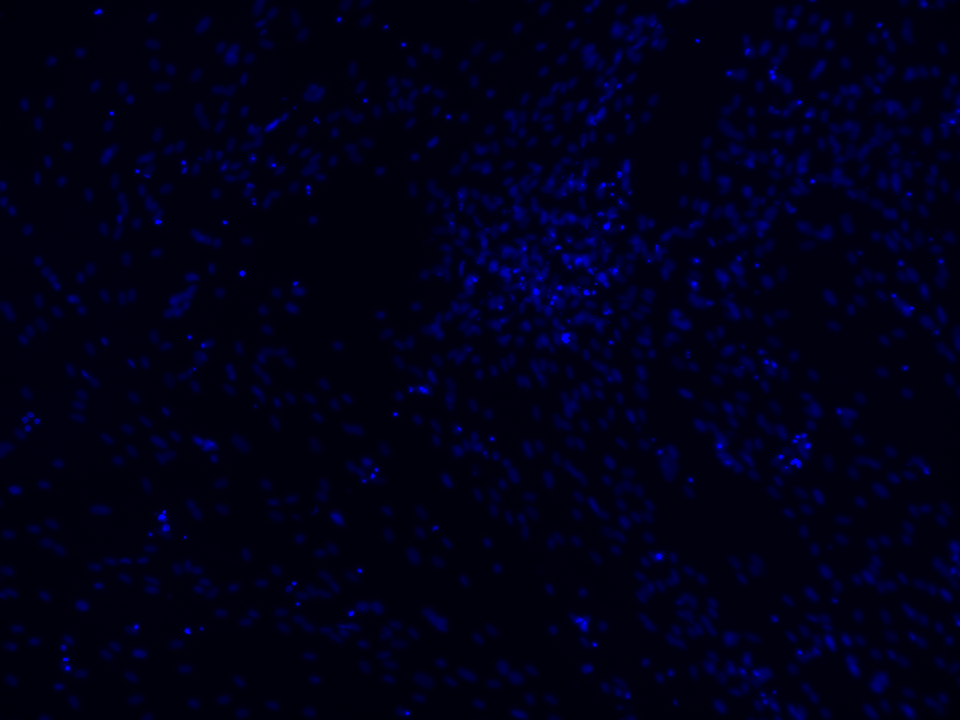

Supplement: Supplementary file 4 — Supplementary Data 1 [file 42003_2022_3423_MOESM4_ESM.zip › Figure S7 (Images)/Raw image files/10x BSC2 P2 BSC-GM/XY08/image_XY08_CH1.tif]

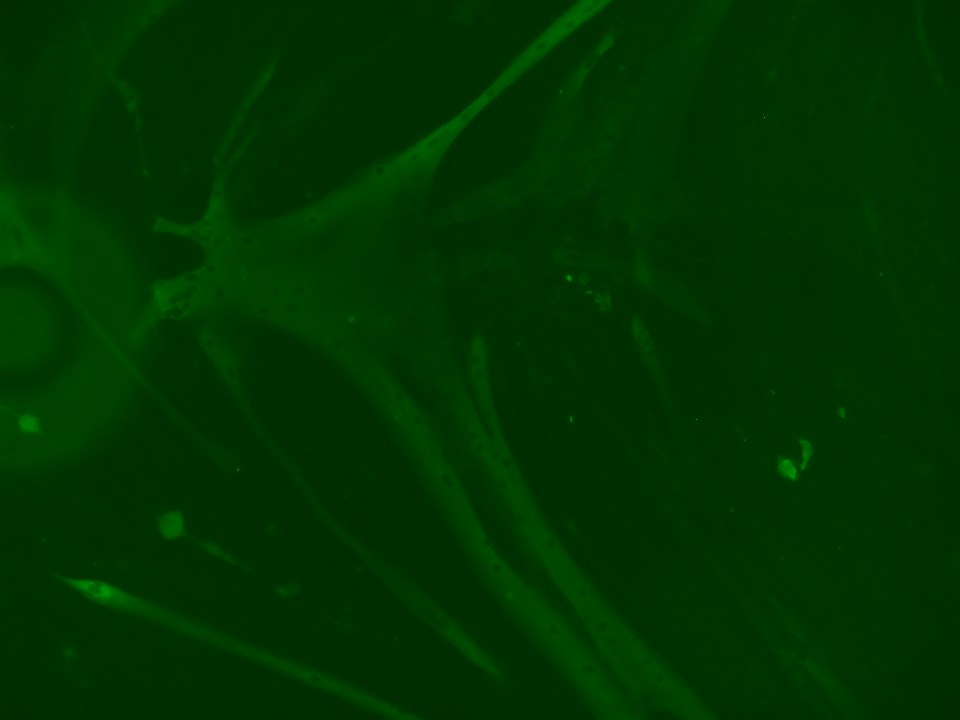

Supplement: Supplementary file 4 — Supplementary Data 1 [file 42003_2022_3423_MOESM4_ESM.zip › Figure S7 (Images)/Raw image files/10x BSC2 P2 BSC-GM/XY08/image_XY08_CH2.tif]

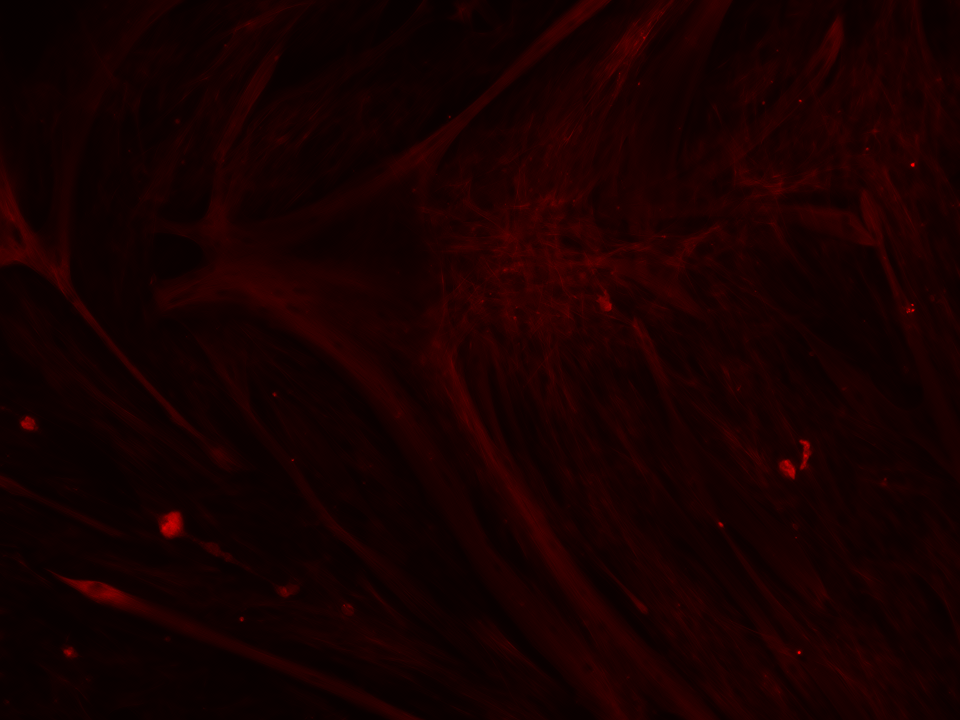

Supplement: Supplementary file 4 — Supplementary Data 1 [file 42003_2022_3423_MOESM4_ESM.zip › Figure S7 (Images)/Raw image files/10x BSC2 P2 BSC-GM/XY08/image_XY08_CH3.tif]

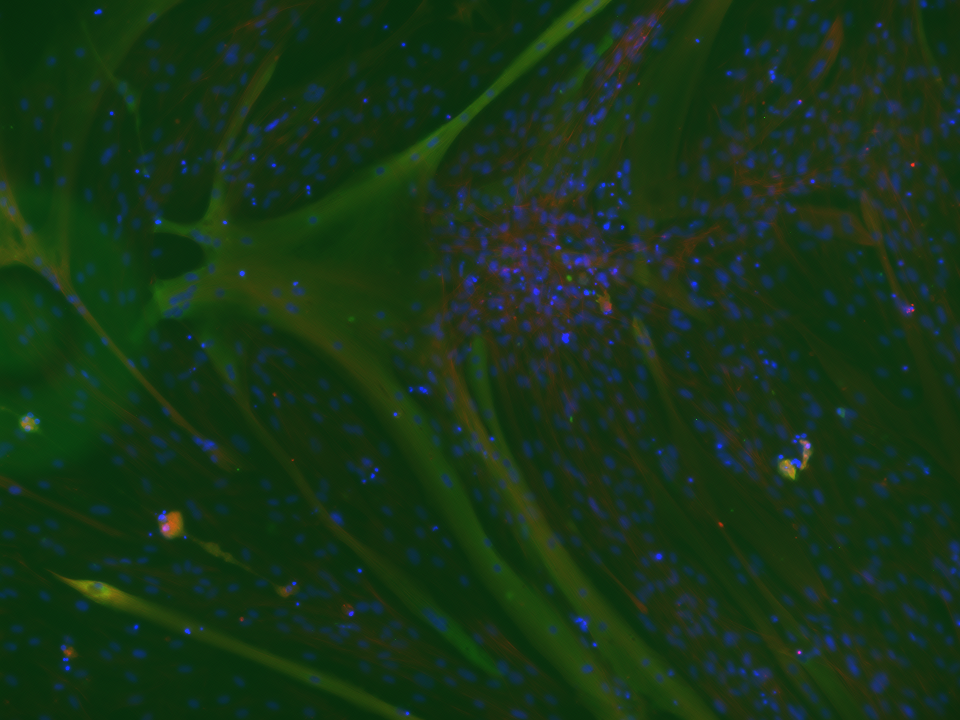

Supplement: Supplementary file 4 — Supplementary Data 1 [file 42003_2022_3423_MOESM4_ESM.zip › Figure S7 (Images)/Raw image files/10x BSC2 P2 BSC-GM/XY08/image_XY08_Overlay.tif]

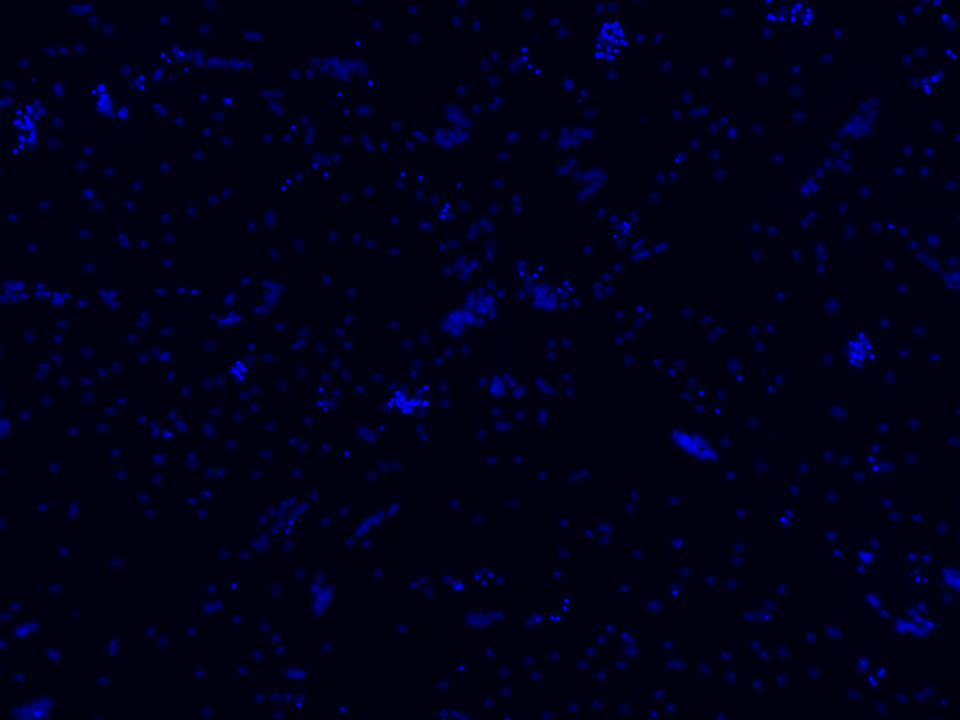

Supplement: Supplementary file 4 — Supplementary Data 1 [file 42003_2022_3423_MOESM4_ESM.zip › Figure S7 (Images)/Raw image files/10x BSC2 P4 Beefy-9 high FGF/XY01/image_XY01_CH1.tif]

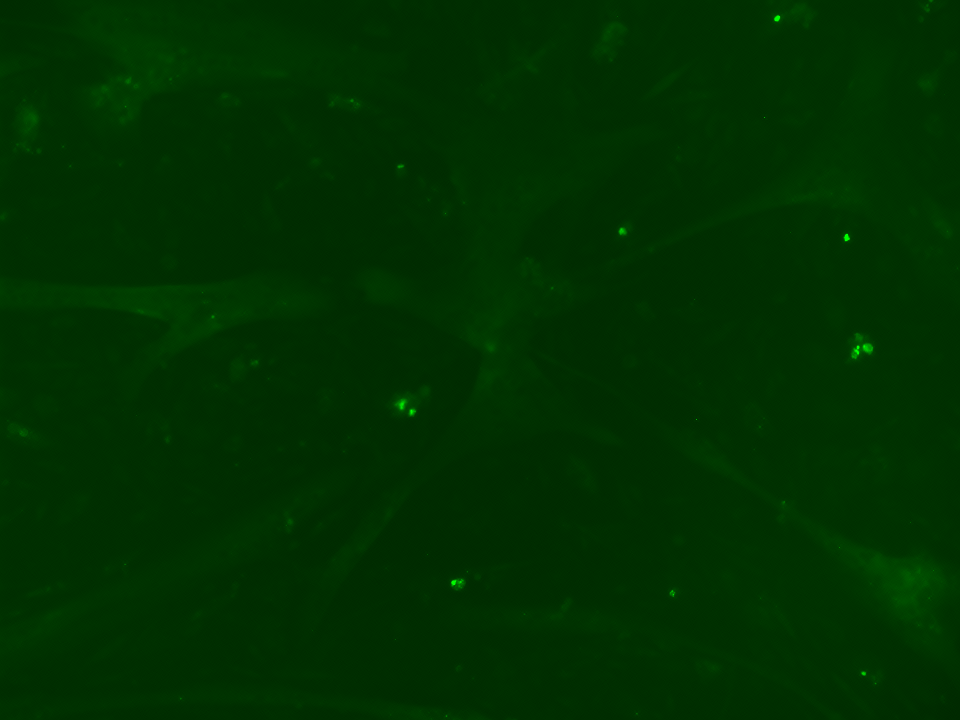

Supplement: Supplementary file 4 — Supplementary Data 1 [file 42003_2022_3423_MOESM4_ESM.zip › Figure S7 (Images)/Raw image files/10x BSC2 P4 Beefy-9 high FGF/XY01/image_XY01_CH2.tif]

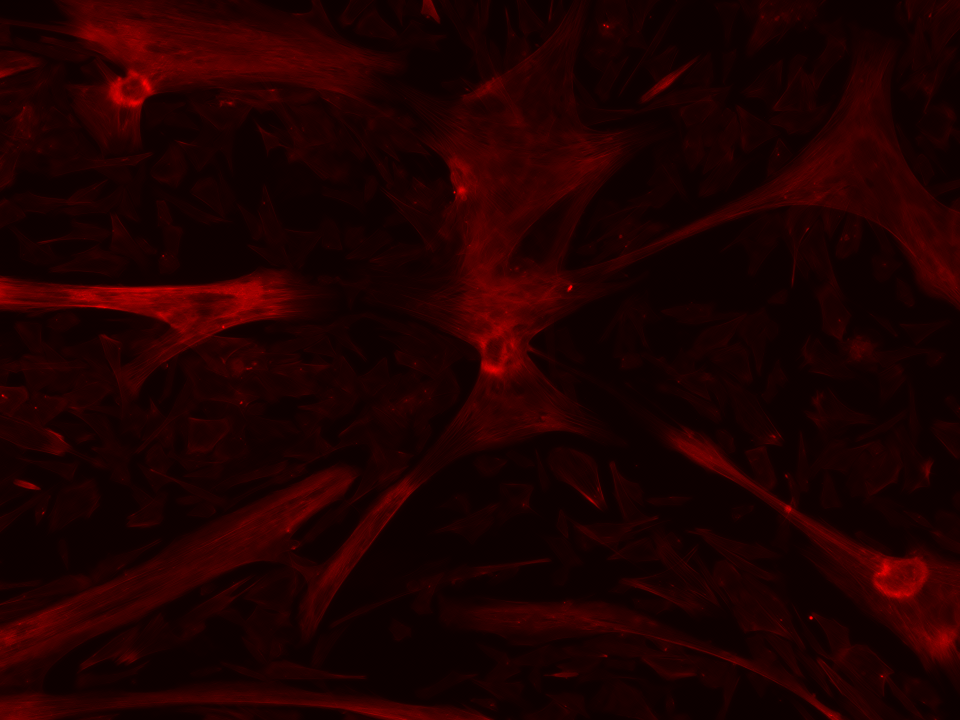

Supplement: Supplementary file 4 — Supplementary Data 1 [file 42003_2022_3423_MOESM4_ESM.zip › Figure S7 (Images)/Raw image files/10x BSC2 P4 Beefy-9 high FGF/XY01/image_XY01_CH3.tif]
