# Supplementary material for: Simple and effective serum-free medium for sustained expansion of bovine satellite cells for cell cultured meat
Source: Commun Biol. 2022 Jun 2;5:466. doi: 10.1038/s42003-022-03423-8 (PMC9163123; doi:10.1038/s42003-022-03423-8)
Supplement: Supplementary file 5 — Reporting Summary [file 42003_2022_3423_MOESM5_ESM.pdf]

nature portfolio

Corresponding author(s): David Kaplan

Last updated by author(s): 4/8/2022

Reporting Summary

Nature Portfolio wishes to improve the reproducibility of the work that we publish. This form provides structure for consistency and transparency in reporting. For further information on Nature Portfolio policies, see our Editorial Policies and the Editorial Policy Checklist.

Statistics

For all statistical analyses, confirm that the following items are present in the figure legend, table legend, main text, or Methods section.

n/a	Confirmed	
☐	☒	The exact sample size (*n*) for each experimental group/condition, given as a discrete number and unit of measurement
☐	☒	A statement on whether measurements were taken from distinct samples or whether the same sample was measured repeatedly
☐	☒	The statistical test(s) used AND whether they are one- or two-sided<br>*Only common tests should be described solely by name; describe more complex techniques in the Methods section.*
☒	☐	A description of all covariates tested
☐	☒	A description of any assumptions or corrections, such as tests of normality and adjustment for multiple comparisons
☐	☒	A full description of the statistical parameters including central tendency (e.g. means) or other basic estimates (e.g. regression coefficient) AND variation (e.g. standard deviation) or associated estimates of uncertainty (e.g. confidence intervals)
☒	☐	For null hypothesis testing, the test statistic (e.g. *F*, *t*, *r*) with confidence intervals, effect sizes, degrees of freedom and *P* value noted<br>*Give P values as exact values whenever suitable.*
☒	☐	For Bayesian analysis, information on the choice of priors and Markov chain Monte Carlo settings
☒	☐	For hierarchical and complex designs, identification of the appropriate level for tests and full reporting of outcomes
☒	☐	Estimates of effect sizes (e.g. Cohen's *d*, Pearson's *r*), indicating how they were calculated

Our web collection on statistics for biologists contains articles on many of the points above.

Software and code

Policy information about availability of computer code

Data collection	BioTek Gen5 Software (for microplate analysis), KEYENCE CV-X (for microscopy), OLYMPUS Provi CM20 Software (for live-cell image analysis)
Data analysis	Graphpad Prism 9.0 software, Microsoft excel, ImageJ (for images).

For manuscripts utilizing custom algorithms or software that are central to the research but not yet described in published literature, software must be made available to editors and reviewers. We strongly encourage code deposition in a community repository (e.g. GitHub). See the Nature Portfolio guidelines for submitting code & software for further information.

Data

Policy information about availability of data

All manuscripts must include a data availability statement. This statement should provide the following information, where applicable:

- Accession codes, unique identifiers, or web links for publicly available datasets
- A description of any restrictions on data availability
- For clinical datasets or third party data, please ensure that the statement adheres to our policy

The authors declare that the data supporting this study are available within the article's Supplementary and source data files. Extra data are available from the corresponding author upon request.

Field-specific reporting

Please select the one below that is the best fit for your research. If you are not sure, read the appropriate sections before making your selection.

☒ Life sciences ☐ Behavioural & social sciences ☐ Ecological, evolutionary & environmental sciences

For a reference copy of the document with all sections, see nature.com/documents/nr-reporting-summary-flat.pdf

Life sciences study design

All studies must disclose on these points even when the disclosure is negative.

Sample size	No sample-size calculations were performed. Sample size was determined to be adequate based on the magnitude and consistency of measurable differences between groups.
Data exclusions	o data was excluded from analysis, except in the case of clear visual aberrations (from bubbles) in images used for fusion index analyses. In this case, excluded data is clearly marked in the provided data files, as are the image files (showing clear disruption of images due to bubbles).
Replication	Attempts at replication were successful.
Randomization	N/A
Blinding	Investigators were not blinded during experimentation. Comparisons reported were not subjective, but rather quantitative analytical values.

Reporting for specific materials, systems and methods

We require information from authors about some types of materials, experimental systems and methods used in many studies. Here, indicate whether each material, system or method listed is relevant to your study. If you are not sure if a list item applies to your research, read the appropriate section before selecting a response.

Materials & experimental systems

n/a	Involved in the study	
☐	☒	Antibodies
☐	☒	Eukaryotic cell lines
☒	☐	Palaeontology and archaeology
☐	☒	Animals and other organisms
☒	☐	Human research participants
☒	☐	Clinical data
☒	☐	Dual use research of concern

Methods

n/a	Involved in the study	
☒	☐	ChIP-seq
☒	☐	Flow cytometry
☒	☐	MRI-based neuroimaging

Antibodies

Antibodies used	Phalloidin 594 (ThermoFisher #A12381); anti-MHC (Developmental studies hybridoma bank #MF-20); anti-mouse (ThermoFisher #A-11001) Anti-rabbit (ThermoFisher #A-11072); anti-Pax7 (ThermoFisher #PA5-68506); DAPI (Abcam #ab104139); BODIPY™ 493/503 (ThermoFisher #D3922); anti-MyoD (ThermoFisher #MA5-12902); anti-Myogenin (Santa Cruz Biotecnology #sc-52903)
Validation	Phalloidin 594 (ThermoFisher #A12381) has been validated in numerous studies. Some PubMed IDs include: 16399995, 11067945, 18347012; anti-MHC (Developmental studies hybridoma bank #MF-20) has been validated in several studies (e.g., doi: 10.2527/jas.2013-7193 & DOI: 10.1083/jcb.95.3.763) and has been used previously in published work by our group (DOI: 10.1016/j.ymben.2020.07.011); DAPI (Abcam #ab104139), routine counter-stain; BODIPY (ThermoFisher #D3922) is likewise routinely used for detecting cellular lipids and has been validated in numerous studies, including for visualizing lipid accumulation in skeletal muscle cells and tissue (DOI: 10.1155/2011/598358 & DOI: 10.1016/j.bbrc.2020.03.025); Anti-rabbit (ThermoFisher #A-11072) has been validated in several studies (e.g., doi: 10.1038/s41467-019-11594-y); anti-Pax7 (ThermoFisher #PA5-68506) has been validated in several studies (e.g., doi: 10.3390/foods8100521); anti-MyoD (ThermoFisher #MA5-12902) has been validated in several studies (e.g., doi: 10.14440/jbm.2018.219); and anti-Myogenin (Santa Cruz Biotecnology #sc-52903) has been validated in several studies (e.g., doi: 10.1186/s12915-021-00980-y)

Eukaryotic cell lines

Policy information about cell lines

Cell line source(s)	Primary bovine satellite cell (BSC) isolations
Authentication	Muscle precursor identity of BSCs was confirmed through differentiation and staining / observation of myotubes

Mycoplasma contamination	BSCs were not tested for mycoplasma, but were cultured for two-weeks in primocin-containing media to eliminate possible mycoplasma contamination following isolation
Commonly misidentified lines (See ICLAC register)	N/A

Animals and other organisms

Policy information about studies involving animals; ARRIVE guidelines recommended for reporting animal research

Laboratory animals	N/A (farm animal used: 14-day-old Simmental bull)
Wild animals	N/A (farm animal used)
Field-collected samples	The bull was kept at the Tufts Cummings School of Veterinary Medicine as part of the school's cattle herd, and was housed prior to and following the tissue biopsy on the 200-acre school farm. The isolation was performed by a Tufts' school veterinarian. Briefly, the cow was be clipped and prepped according to standard surgical procedure over the right paralumbar fossa. An inverted "L" block was performed using 20 cc of 2% lidocaine around the surgical site. A 3 cm full thickness skin and subcutaneous incision was made to access the semitendinosus. A 0.5 cm X 1 cm X 2 cm piece of muscle was removed, and the wound was closed using "0" PDS on the muscle and Polymid (nylon) on the skin in an interrupted pattern. Analgesia was given: Flunixin 1.1 mg/kg IV, with a single injection providing 12 hours of anti-inflammatory action. The cow was then observed for swelling, sensitivity, appetite, attitude and gait for 72 hours following surgery. After the surgery was completed, the animal was returned to normal activities as part of the Tufts herd.
Ethics oversight	Approval was given by the Tufts University Institutional Animal Care and Use Committee (IACUC)

Note that full information on the approval of the study protocol must also be provided in the manuscript.
